# Contrasting E−H Bond Activation Pathways of a Phosphanyl‐Phosphagallene

**DOI:** 10.1002/anie.202109334

**Published:** 2021-08-31

**Authors:** Joey Feld, Daniel W. N. Wilson, Jose M. Goicoechea

**Affiliations:** ^1^ Department of Chemistry University of Oxford Chemistry Research Laboratory 12 Mansfield Rd. Oxford OX1 3TA UK

**Keywords:** decarbonylation, gallium, multiple bonds, phosphaketenes, phosphorus

## Abstract

The reactivity of the phosphanyl‐phosphagallene, [H_2_C{N(Dipp)}]_2_PP=Ga(Nacnac) (Nacnac=HC[C(Me)N(Dipp)]_2_; Dipp=2,6‐^*i*^Pr_2_C_6_H_3_) towards a series of reagents possessing E−H bonds (primary amines, ammonia, water, phenylacetylene, phenylphosphine, and phenylsilane) is reported. Two contrasting reaction pathways are observed, determined by the polarity of the E−H bonds of the substrates. In the case of protic reagents (^δ−^E−H^δ+^), a frustrated Lewis pair type of mechanism is operational at room temperature, in which the gallium metal centre acts as a Lewis acid and the pendant phosphanyl moiety deprotonates the substrates. Interestingly, at elevated temperatures both NH_2_^*i*^Pr and ammonia can react via a second, higher energy, pathway resulting in the hydroamination of the Ga=P bond. By contrast, with hydridic reagents (^δ+^E−H^δ−^), such as phenylsilane, hydroelementation of the Ga=P bond is exclusively observed, in line with the polarisation of the Si−H and Ga=P bonds.

## Graphical abstract

We describe the synthesis and reactivity of a phosphanyl phosphagallene towards reagents with protic and hydridic E−H bonds. These studies reveal that there are two competing reactivity pathways available for such bond activation reactions: a frustrated Lewis pair type of mechanism, and a competing pathway which results in the hydroelementation of the Ga=P double bond.

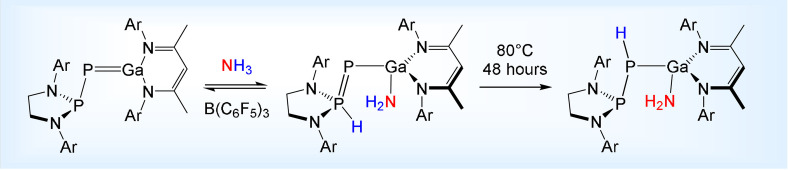

## Introduction

Heteroatomic multiple bonds between the group 13 and 15 elements are of interest due to their valence isoelectronic relationship with C=C and C≡C bonds. The polarity of such bonds, a result of the electronegativity difference between the elements of groups 13 and 15, imparts chemical characteristics to molecules that differ significantly from their carbon‐containing analogues. Examples of compounds with E=E′ bonds in which one element has a principal quantum number (*n*) of 2 (i.e. E=Al, Ga and E′=N; or E=B and E′=P, As) have been known for decades. The synthesis of compounds with B=P and B=As double bonds was pioneered by Nöth and Power who were able to trap such compounds by coordination to Lewis acids and/or bases.[^1^, ^2^] More recently, several other research groups have explored such compounds including the groups of Stephan, Bertrand, Braunschweig and others.[^3^, ^4^, ^5^, ^6^, ^7^] Power and co‐workers also developed a synthetic strategy allowing access to E=N (E=Al, Ga) double bonds by employing a group 13 carbenoid E(Nacnac) (Nacnac=HC[C(Me)N(Dipp)]_2_; Dipp=2,6‐^*i*^Pr_2_C_6_H_3_)^[8]^ and sterically encumbered organic azides, which liberate N_2_ to give the desired double bond.[^9^, ^10^] A similar strategy was recently used to allow access to aluminium‐imides starting from organic azides aluminium(I) reagents.[^10d^, ^10e^, ^10f^]

**Figure 1:**
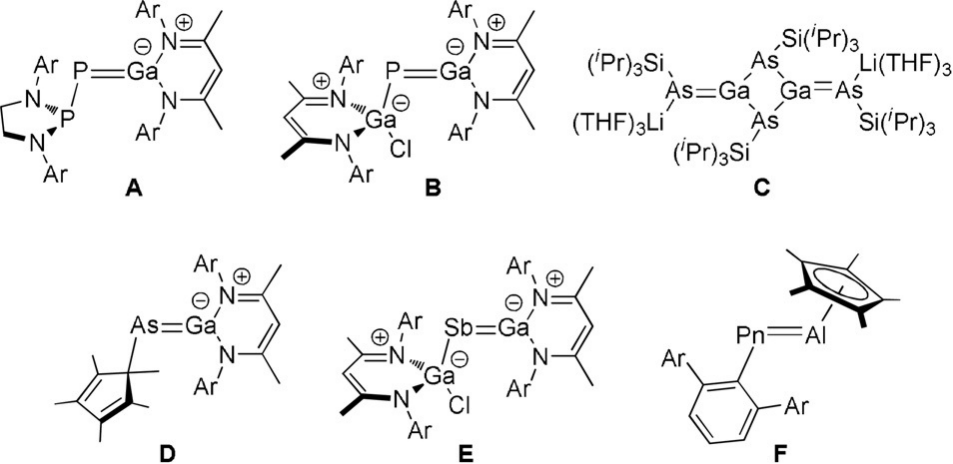
Previously reported examples of heteroatomic group 13/15 multiple bonds. Ar=2,6‐^*i*^Pr_2_C_6_H_3_; Pn=P, As.

Heteroatomic multiple bonds between heavy group 13/15 elements are more unusual due to poorer orbital overlap on descending the group, resulting in a propensity for these species to oligomerise. Last year, we reported the first example of a phosphagallene (Figure 1, **A**) by exploiting the known reactivity of phosphanyl‐phosphaketenes to eliminate CO in the presence of strong nucleophiles,^[11]^ in this case the group 13 carbenoid Ga(Nacnac). This strategy is widely applicable, and has been used by our group and Schulz and co‐workers to expand on this class of compounds affording other phosphagallenes (Figure 1, **B**).[^12^, ^13^] A compound containing a Ga=As double bond was reported by von Hänisch and Hampe in the form of a dimeric [{Li(THF)_3_}_2_Ga_2_{As(Si^*i*^Pr_3_)}_4_] (Figure 1, **C**) which was obtained by the reaction of GaCl_3_ with two equivalents of Li_2_As(Si^*i*^Pr_3_).^[14]^ More recently, Schulz and co‐workers reported the synthesis of monomeric gallaarsenes and gallastibenes (Figure 1, **D** and **E**, respectively).[^15^, ^16^] This series of interesting compounds was expanded to include examples of Al=P and Al=As double bonds by Braunschweig and Hering‐Junghans, who were able to access such species by reaction of (AlCp*)_4_ with the phospha‐Wittig reagents ^Dipp^TerPnPMe_3_ (Pn=P, As).^[17]^

Despite the recent availability of compounds containing Ga=Pn and Al=Pn bonds (Pn=P, As), their reactivity remains largely unexplored. In contrast, species with homoatomic multiple bonds such as digermynes,^[18]^ diborynes,^[19]^ and dialumenes,^[20]^ for example, have been shown to activate H_2_. For heteroatomic multiple bonds, the polarity difference is anticipated to further favour heterolytic cleavage of inert bonds. Anionic aluminium‐imides have been shown to add H_2_ across the Al=N bond.^[10e]^ Kinjo has also recently shown that H_2_ addition across a Ge=B double bond is possible.^[21]^ It is worth noting that these examples all contain heteroatomic multiple bonds in which one of the elements has an *n*=2. We sought to map the reactivity of highly polarized heavier element multiple bonds by exploring the reactivity of compound **A** towards a range of compounds possessing E−H bonds (E=N, O, C, P, Si) of different polarity. The results of our studies are described herein, and show contrasting reactivity with bond activation reactions involving either a frustrated Lewis pair type mechanism or addition across the Ga=P π‐bond. To the best of our knowledge this is the first example of a heteroatomic multiple bond which exhibits such contrasting reactivity.

## Results and Discussion

We recently demonstrated that the phosphanyl‐phosphagallene, **A**, is capable of reacting as a frustrated Lewis pair (FLP) towards apolar substrates such as dihydrogen and carbon dioxide.^[11]^ In the case of dihydrogen, heterolytic H−H bond activation was observed involving the pendant phosphanyl moiety which acts as the Lewis basic component. In the case of carbon dioxide, the molecule can be trapped between the phosphanyl moiety and the gallium center, affording a five‐membered ring, as observed previously for other intramolecular FLPs such as those reported by Tamm, Uhl, Fontaine, and others.[^22^, ^23^, ^24^, ^25^, ^26^] These studies demonstrated that the pendant phosphanyl moiety plays a pivotal role in the reactivity of **A**, which contrasts with that observed for other phosphagallenes that lack a pendant Lewis basic moiety such as compound **B**. In the case of the latter, two equivalents of carbon dioxide were found to insert between the Ga=P bond.^[13]^ These observations prompted us to explore the reactivity of **A** towards substrates with polar E−H bonds.

**Scheme 1:**
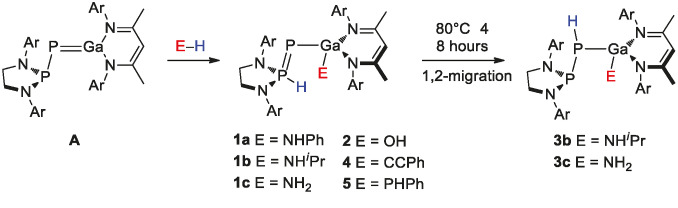
Reactivity of **A** towards primary amines, ammonia, water, phenylacetylene and phenylphosphine. Ar=2,6‐^*i*^Pr_2_C_6_H_3_.

We started by exploring the reactivity of **A** towards amines. Reactions of **A** with aniline and isopropylamine resulted in immediate, quantitative formation of **1 a** and **1 b**, respectively, as indicated by decolourisation of the solution upon mixing (Scheme 1).^[27]^ In the ^31^P{^1^H} NMR spectra, resonances were found at a significantly lower frequency relative to **A** (**1 a**: 62.2, −243.6 ppm; **1 b**: 60.6, −238.1 ppm). This was accompanied by an increase of the ^1^ *J*_P‐P_ coupling constant from 346 Hz to 574 and 572 Hz, respectively, implying an increase of P−P bond order (the pendant phosphanyl group is transformed into a phosphorane). The proton coupled ^31^P NMR spectrum displays a ^1^ *J*_P‐H_ coupling for the phosphorane phosphorus atom (**1 a**: 457 Hz; **1 b**: 450 Hz), a clear indication of protonation. The corresponding resonance in the ^1^H NMR is found as a doublet at a high chemical shift (**1 a**: 9.13 ppm, **1 b**: 9.35 ppm). These correspond to a [1,3] activation via the phosphanyl phosphorus lone pair similar to the H_2_ activation reaction we have previously reported.^[11]^ Contrastingly, secondary amines diisopropylamine and diphenylamine did not react, even when heated to 80 °C for 2 hours.

Ammonia is a more challenging substrate than primary amines. In the context of main group compounds, N−H bond activation of ammonia is limited to low valent main group species,[^28^, ^29^, ^30^, ^31^, ^32^] and geometrically constrained T‐shaped phosphorus heterocycles.[^33^, ^34^] To our knowledge, the only example of FLP‐facilitated NH_3_ activation is an intermolecular pair which involves an N‐heterocyclic carbene and tris(pentafluorophenyl)borane.^[35]^ When a solution of **A** in C_6_D_6_ was exposed to 1 bar of ammonia, the solution immediately decolourised and two new signals in the ^31^P{^1^H} NMR spectrum were observed at 62.0 and −252.1 ppm (^1^ *J*_P−P_=566 Hz) corresponding to the ammonia activation product **1 c**. The ^1^H NMR spectrum displayed a broad resonance at −0.38 ppm, consistent with an Ga−NH_2_ moiety, as seen in related complexes of the type (Nacnac)Ga(NH_2_)R, which exhibit NH_2_ resonances at 0.07 (R=^*t*^Bu) and −0.58 ppm (R=NH_2_).[^32d^, ^36^] A second, minor product could also be observed in the ^31^P{^1^H} NMR spectrum with a pair of resonances at 61.8 and −255.5 ppm (^1^ *J*_P−P_=560 Hz). Both the major and minor products exhibited ^1^ *J*_P−H_ coupling of the phosphorane resonance, indicating a [1,3] type activation.

The minor product was identified as the hydrolysis product **2**, which forms due to the presence of traces amounts of water in the ammonia. **2** was independently synthesized by addition of one equivalent of H_2_O to **A** and fully characterised (see SI for details). Similarly, addition of D_2_O to **A** results in a characteristic equal intensity triplet in the ^31^P{^1^H} NMR spectrum (^1^ *J*_P−D_=72 Hz) resulting from P−D coupling (^2^H: *I*=1).

**Figure 2:**
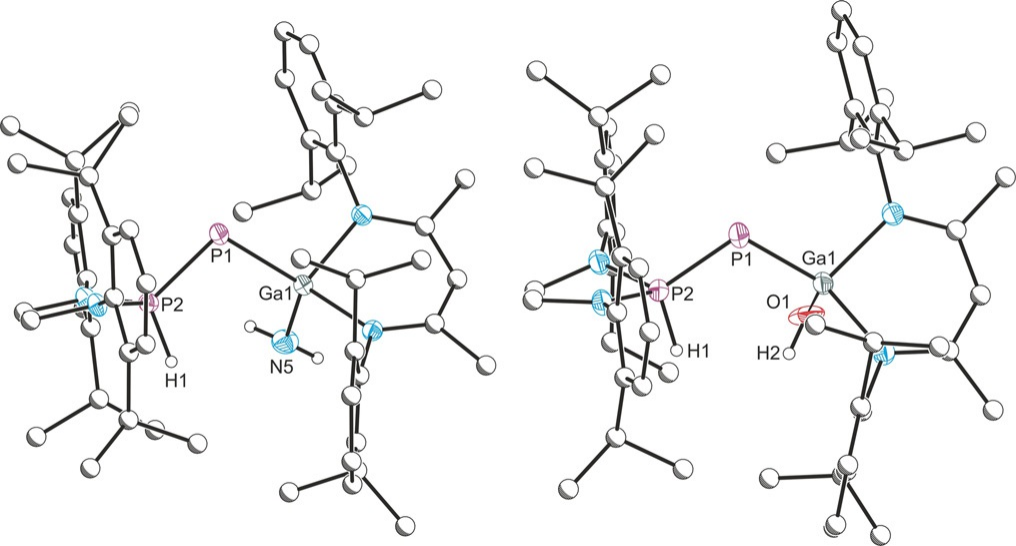
Molecular structure of **1 c** (left) and **2** (right). Ellipsoids set at 50 % probability; hydrogen atoms (with the exception of those originating from the substrate) omitted for clarity. All carbon atoms are pictured as spheres of arbitrary radius. Selected interatomic distances [Å] and angles [°]: **1 c**: Ga1–P1 2.304(1), P1–P2 2.041(1), Ga1–N5 1.845(2), P2–H1 1.31(2); Ga1‐P1‐P2 100.27(3). **2**: Ga1–P1 2.301(1), P1–P2 2.042(1), Ga1–O1 1.946(3), P2–H1 1.33(3); Ga1‐P1‐P2 101.46(3).

Crystals of **1 a**–**1 c** and **2** suitable for X‐ray diffraction were grown from hexane solutions at room temperature (Figure 2; see SI for the structures of **1 a** and **1 b**). The P1−P2 distances in **1 c** (2.041(1) Å) and **2** (2.042(1) Å) are notably shorter when compared to **A** (2.212(1) Å), which is consistent with an increase in the bond order between the phosphorus atoms (this is accompanied by an elongation of the Ga−P bond by 0.12 Å).

Heating a benzene solution of either **1 b** or **1 c** at 80 °C for two days yielded new signals in the ^31^P{^1^H} NMR spectrum, assigned as **3 b** (150.7 ppm, −187.8 ppm; ^1^ *J*_P−P_=234 Hz) and **3 c** (150.5 ppm, −191.3 ppm; ^1^ *J*_P−P_=239 Hz), respectively. In both cases, the ^1^ *J*_P−P_ constants are smaller than those of **1 b** and **1 c**, consistent with a reduction in the bond order for the P−P bond. In the proton‐coupled ^31^P NMR spectrum, the phosphorane resonance has lost its P−H coupling and instead the P−H coupling is found for the former phosphanylidene atom [^1^ *J*_P−H_=169.0 Hz (**3 b**); 171.8 Hz (**3 c**)], indicating a [1,2] proton migration. Crystals suitable for single crystal X‐ray diffraction were obtained for both **3 b** and **3 c**. However, in the latter case positional disorder across an inversion centre prevents meaningful discussion of bond parameters.

**Figure 3:**
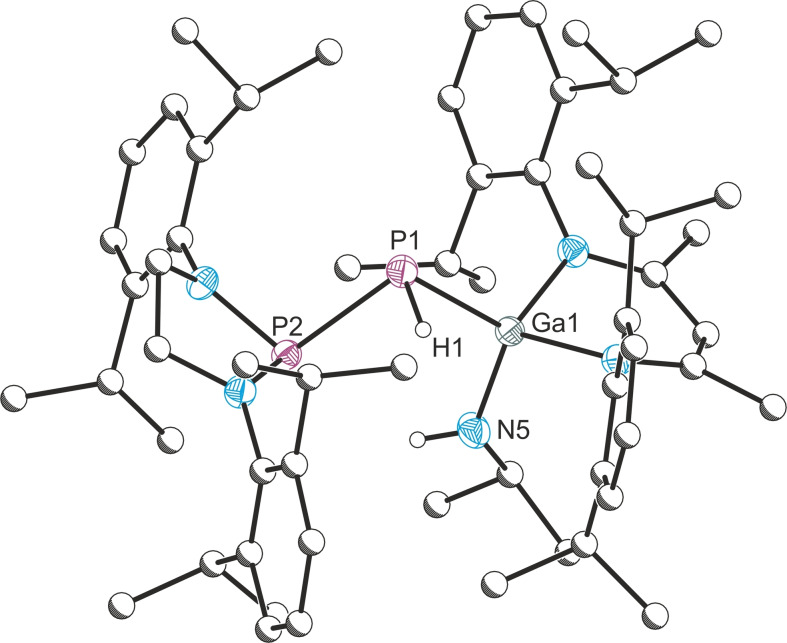
Molecular structure of **3 b**. Ellipsoids set at 50 % probability; hydrogen atoms (with the exception of those originating from the substrate) omitted for clarity. All carbon atoms are pictured as spheres of arbitrary radius. Selected interatomic distances [Å] and angles [°]: Ga1–P1 2.3358(5), P1–P2 2.2999(6), Ga1–N5 1.8623(16), P1–H1 1.31(3); Ga1‐P1‐P2 97.50(2).

The crystal structure of **3 b** (Figure 3) confirms migration of the proton position to the central phosphanylidene phosphorus atom to afford a secondary phosphine. The most notable structural change is an elongation of the P1−P2 bond, 2.300(1) Å, relative to that of **1 b** (2.067(1) Å).

DFT calculations were performed at the B3LYP/def2TZVP(Ga,P,N)/ Def2SVP(C,H) level of theory with the ligand diisopropylphenyl groups truncated to phenyl substituents (See Figure S36). Adduct formation between the gallium atom of **A^DFT^** and NH_3_ to form **I1** is barrierless and slightly exergonic (Δ*G*=−2 kcal mol^−1^; within error of the calculation). Direct deprotonation of ammonia by either P1 or P2 of **A^DFT^** results in high energy intermediates (>400 kcal mol^−1^), ruling out a mechanism in which deprotonation precedes adduct formation. Following formation of **I1**, N−H deprotonation by P2 to yield **1 c^DFT^** is exergonic by 4 kcal mol^−1^, with an associated free energy barrier of 15.7 kcal mol^−1^. This indicates that conversion between **I1** and **1 c^DFT^** is facile at room temperature. Similarly, the reverse reaction is also feasible at room temperature (Δ*G* ^≠^=19.7 kcal mol^−1^), implying that these two species may be in equilibrium in solution. We were unable to find a reasonable transition state between **1 c^DFT^** and **3 c^DFT^** due to the requirement for rotation about the sterically congested P=P bond, making this an energetically expensive process. However, conversion of **I1** to **3 c^DFT^** has an associated barrier of 27.5 kcal mol^−1^, consistent with the experimental observation that **3 c** only forms upon heating to 80 °C.

**Scheme 2:**
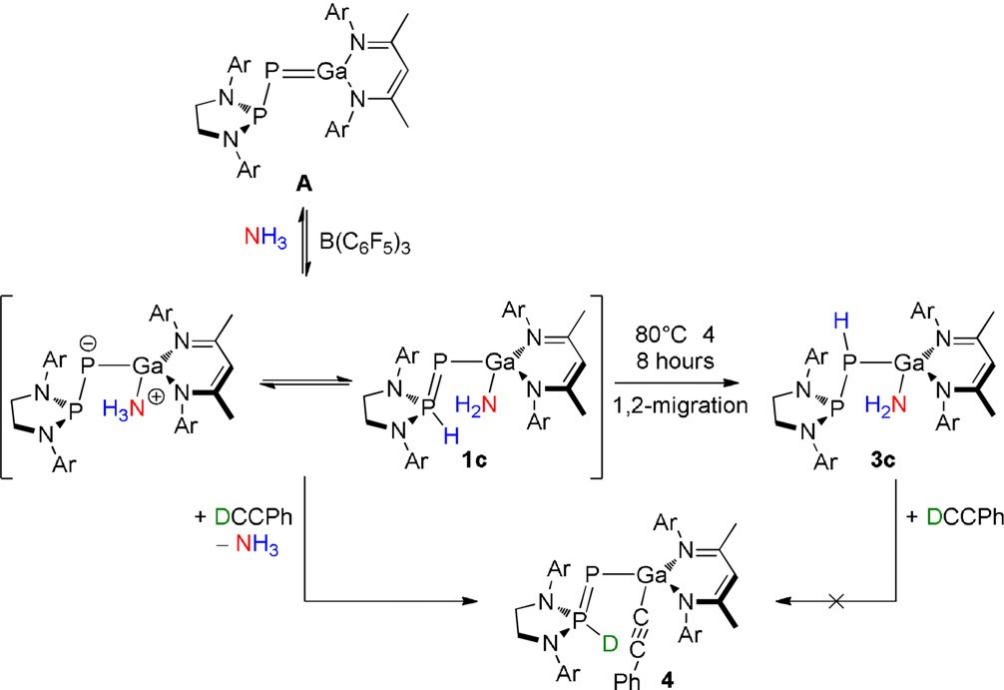
Reactivity of **A** towards ammonia to afford **1 c** and subsequent reactivity of this species. Ar=2,6‐^*i*^Pr_2_C_6_H_3_.

Encouraged by the computational studies, we sought to investigate the possibility of reversible NH_3_ activation by this system. Placing **1 c** under a dynamic vacuum at room temperature did not result in reformation of **A**, and heating a solid sample under vacuum resulted in proton migration to yield **3 c**. Instead, we turned our attention to chemical processes. Addition of one equivalent of PhCCH to **1 c** immediately yields **4** (Scheme 2), as indicated by a new set of doublet signals in the ^31^P{^1^H} NMR spectrum (70.9 ppm, −236.5 ppm; ^1^ *J*_P−P_=567 Hz). **4** could also be prepared directly through addition of PhCCH to **A**. In order to rule out a σ‐bond metathesis mechanism for the conversion of **1 c** to **4**, deuterium labelling experiments were performed. Addition of a stoichiometric amount of PhCCD to **1 c** yields signals in the ^31^P NMR spectrum similar to **4** (70.2 ppm, −238.3 ppm; ^1^ *J*_P−P_=568 Hz), however the phosphorane resonance now exhibits a distinctive P−D coupling pattern (^1^ *J*_P−D_=72 Hz). This implies that, firstly ammonia activation is reversible, as σ‐bond metathesis would not yield a P−D bond, and secondly that there is no H/D exchange with PhCCD. Further, addition of the Lewis acid tris(pentafluorophenyl)borane (BCF) to **1 c** immediately causes a change from a colourless solution to orange. The ^31^P NMR spectrum of the solution indicates that **A** has reformed, indicating the abstraction of NH_3_ to form the Lewis acid‐base adduct, H_3_N:B(C_6_F_5_)_3_.

Phenylphosphine was also found to react with **A** to form **5**. Three resonances were found in the ^31^P{^1^H} NMR spectrum, corresponding to the phosphorane, phosphanylidene and phosphine nuclei (60.1, −109.7 and −210.8 ppm, respectively). All NMR data are consistent with a [1,3] activation as observed for amines. The P−P and P−H coupling constants are comparable with compounds **1 a**–**c** and **2**. The structure of **5** was confirmed by single crystal X‐ray diffraction (see SI for further details).

**Scheme 3:**
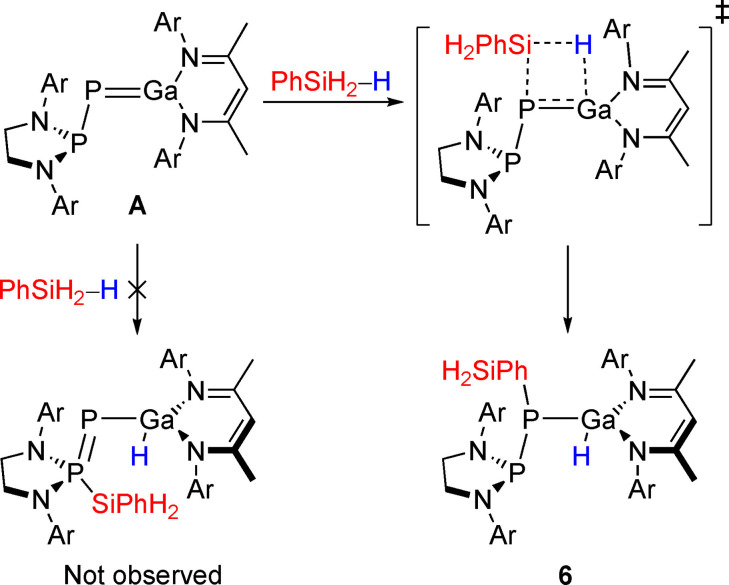
Reactivity of **A** towards phenylsilane. Ar=2,6‐^*i*^Pr_2_C_6_H_3_.

With the results in hand, we turned our attention to compounds with hydridic E−H bonds. The activation of hydridic substrates by FLPs results in the formation of a terminal hydride on the Lewis acidic site, in accordance with the polarity of the ^δ+^E−H^δ−^ bond.^[37]^ Addition of phenylsilane to a solution of **A** (Scheme 3) resulted in quantitative formation of a new product, **6**, as indicated by two doublet resonances at 162.5 and −173.6 ppm (^1^ *J*_P−P_=389 Hz) in the ^31^P NMR spectrum. The P−P coupling constant is similar to **A**, indicative of a P−P single bond. The broadened signals had no discernible long‐range P−H coupling constants and did not change upon proton decoupling. The ^1^H NMR spectrum displayed a sharp doublet resonance at 5.22 ppm (^2^ *J*_P−H_=6 Hz), corresponding to the SiH_2_ moiety with ^29^Si satellites (^1^ *J*_Si−H_=101 Hz), the Ga−H is observed as a broadened doublet 5.86 ppm (^2^ *J*_P−H_=31 Hz). No signal was observed in the ^29^Si NMR spectrum, but by using ^1^H/^29^Si HMBC, a signal at −36.0 ppm was found to correlate to the SiH_2_ protons.

**Figure 4:**
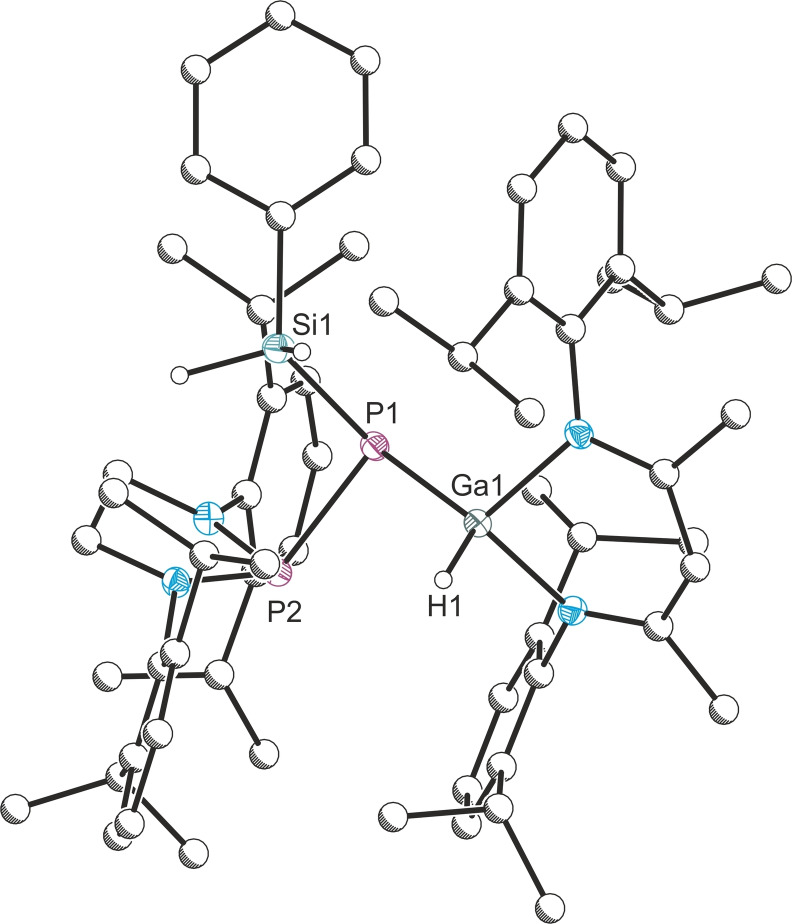
Molecular structure of **6**. Ellipsoids set at 50 % probability; hydrogen atoms (with the exception of those originating from the H_3_SiPh substrate) omitted for clarity. All carbon atoms are pictured as spheres of arbitrary radius. Selected interatomic distances [Å] and angles [°]: Ga1–P1 2.341(1), P1–P2 2.285(1), Ga1–H1 1.42(2), P1–Si1 2.239(1); Ga1‐P1‐P2 102.73(2).

Crystals of **6** suitable for X‐ray diffraction were grown from hexane solutions at room temperature. The asymmetric unit contains two crystallographically independent molecules, for clarity, only one of these is discussed. The solid state structure revealed the [1,2] activation product, with an association of the phenylsilyl group to the phosphanylidene atom, with a P−Si bond distance of 2.239(1) Å (Figure 4). The P−P distance 2.285(1) Å is significantly longer than the P−P distance found in the [1,3] amine activation reactions (2.041(1)–2.067(1) Å), in the range of a P−P single bond. The P−Ga bond distance is slightly longer at 2.341(1) Å than in **A** (2.018(1) Å), indicating the change from a double bond to a single bond.

DFT calculations (Figure S37) predict **6^DFT^** to be more stable than the starting material by 17.3 kcal mol^−1^ and the [1,3] isomer by 16.7 kcal mol^−1^. Further, access to **6^DFT^** is associated with a lower free energy barrier (Δ*G* ^≠^=18.8 kcal mol^−1^) than its isomer (Δ*G* ^≠^=24.0 kcal mol^−1^). The transition state (**TS3**) indicates concerted formation of both the Ga−H and P−Si bonds, consistent with a σ‐bond metathesis pathway.

## Conclusion

In conclusion, we have demonstrated the ability for phosphanyl phosphagallene **A** to activate polar E−H bonds at room temperature. The Ga/P FLP‐type reactivity is able to capture and cleave the E−H bond in amines, phosphines and terminal alkynes. Most notably, the activation of ammonia is facile at 1 bar pressure and could be reversed using a Lewis acid. Heating the ammonia activated product resulted in proton migration to yield a secondary phosphine. Work is ongoing on optimising the design of this system to allow for insertion of substrates into the Ga−NH_2_ bond, with the aim of hydroamination directly from NH_3_. Finally, reactivity across the Ga=P π‐bond was observed upon reaction of **A** with phenylsilane to yield exclusively the [1,2] addition product via a σ‐bond metathesis.

## Conflict of interest

The authors declare no conflict of interest.

## Supporting information

As a service to our authors and readers, this journal provides supporting information supplied by the authors. Such materials are peer reviewed and may be re‐organized for online delivery, but are not copy‐edited or typeset. Technical support issues arising from supporting information (other than missing files) should be addressed to the authors.

Supporting Information
