# Supplementary material for: Contrasting E−H Bond Activation Pathways of a Phosphanyl‐Phosphagallene
Source: Angew Chem Int Ed Engl. 2021 Aug 31;60(40):22057–61. doi: 10.1002/anie.202109334 (PMC8518045; doi:10.1002/anie.202109334)
Supplement: Supplementary file 1 — Supporting Information [file ANIE-60-22057-s001.pdf]

Angewandte Chemie
Eine Zeitschrift der Gesellschaft Deutscher Chemiker

Supporting Information

Contrasting E–H Bond Activation Pathways of a Phosphanyl-Phosphagallene

*Joey Feld, Daniel W. N. Wilson, and Jose M. Goicoechea**

anie_202109334_sm_miscellaneous_information.pdf

Contents

1. Experimental Section 2

1.1. General synthetic methods 2

1.2. Synthesis of $[CH_2\{N(Dipp)\}]_2P(H)PGa(NHPh)(NacNac)$ (**1a**) 3

1.3. Synthesis of $[CH_2\{N(Dipp)\}]_2P(H)PGa(NH^iPr)(NacNac)$ (**1b**) 5

1.4. $[CH_2\{N(Dipp)\}]_2P(H)PGa(NH_2)(NacNac)$ (**1c**) 8

1.5. $[CH_2\{N(Dipp)\}]_2P(H)PGa(OH)(NacNac)$ (**2**) 11

1.6. Synthesis of $[CH_2\{N(Dipp)\}]_2PP(H)Ga(NH^iPr)(NacNac)$ (**3**b) 13

1.7. Synthesis of $[CH_2\{N(Dipp)\}]_2PP(H)Ga(NH_2)(NacNac)$ (**3c**) 16

1.9. Synthesis of $[CH_2\{N(Dipp)\}]_2P(H)PGa(CCPh)(NacNac)$ (**4**) 18

1.8. Synthesis of $[CH_2\{N(Dipp)\}]_2P(H)PGa(PHPh)(NacNac)$ (**5**) 21

1.10. Reaction of **1c** with PhCCD 23

1.11. Reaction of **1c** with $B(C_6F_5)_3$ 24

1.12. Synthesis of $[CH_2\{N(Dipp)\}]_2PP(H)Ga(H_2SiPh)(NacNac)$ (**6**) 25

2. Single crystal X-ray diffraction data 29

3. Computational Details 36

4. References 51

1. Experimental Section

1.1. General synthetic methods

All reactions and product manipulations were carried out under an inert atmosphere of argon or dinitrogen using standard Schlenk-line or glovebox techniques (MBraun UNIlab glovebox maintained at <0.1 ppm H_2O and <0.1 ppm O_2). **A** was synthesized according to previously reported synthetic procedure.[1] Hexane (hex; Sigma Aldrich, HPLC grade) and toluene (Sigma Aldrich, HPLC grade) were purified using an MBraun SPS-800 solvent system. C_6D_6 (Aldrich, 99.5%) was dried over CaH_2 and degassed prior to use. All dry solvents were stored under argon in gas-tight ampoules. All solvents were stored over 3Å molecular sieves. $PhNH_2$ (Aldrich, >99.5%), ${}^{i}PrNH_2$ (Alfa Aesar, 99+%), PhCCH (Aldrich, 98%), $PhSiH_3$ (Chem Cruz, 97%) were stored over 3 Å molecular sieves without further purification. $PhPH_2$ in hexanes w/w 10% (Strem, 99%) and NH_3 (BOC, 99.98%) were used as purchased without further purification.

Additional characterization techniques: NMR spectra were acquired on a Bruker AVIII 500 MHz NMR spectrometer (${}^{1}H$ 500 MHz, ${}^{13}C$ 126 MHz) and Bruker AVIII 400 MHz NMR spectrometer (${}^{1}H$ 400 MHz, ${}^{31}P$ 162 MHz, ${}^{11}B$ 128 MHz, ${}^{19}F$ 470 MHz). ${}^{1}H$ and ${}^{13}C$ NMR spectra were referenced to the most downfield solvent resonance (${}^{1}H$ NMR C_6D_6: $\delta = 7.16$ ppm; ${}^{13}C$ NMR C_6D_6: $\delta = 188.06$ ppm). ${}^{31}P$ spectra were externally referenced to an 85% solution of H_3PO_4 in H_2O. Elemental analyses were carried out by Elemental Microanalyses Ltd. (Devon, U.K.). Samples (approx. 6 mg) were submitted in sealed Pyrex ampoules.

1.2. Synthesis of [CH_2\{N(Dipp)\}]$_2$P(H)PGa(NHPh)(NacNac) (1a)

A (50 mg, 0.054 mmol) was dissolved in toluene and a drop of aniline (0.01 mL, 0.110 mmol) was added. The mixture immediately decolourised and the solvent was removed under reduced pressure. Recrystallisation from hexane gave off-white crystalline solids. (0.054 g, 98.2%). Anal. calculated for $C_{61}H_{86}GaN_5P_2$: C, 71.76; H, 8.49; N, 6.86. Found: C, 71.17; H, 8.19; N, 7.32. **^{1}H NMR (400 MHz, C_6D_6):** δ (ppm) 9.13 (dd, $^1J_{P–H}$ = 451.7, $^2J_{P–H}$ = 9.1 Hz, 1H; P*H*), 7.31–7.04 (m, 12H; ArC*H*), 7.02–6.98 (m, 4H; ArC*H*), 6.71 (tt, $^3J_{H–H}$ = 7.2 Hz, $^4J_{H–H}$ = 1.0 Hz 1H; *p*-ArC*H*), 4.98 (s, 1H; NacNac γ-H), 3.83 (sept, $^3J_{H–H}$ = 6.8 Hz, 2H; \{C*H*(CH_3)$_2$\}), 3.47–3.15 (m, 8H; \{C*H*(CH_3)$_2$\} and \{(NCH_2\}$_2$\}), 3.09 (sept, $^3J_{H–H}$ = 6.7 Hz, 2H; \{C*H*(CH_3)$_2$\}), 2.56 (s, 1H; NH), 1.40 (s, 6H; NacNac C*H*$_3$), 1.22 (d, $^3J_{H–H}$ = 6.7 Hz, 6H; Dipp \{CH(C*H*$_3$)$_2$\}), 1.14–1.03 (m, 30H; Dipp \{CH(C*H*$_3$)$_2$\}), 0.99 (d, $^3J_{H–H}$ = 6.8 Hz, 6H; Dipp \{CH(C*H*$_3$)$_2$\}), 0.86–0.84 (m, 6H; Dipp \{CH(C*H*$_3$)$_2$\}). **^{13}C\{^{1}H\} NMR (126 MHz, C_6D_6):** δ (ppm) 169.00 (Ar*C*), 152.38 (Ar*C*), 150.75 (Ar*C*), 149.14 (Ar*C*), 145.19 (Ar*C*), 143.77 (Ar*C*), 142.86 (Ar*C*), 137.89 (Ar*C*), 135.70 (Ar*C*), 135.66 (Ar*C*), 129.33 (Ar*C*), 128.57 (Ar*C*), 128.48 (Ar*C*), 128.35 (Ar*C*), 127.18 (Ar*C*), 125.70 (Ar*C*), 125.30 (Ar*C*), 124.77 (Ar*C*), 124.08 (Ar*C*), 123.80 (Ar*C*), 114.32 (Ar*C*), 96.98 (NacNac *C*H), 51.32 (\{(N*C*H_2)$_2$\}), 51.27 (\{(N*C*H_2)$_2$\}), 29.55 (d, $^4J_{P–C}$ = 7.1 Hz; Dipp \{*C*H(CH_3)$_2$\}), 29.30 (Dipp \{*C*H(CH_3)$_2$\}), 29.14 (d, $^4J_{P–C}$ = 5.3 Hz; Dipp \{*C*H(CH_3)$_2$\}), 27.15 (Dipp \{*C*H(CH_3)$_2$\}), 26.73 (Dipp \{CH(*C*H_3)$_2$\}), 26.35 (Dipp \{CH(*C*H_3)$_2$\}), 26.03 (Dipp \{CH(*C*H_3)$_2$\}), 24.95 (Dipp \{CH(*C*H_3)$_2$\}), 24.84(d, $^5J_{P–C}$ = 7.9 Hz; Dipp \{CH(*C*H_3)$_2$\}) , 24.57 (Dipp \{CH(*C*H_3)$_2$\}), 24.52 (Dipp \{CH(*C*H_3)$_2$\}), 24.04 (Nacnac *C*H_3), 24.02 (Dipp \{CH(*C*H_3)$_2$\}). **^{31}P NMR (162 MHz, C_6D_6):** δ (ppm) 60.61 (dd, $^1J_{P–P}$ = 571.2 Hz, $^1J_{P–H}$ = 450.3 Hz, $^4J_{P–H}$ = 6.8 Hz), −238.12 (d, $^1J_{P–P}$ = 571.1 Hz, $^3J_{P–H}$ = 8.8 Hz).

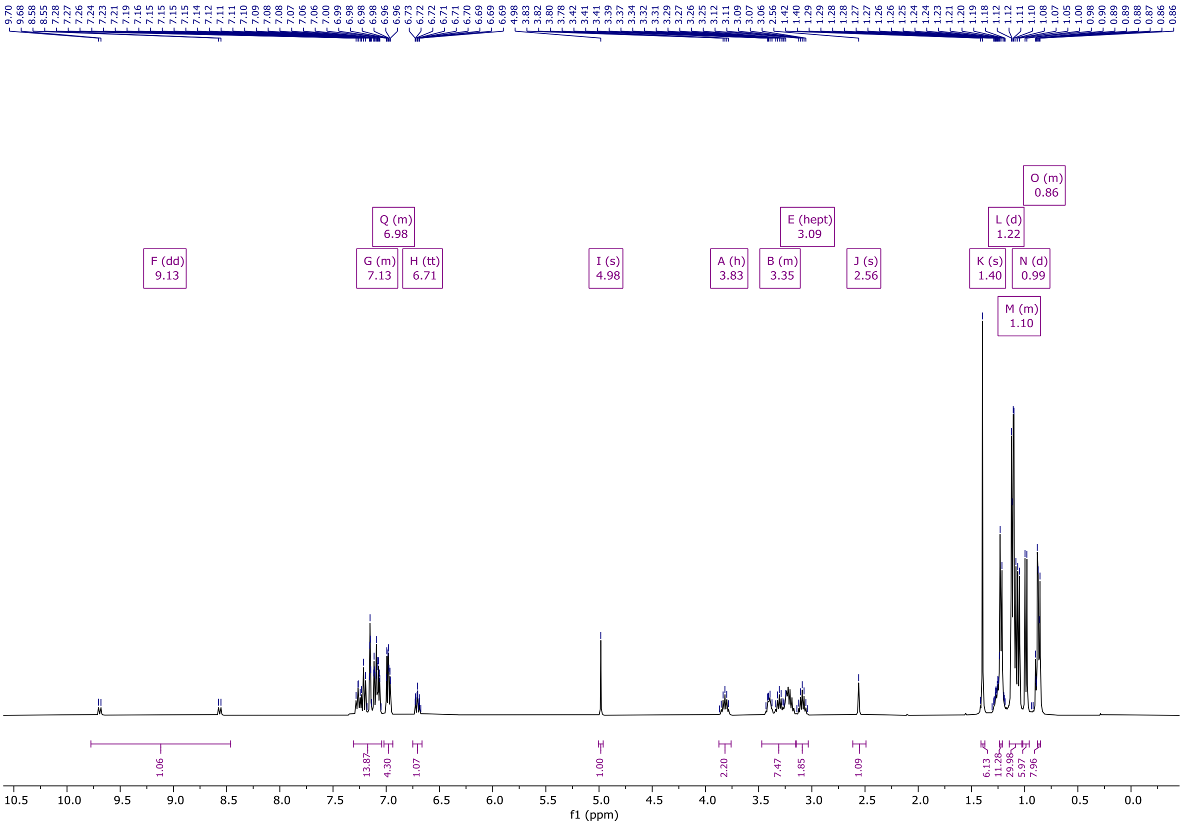

Figure S1. ^{1}H NMR spectrum (400 MHz, 293 K) of **1a** in C_6D_6.

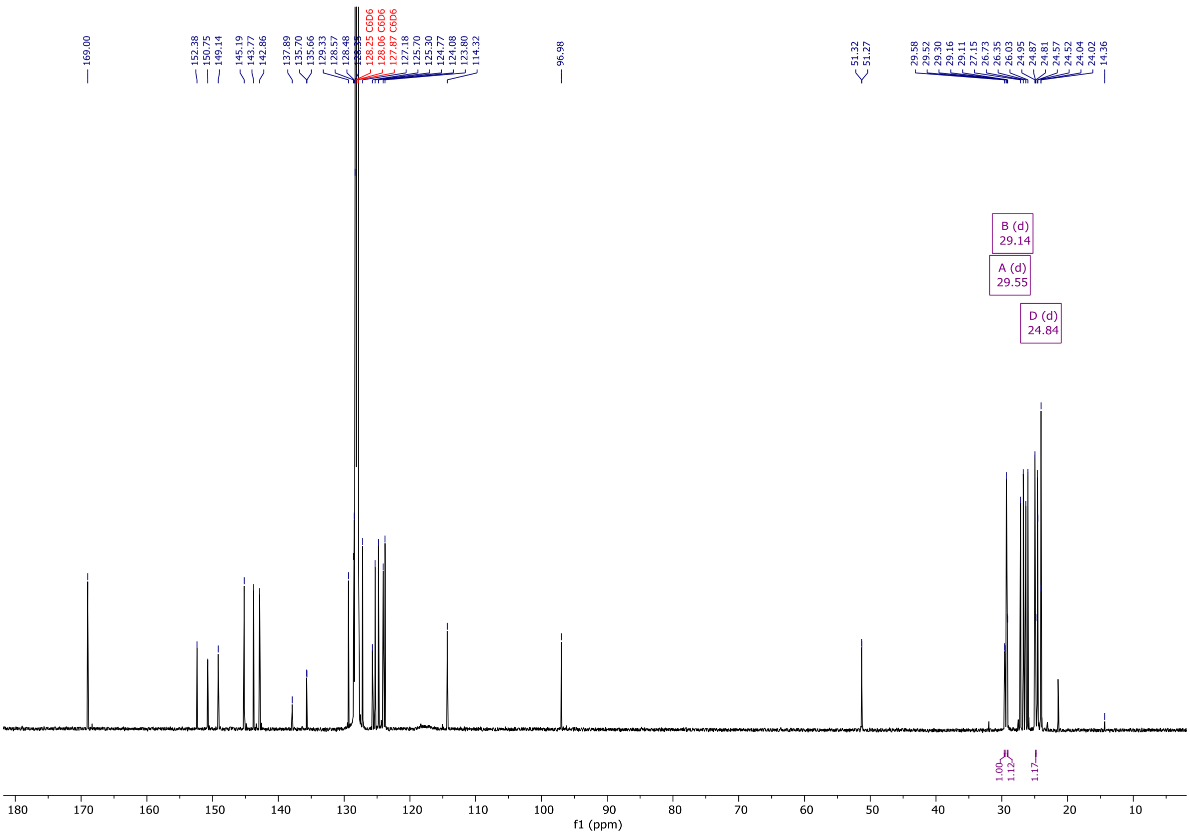

Figure S2. $^{13}C\{^{1}H\}$ NMR spectrum (126 MHz, 293 K) of **1a** in C_6D_6.

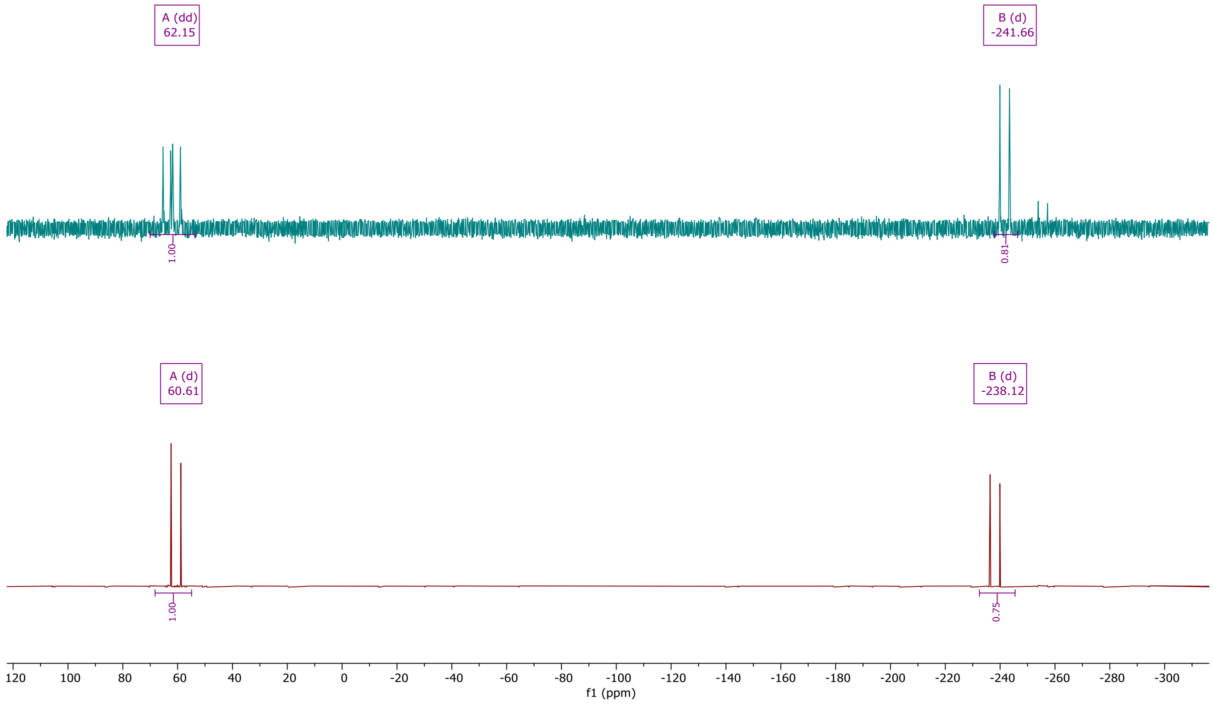

Figure S3. ^{31}P (top) and ^{31}P{^{1}H} (bottom) NMR spectra (162 MHz, 293 K) of **1a** in C_6D_6.

1.3. Synthesis of [CH$_2${N(Dipp)}]$_2$P(H)PGa(NH^{i}Pr)(NacNac) (1b)

A (50 mg, 0.054 mmol) was suspended in hexane (2 mL) and an excess of isopropylamine (0.01mL, 0.117 mmol) was added. The mixture immediately decolourised and a white precipitate was formed. The solids were filtered and collected to afford **1b** with >95% purity by ^{31}P NMR spectroscopy (0.028 g, 52.6%). **^{1}H NMR (400 MHz, C_6D_6):** δ (ppm) 9.42 (d, $^{1}J_{P–H}$ = 456.4 Hz, 1H; P*H*), 7.50–7.14 (m, 14H; ArC<u>*H*</u>), 4.96 (s, 1H; NacNac γ-H), 4.12–3.74 (m, 4H; Dipp {C*H*(CH$_3$)$_2$}), 3.70–3.29 (m, 8H; {C*H*(CH$_3$)$_2$} and {(NC*H*$_2$)$_2$}), 1.61 (s, 6H; NacNac C*H*$_3$), 1.58–1.27 (m, 30H; {C*H*(CH$_3$)$_2$}), 1.13–1.05 (m, 9H; Dipp {CH(C*H*$_3$)$_2$}), 0.91 (s, 6H; Dipp {CH(C*H*$_3$)$_2$}), 0.00 (s, 1H; N*H*). **^{13}C{^{1}H} NMR (126 MHz, C_6D_6):** δ (ppm) 168.48 (NacNac C), 150.62 (Ar*C*), 148.64 (Ar*C*), 143.28 (Ar*C*), 126.26 (Ar*C*), 125.33 (Ar*C*), 124.52 (Ar*C*), 123.95 (Ar*C*), 123.65 (Ar*C*), 97.35 (NacNac *C*H), 51.43 ({(N*C*H$_2$)$_2$}), 31.60 (Dipp

{*C*H(CH_3)$_2$}), 28.67 (Dipp {*C*H(CH_3)$_2$}), 28.61 (NacNac CH_3), 28.90 (Dipp {*C*H(CH_3)$_2$}), 28.46 (Dipp {*C*H(CH_3)$_2$}), 26.57 (Dipp {*C*H(CH_3)$_2$}), 26.11 (Dipp {CH(*C*H_3)$_2$}), 25.92 (Dipp {CH(*C*H_3)$_2$}), 25.54 (Dipp {CH(*C*H_3)$_2$}), 24.99 (Dipp {CH(*C*H_3)$_2$}), 24.52 (Dipp {CH(*C*H_3)$_2$}), 24.15 (Dipp {CH(*C*H_3)$_2$}), 24.00 (Dipp {CH(*C*H_3)$_2$}), 22.68 (Dipp {CH(*C*H_3)$_2$}), 13.98 (Dipp {CH(*C*H_3)$_2$}). **^{31}P NMR (162 MHz, C_6D_6):** δ (ppm) 62.15 (dd, $^1J_{P\text{–}P}$ = 573.8 Hz, $^1J_{P\text{–}H}$ = 456.2 Hz), –241.66 (d, $^1J_{P\text{–}P}$ = 573.6 Hz).

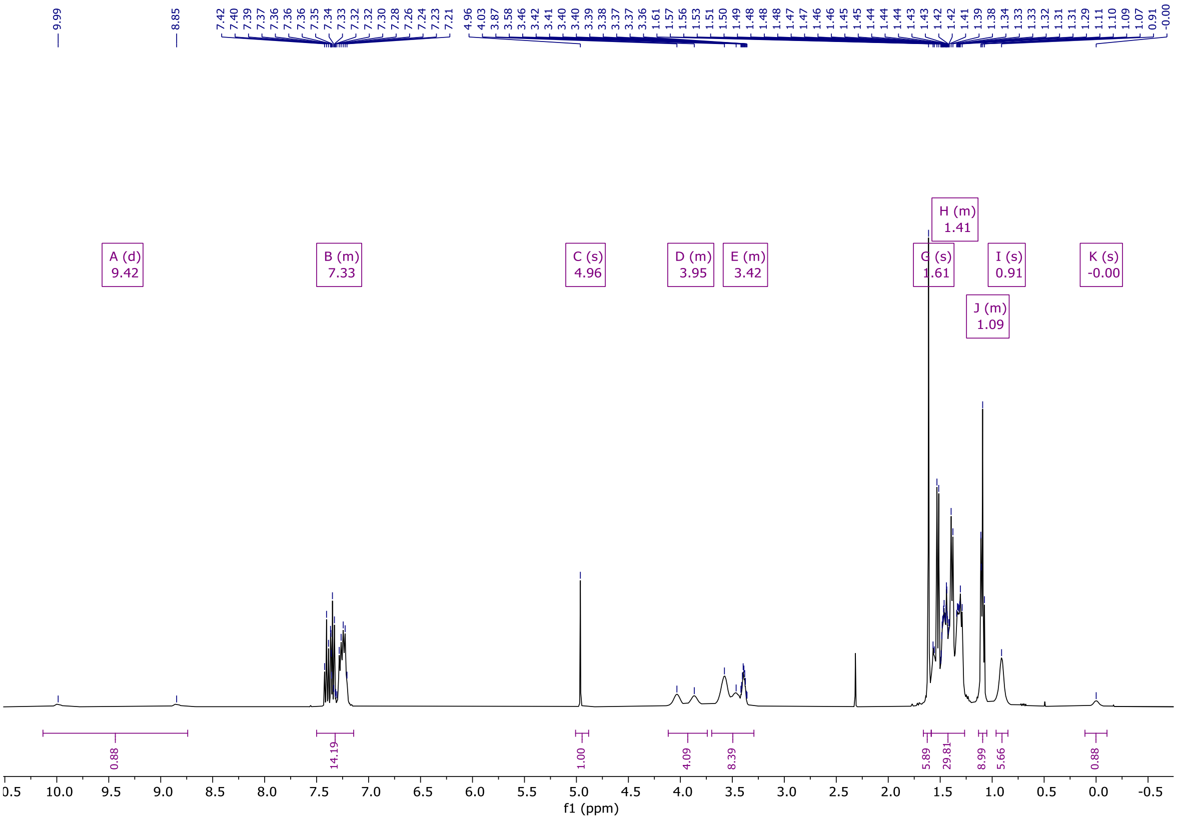

Figure S4. ^{1}H NMR spectrum (400 MHz, 293 K) of **1b** in C_6D_6.

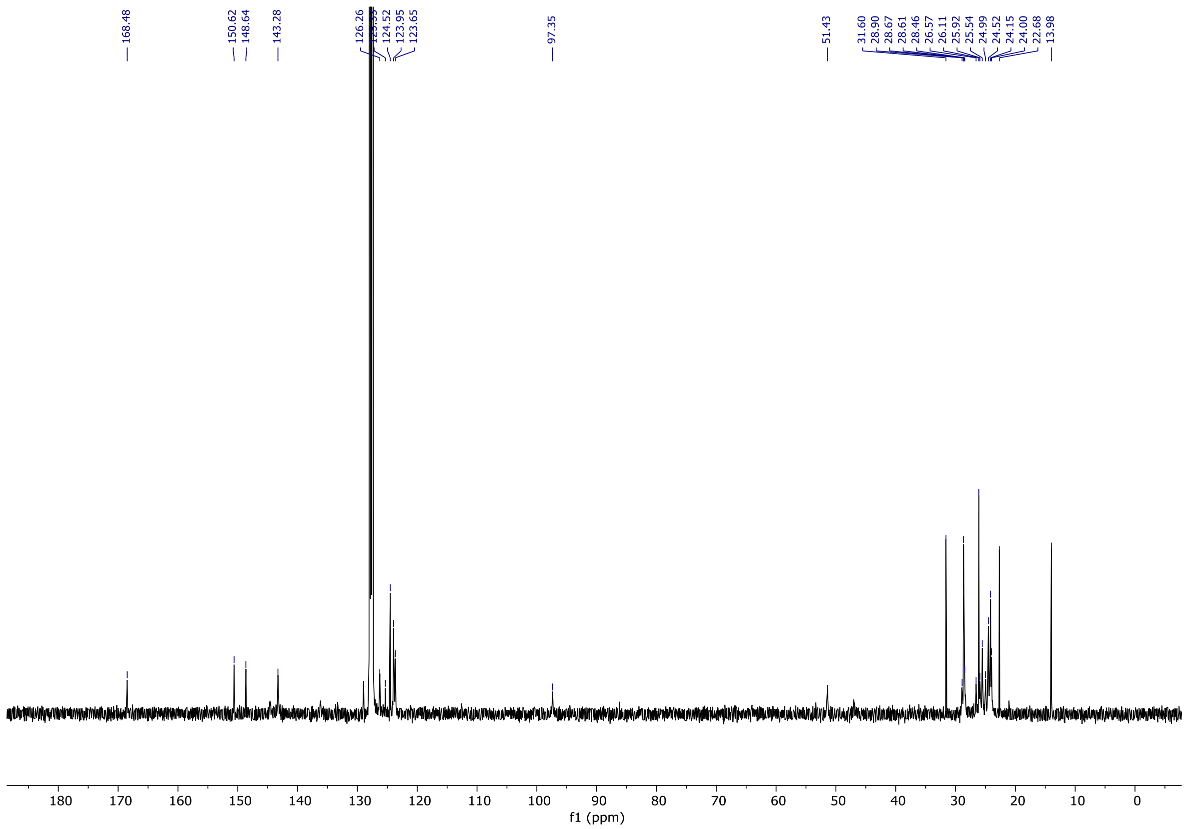

Figure S5. $^{13}C\{^{1}H\}$ NMR spectrum (126 MHz, 293 K) of **1b** in C_6D_6.

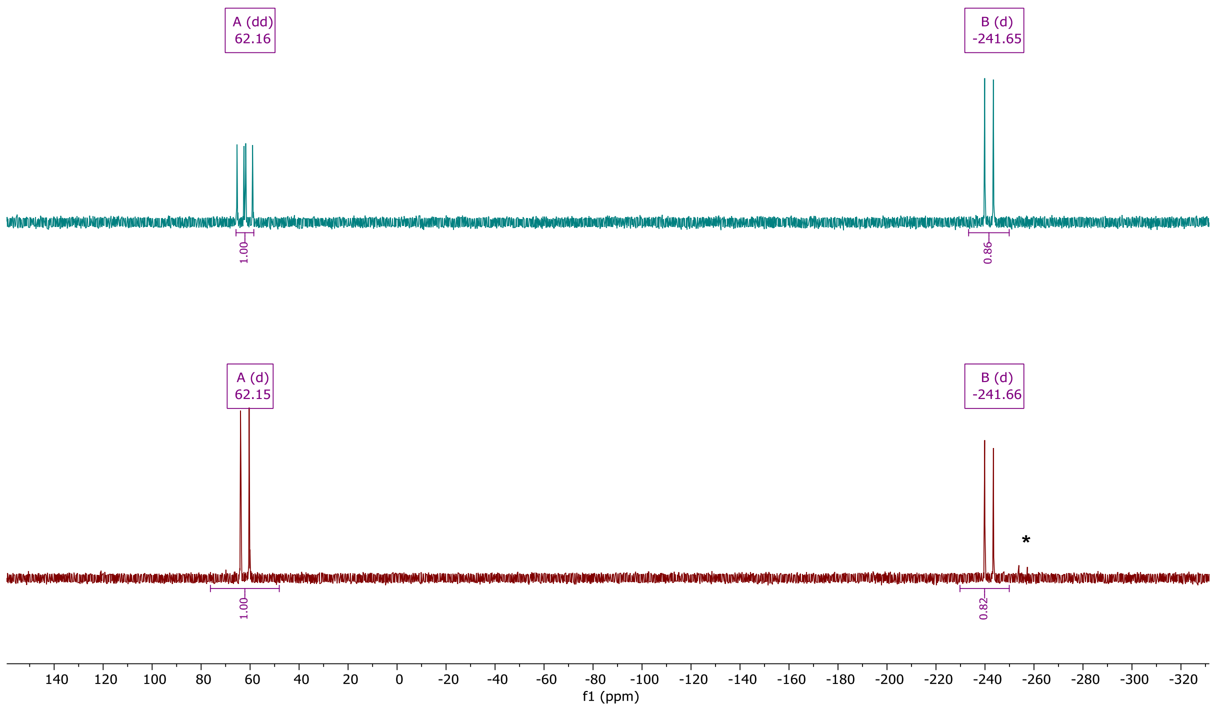

Figure S6. ^{31}P (top) and $^{31}P\{^{1}H\}$ (bottom) NMR spectra (162 MHz, 293 K) of **1b** in C_6D_6. * indicates presence of **2** due to hydrolysis.

1.4. [CH_2{N(Dipp)}]$_2$P(H)PGa(NH_2)(NacNac) (1c)

A (100 mg, 0.11 mmol) was suspended in hexane (2 mL) and put under 1 bar of NH_3. Upon shaking, the solution immediately decolourised and white precipitate was formed. The solids were filtered and collected to yield **1a** with >95% purity by ^{31}P NMR integration. (0.074 g, 72.7% assuming 100% conversion). **^{1}H NMR (400 MHz, C_6D_6):** δ (ppm) 9.08 (d, $^{1}J_{P–H}$ = 469.5 Hz, 1H; P*H*), 7.30–6.90 (m, 12H; ArC*H*), 4.65 (s, 1H; NacNac γ-H), 3.83 (m, 2H; {(NCH_2)$_2$}), 3.40–3.16 (m, 10H; {C*H*(CH_3)$_2$} and {(NCH_2)$_2$}), 1.42 (s, 6H; NacNac C*H*$_3$), 1.23 (d, $^{3}J_{H–H}$ = 6.8 Hz, 12H; ; Dipp {CH(C*H*$_3$)$_2$}), 1.16 (m, 12H; ; Dipp {CH(C*H*$_3$)$_2$}), 1.11 (m, 12H; Dipp {CH(C*H*$_3$)$_2$}), 1.05 (d, $^{3}J_{H–H}$ = 6.8 Hz, 6H; Dipp {CH(C*H*$_3$)$_2$}), 0.94 (d, $^{1}J_{H–H}$ = 6.7 Hz, 6H; Dipp {CH(C*H*$_3$)$_2$}), −0.38 (s, 2H; NH_2). **^{13}C{^{1}H} NMR (126 MHz, C_6D_6):** δ (ppm) 167.38 (NacNac *C*), 150.23 (Ar*C*), 149.09 (Ar*C*), 144.14 (Ar*C*), 143.16 (Ar*C*), 142.64 (Ar*C*), 136.28 (Ar*C*), 126.38 (Ar*C*), 124.48 (Ar*C*), 123.89 (Ar*C*), 123.40 (Ar*C*), 123.35 (Ar*C*), 95.22 (NacNac *C*H), 51.26 ({(N*C*H$_2$)$_2$}), 51.21 ({(N*C*H$_2$)$_2$}), 29.41 (d, $^{4}J_{P–C}$ = 5.6 Hz; (Dipp {*C*H(CH_3)$_2$})), 29.29 (Dipp {*C*H(CH_3)$_2$}), 29.14 (d, $^{4}J_{P–C}$ = 6.1 Hz; (Dipp {*C*H(CH_3)$_2$})), 27.71 (Dipp {*C*H(CH_3)$_2$}), 27.24 (Dipp {CH(*C*H$_3$)$_2$}), 26.55 (Dipp {CH(*C*H$_3$)$_2$}), 25.99 (Dipp {CH(*C*H$_3$)$_2$}), 25.01 (Dipp {CH(*C*H$_3$)$_2$}), 24.48 (Dipp {CH(*C*H$_3$)$_2$}), 24.42 (Dipp {CH(*C*H$_3$)$_2$}), 24.35 (Nacnac *C*H$_3$), 24.20 (Dipp {CH(*C*H$_3$)$_2$}), 24.04 (Dipp {CH(*C*H$_3$)$_2$}). **^{31}P NMR (162 MHz, C_6D_6):** δ (ppm) = 62.96 (dd, $^{1}J_{P–P}$ = 565.8 Hz, $^{1}J_{P–H}$ = 473.3 Hz), –252.06 (d, $^{1}J_{P–P}$ = 565.8 Hz).

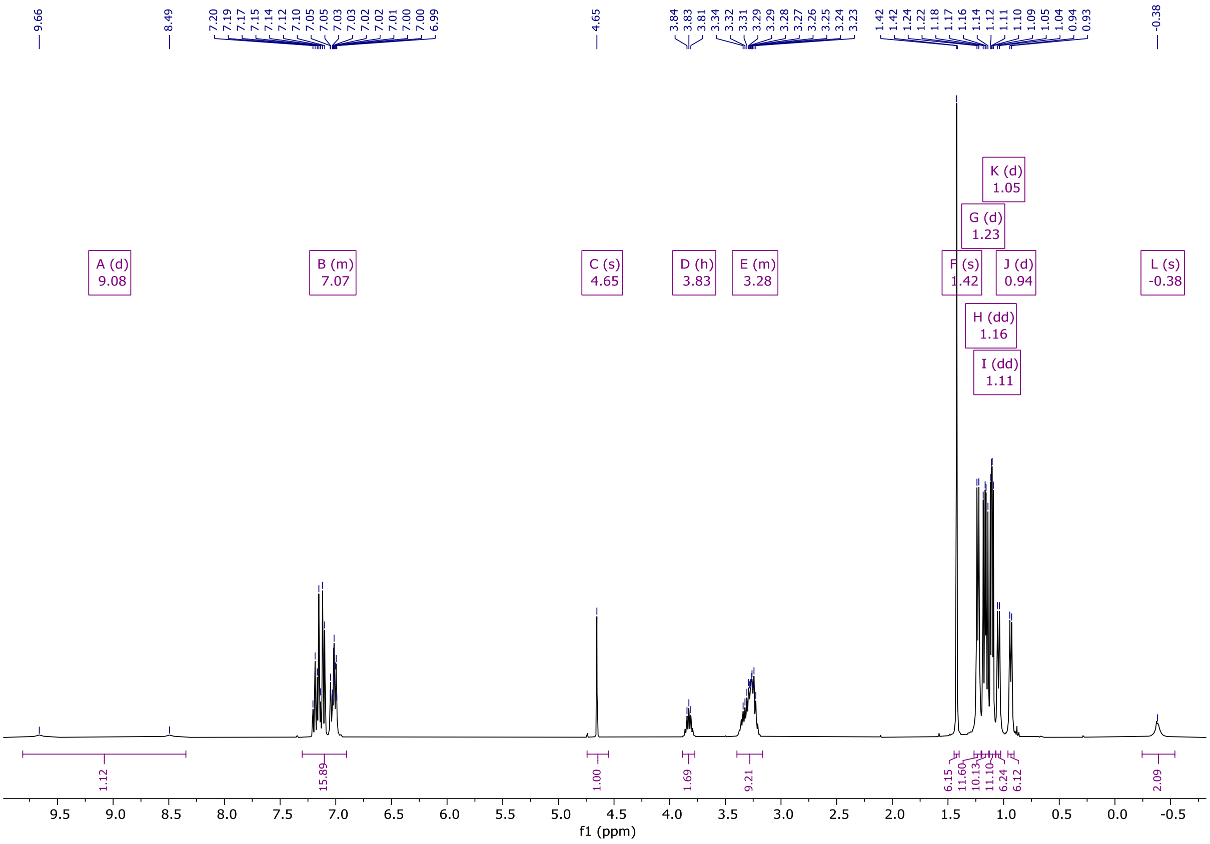

Figure S7. ^{1}H NMR spectrum (400 MHz, 293 K) of **1c** in C_6D_6.

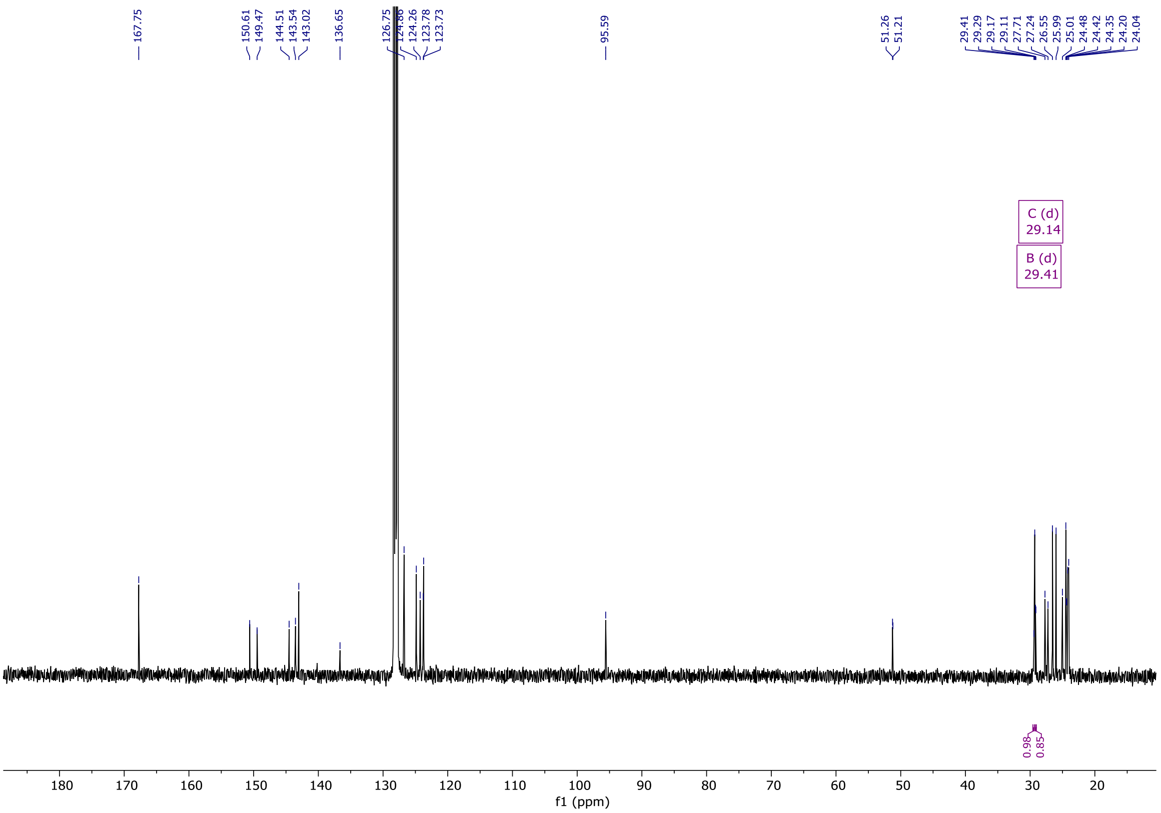

Figure S8. $^{13}C\{^{1}H\}$ NMR spectrum (126 MHz, 293 K) of **1c** in C_6D_6.

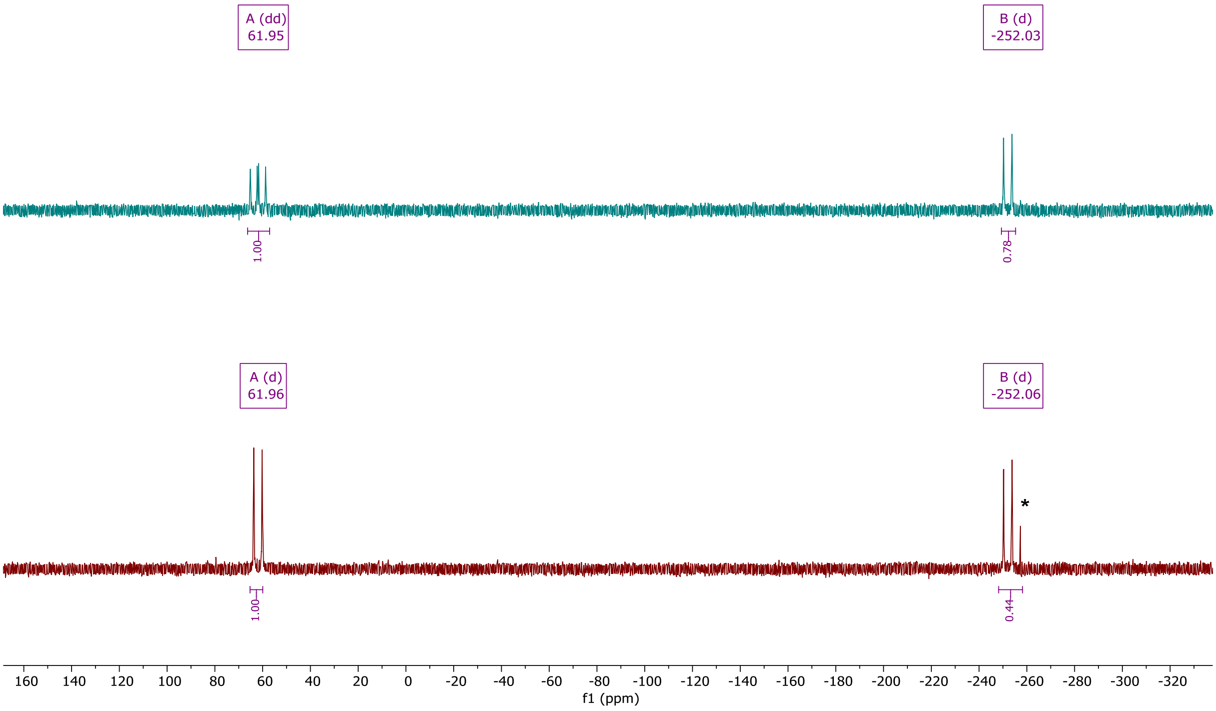

Figure S9. ^{31}P (top) and $^{31}P\{^{1}H\}$ (bottom) NMR spectra (162 MHz, 293 K) of **1c** in C_6D_6.

(* indicates presence of **2** due to slight hydrolysis).

1.5. [CH_2{N(Dipp)}]$_2$P(H)PGa(OH)(NacNac) (2)

A (50 mg, 0.054 mmol) was dissolved in toluene and a drop of water (0.01 mL, 0.556 mmol) was added. The mixture was stirred for half an hour and the solvent was removed under reduced pressure. Recrystallisation from hexane gave white crystalline solids. (0.032 g, 62.8%). Anal. calculated for $C_{55}H_{81}GaN_4O_1P_2$: C, 69.84; H, 8.63; N, 5.92. Found: C, 68.84; H, 8.64; N, 5.95. **^{1}H NMR (400 MHz, C_6D_6):** δ (ppm) 9.07 (dd, $^1J_{P–H}$ = 468.3, $^2J_{P–H}$ = 9.5 Hz, 1H; P*H*), 7.27–6.90 (m, 12H; ArC*H*), 4.74 (s, 1H; NacNac γ-H), 3.86–3.72 (sept, $^3J_{H–H}$ = 6.8 Hz, 2H; {C*H*(CH_3)$_2$}), 3.50 (sept, $^3J_{H–H}$ = 6.8 Hz, 2H; {C*H*(CH_3)$_2$}), 3.23 (m, 8H; {C*H*(CH_3)$_2$} and {(NCH_2}$_2$}), 1.42 (s, 6H; NacNac C*H*$_3$), 1.26–1.08 (m, 36H; Dipp {CH(C*H*$_3$)$_2$}), 1.03 (d, $^3J_{H–H}$ = 6.8 Hz, 6H; Dipp {CH(C*H*$_3$)$_2$}), 0.93 (d, $^3J_{H–H}$, 6H; Dipp {CH(C*H*$_3$)$_2$}), −0.38 (s, 1H; O*H*). **^{13}C{^{1}H} NMR (126 MHz, C_6D_6):** δ (ppm) 167.95 (NacNac C), 150.14 (Ar*C*), 149.14 (Ar*C*), 144.51 (Ar*C*), 142.98 (Ar*C*), 142.19 (ArC), 136.00 (d, $^2J_{P–C}$ = 5.6 Hz), 126.64 (Ar*C*), 124.42 (Ar*C*), 123.98 (Ar*C*), 123.55 (Ar*C*), 123.33 (Ar*C*), 95.83 (NacNac *C*H), 50.85 ({(N*C*H2)2}), 50.80 ({(N*C*H2)2}), 29.06 (d, $^4J_{P-C}$ = 5.8 Hz; Dipp {*C*H(CH_3)$_2$}), 28.97 (Dipp {*C*H(CH_3)$_2$}), 28.79 (d, 2$J_{P–C}$ = 6.2 Hz; {(N*C*H_2)$_2$}), 27.12 (Dipp {*C*H(CH_3)$_2$}), 26.35 (Dipp {CH(*C*H_3)$_2$}), 26.14 (Dipp {CH(*C*H_3)$_2$}), 25.50 (Dipp {CH(*C*H_3)$_2$}), 24.48 (Dipp {CH(*C*H_3)$_2$}), 24.31 (Dipp {CH(*C*H_3)$_2$}), 24.01 (d, $^4J_{P-C}$ = 7.2 Hz; (Dipp {*C*H(CH_3)$_2$})), 23.76 (NacNaC *C*H_3), 23.66 (d, $^4J_{P–C}$ = 6.9 Hz; (Dipp {*C*H(CH_3)$_2$})), 23.51 (Dipp {CH(*C*H_3)$_2$}). **^{31}P NMR (162 MHz, C_6D_6):** δ (ppm) 61.8 (dd, $^1J_{P–P}$ = 557.7 Hz, $^1J_{P–H}$ = 468.3 Hz), –255.6 (dd, $^1J_{P–H}$ = 557.7 Hz, $^3J_{P–H}$ = 9.5 Hz)

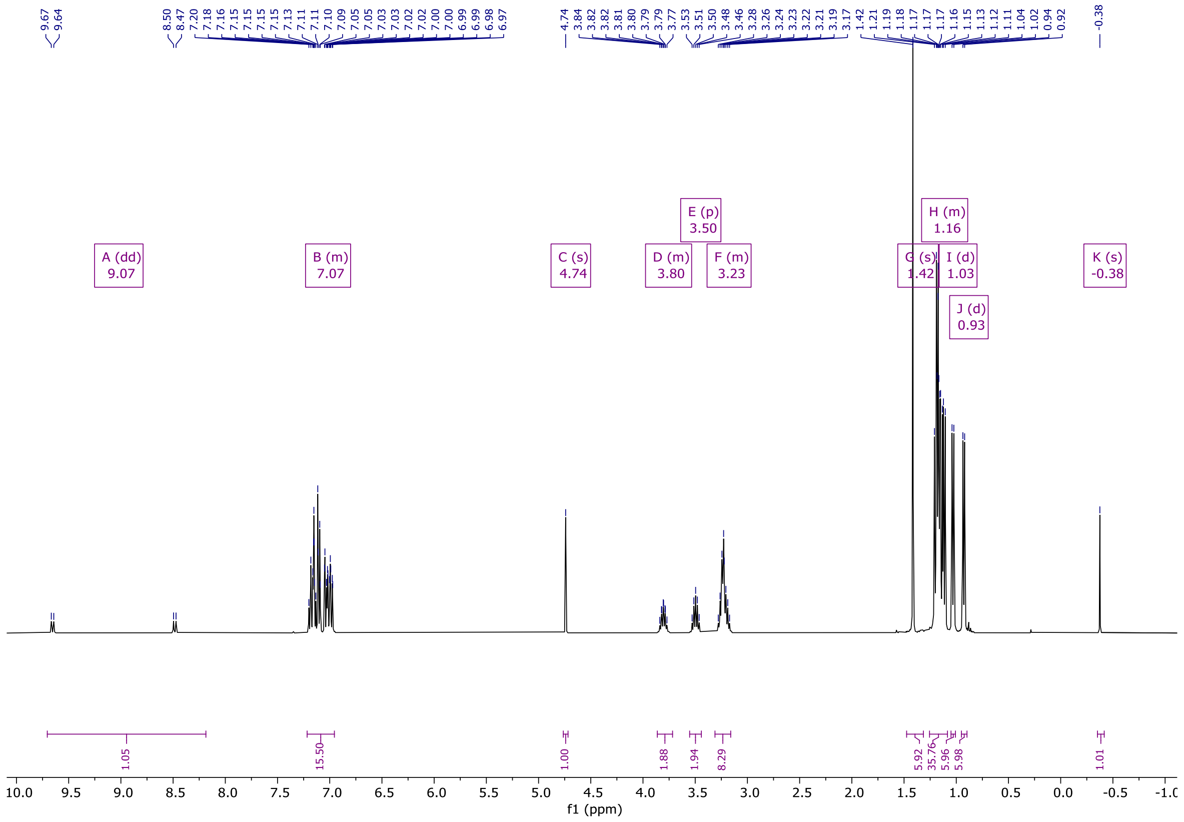

Figure S10. ^{1}H NMR spectrum (400 MHz, 293 K) of **2** in C_6D_6.

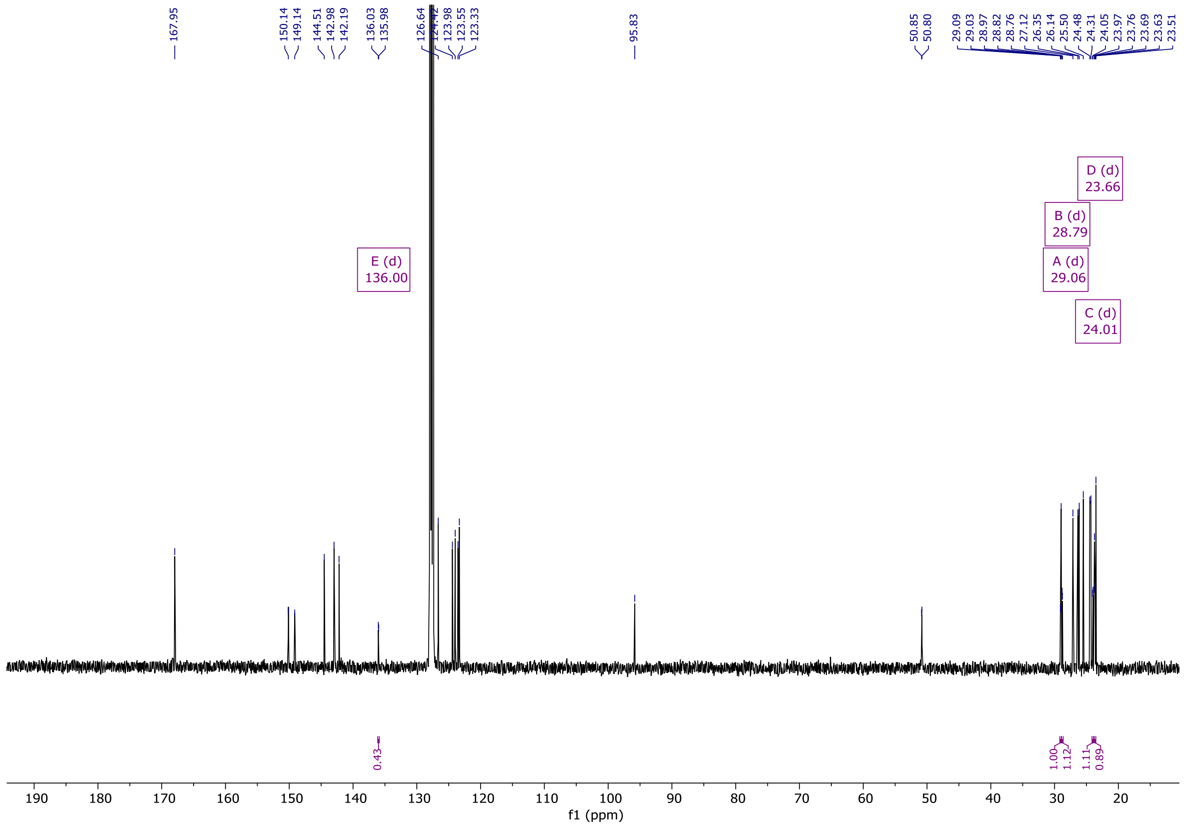

Figure S11. $^{13}C\{^{1}H\}$ NMR spectrum (126 MHz, 293 K) of **2** in C_6D_6.

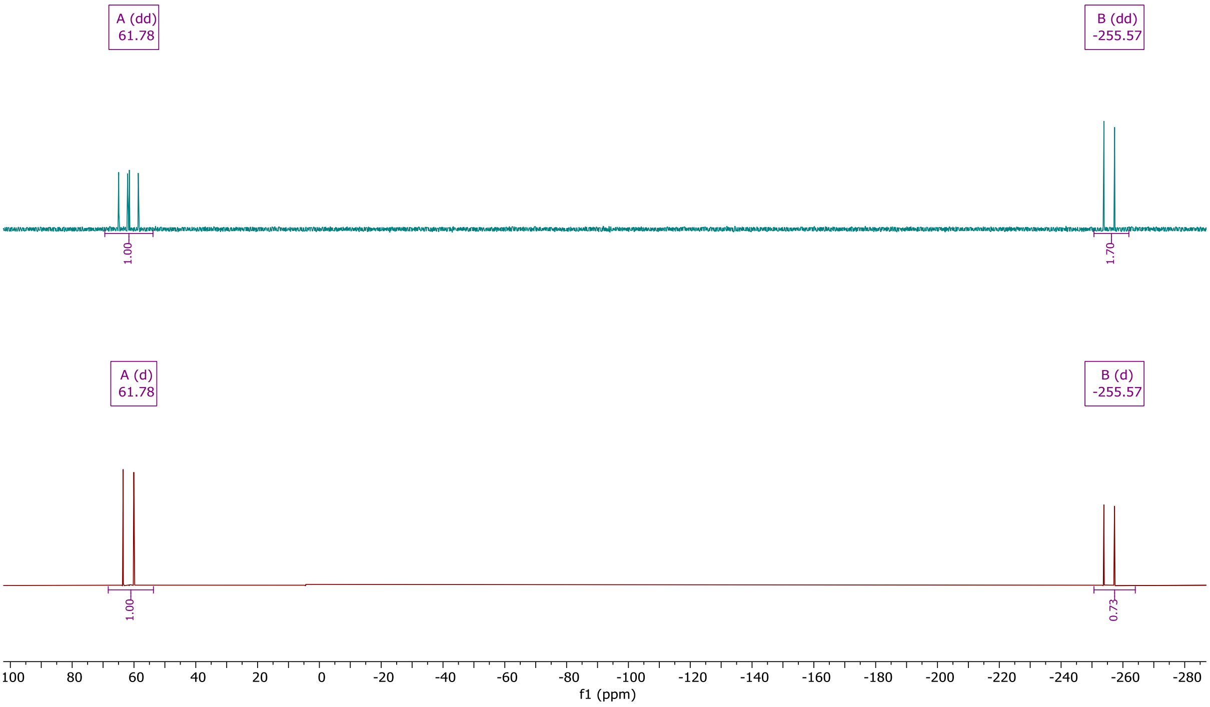

Figure S12. ^{31}P (top) and ^{31}P{^{1}H} (bottom) NMR spectra (162 MHz, 293 K) of **2** in C_6D_6.

1.6. Synthesis of [CH_2{N(Dipp)}]$_2$PP(H)Ga(NH^{i}Pr)(NacNac) (3b)

1b (50 mg, 0.051 mmol) was dissolved in toluene and heated at 80 °C for overnight. The solvent was removed and recrystallisation from hexane yielded colourless crystals of **3b** (Yield = 0.023 mg, 45.7%). Anal. calculated for $C_{58}H_{88}GaN_4P_2$: C, 70.58; H, 8.99; N, 7.10. Found: C, 70.18; H, 8.73; N, 5.97. **^{1}H NMR (400 MHz, C_6D_6):** δ (ppm) 7.31–6.88 (m, 15H; Ar*H*), 4.75 (s, 1H; NacNac C*H*), 3.82 (p, $^{3}J_{H–H}$ = 6.8 Hz, 2H; {C*H*(CH_3)$_2$}), 3.77–3.63 (m, 4H; {C*H*(CH_3)$_2$} and {(NCH_2)$_2$}), 3.59 (sept, $^{3}J_{H–H}$ = 6.8 Hz, 2H; {C*H*(CH_3)$_2$}), 3.42 (sept, $^{3}J_{H–H}$ = 6.2 Hz, 1H; NHC*H*(CH_3)$_2$)), 3.20 (sept, $^{3}J_{H–H}$ = 6.8 Hz, 2H; {C*H*(CH_3)$_2$}), 3.06 (m, 2H; {(NCH_2)$_2$}), 1.50 (d, $^{3}J_{H–H}$ = 6.8 Hz, 6H; {CH(CH_3)$_2$}), 1.37 (m, 12H; {CH(CH_3)$_2$} and NacNac CH_3), 1.28 (d, $^{3}J_{H–H}$ = 6.2 Hz, 6H; NHCH(CH_3)$_2$}), 1.24 (d, $^{3}J_{H–H}$ = 6.9 Hz, 6H; {CH(CH_3)$_2$}), 1.17 (d, $^{3}J_{H–H}$ = 6.9 Hz, 5H; {CH(CH_3)$_2$}), 1.14 (dd, $^{1}J_{P–H}$ = 171.2, $^{2}J_{P–H}$ = 5.7

Hz, 1H; PH), 1.11 (m, 12H; {CH(CH_3)$_2$}), 0.49 (m, 12H; {CH(CH_3)$_2$}). [Note: we were unable to observe the NH resonance, presumably due to overlap with other resonances.] **^{13}C{^{1}H} NMR (126 MHz, C_6D_6):** δ (ppm) 168.80 (NacNac C), 149.29 (Ar*C*), 147.79 (Ar*C*), 145.35 (Ar*C*), 143.16 (Ar*C*), 142.56 (Ar*C*), 138.57 (d, $^2J_{P–C}$ = 12.4 Hz; ArC) , 126.79 (Ar*C*), 126.70 (Ar*C*), 124.40 (Ar*C*), 124.28 (Ar*C*), 124.04 (Ar*C*), 123.97 (Ar*C*), 97.64 (NacNac *C*H), 55.28 ({(N*C*H2)2}), 55.20 ({(N*C*H2)2}), 47.23 (NH*C*H(CH$_3$)$_2$), 28.56 (Dipp {*C*H(CH$_3$)$_2$}), 28.46 (Dipp {*C*H(CH$_3$)$_2$}), 28.36 (Dipp {CH(*C*H$_3$)$_2$}), 28.27 (d, $^4J_{P–C}$ = 4.3 Hz; Dipp {*C*H(CH$_3$)$_2$}), 26.40 (Dipp {*C*H(CH$_3$)$_2$}), 25.77 (Dipp {CH(*C*H$_3$)$_2$}), 25.63 (Dipp {CH(*C*H$_3$)$_2$}), 25.35 (d, $^5J_{P–C}$ = 2.9 Hz; Dipp {CH(*C*H$_3$)$_2$}), 25.28 (d, $^5J_{P–C}$ = 2.1 Hz; Dipp {CH(*C*H$_3$)$_2$}), 24.50 (d, $^5J_{P–C}$ = 2.5 Hz; Dipp {CH(*C*H$_3$)$_2$}), 24.32 (Dipp {CH(*C*H$_3$)$_2$}), 24.01 (Dipp {CH(*C*H$_3$)$_2$}), 23.83 (Dipp {CH(*C*H$_3$)$_2$}), 23.79 (NacNac *C*H$_3$). **^{31}P NMR (162 MHz, C_6D_6):** δ (ppm) = 150.59 (dd, $^1J_{P–P}$ = 239.2 Hz), –191.04 (d, $^1J_{P–P}$ = 239.5 Hz, $^1J_{P–H}$ = 171.3 Hz).

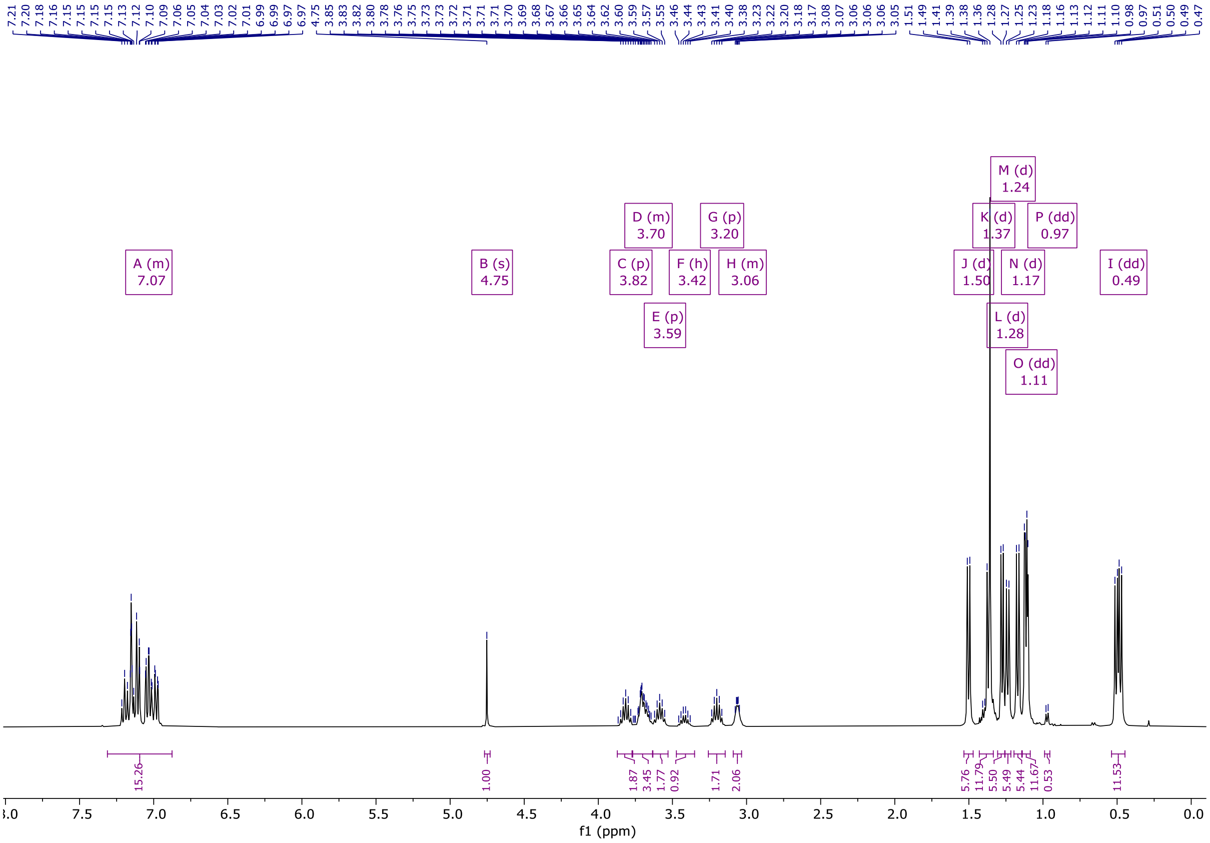

Figure S13. ^{1}H NMR spectrum (400 MHz, 293 K) of **3b** in C_6D_6.

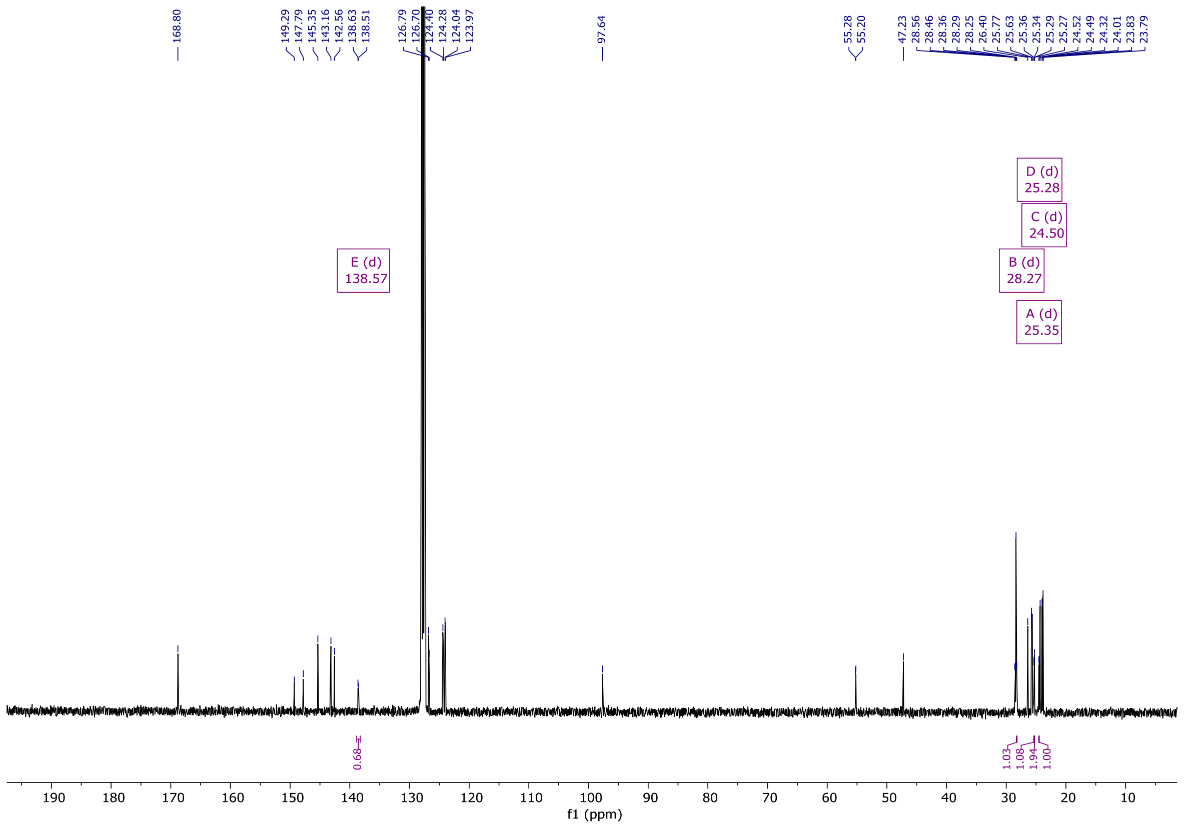

Figure S14. $^{13}C\{^{1}H\}$ NMR spectrum (126 MHz, 293 K) of **3b** in C_6D_6.

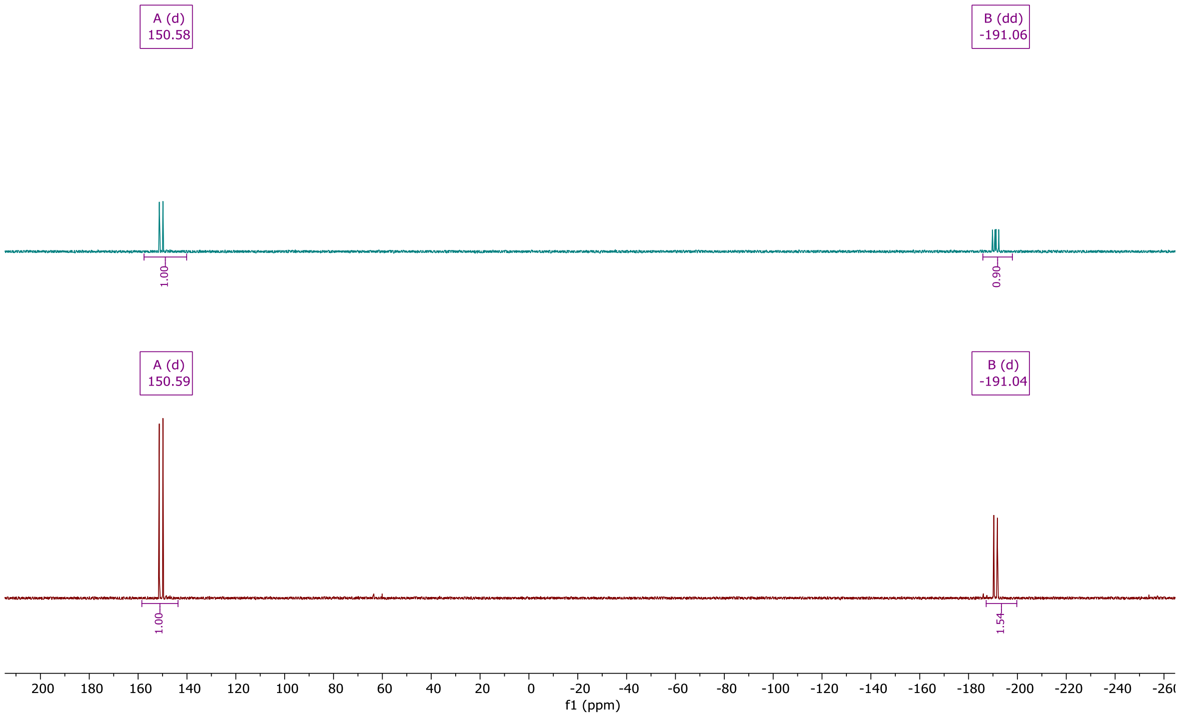

Figure S15. ^{31}P (top) and $^{31}P\{^{1}H\}$ (bottom) NMR spectra (162 MHz, 293 K) of **3b** in C_6D_6.

1.7. Synthesis of $[CH_2\{N(Dipp)\}]_2PP(H)Ga(NH_2)(NacNac)$ (3c)

1a (50 mg, 0.053 mmol) was dissolved in toluene and heated at 110°C for 2 days. The solvent was removed and recrystallisation from hexane yielded colourless crystals (0.033 g, 66.0%).

^{1}H NMR (400 MHz, C_6D_6): δ (ppm) 7.23 – 6.92 (m, 12H; ArC*H*), 4.71 (s, 1H; NacNac γ-H), 3.78 – 3.54 (m, 8H; {(NCH_2}$_2$} and Dipp {C*H*(CH_3)$_2$}), 3.26 (h, $^3J_{H–H}$ = 6.9 Hz, 2H; Dipp {CH(CH_3)$_2$}), 3.13 – 3.08 (m, 2H; {(NCH_2}$_2$}), 1.50 – 1.36 (m, 18H; Dipp {CH(CH_3)$_2$} and NacNac CH_3), 1.22 (d, $^3J_{H–H}$ = 6.9 Hz, 6H; Dipp {CH(CH_3)$_2$}), 1.18 – 1.04 (m, 17H; Dipp {CH(CH_3)$_2$}), 1.11 (dd, $^1J_{P–H}$ = 169.1 Hz, $^2J_{P–H}$ = 7.0 Hz, 1H; P*H*), 0.67 (m, 12H; Dipp {CH(CH_3)$_2$}), 0.47 (s, 2H; NH_2). **^{13}C{^{1}H} NMR (126 MHz, C_6D_6):** δ (ppm) 167.94 (NacNac *C*), 149.23 (Ar*C*), 147.83 (Ar*C*), 145.03 (Ar*C*), 142.50 (Ar*C*), 142.12 (Ar*C*), 138.44 (Ar*C*), 138.32 (Ar*C*), 126.69 (Ar*C*), 126.58 (Ar*C*), 124.23 (Ar*C*), 124.18 (Ar*C*), 123.85 (Ar*C*), 123.76 (Ar*C*), 96.05 (NacNac *C*H), 55.23 ({(N*C*H_2}$_2$}), 55.15 ({(N*C*H_2}$_2$}), 28.56 (Dipp {*C*H(CH_3)$_2$), 28.54 (d, $^4J_{P–H}$ = 10.6 Hz; Dipp {*C*H(CH_3)$_2$), 28.29 (d, $^4J_{P–H}$ = 4.9 Hz; Dipp {*C*H(CH_3)$_2$), 27.32 (Dipp {*C*H(CH_3)$_2$), 26.19 (d, $^5J_{P–H}$ = 3.5 Hz; Dipp {*C*H(CH_3)$_2$), 26.02 (Dipp {*C*H(CH_3)$_2$), 25.73 (Dipp {*C*H(CH_3)$_2$), 24.70 (d, $^4J_{P–H}$ = 3.1 Hz; Dipp {*C*H(CH_3)$_2$), 24.47 (Dipp {*C*H(CH_3)$_2$), 24.42 (d, $^4J_{P–H}$ = 3.2 Hz; Dipp {*C*H(CH_3)$_2$), 23.77 (Dipp {*C*H(CH_3)$_2$), 23.68 (Dipp {*C*H(CH_3)$_2$), 23.32 (NacNac *C*H_3). **^{31}P NMR** (162 MHz, C_6D_6): δ = 150.69 (d, $^1J_{P–P}$ = 234.1 Hz), –187.79 (dd, $^1J_{P–P}$ = 234.3 Hz, $^1J_{P–H}$ = 169.4 Hz)

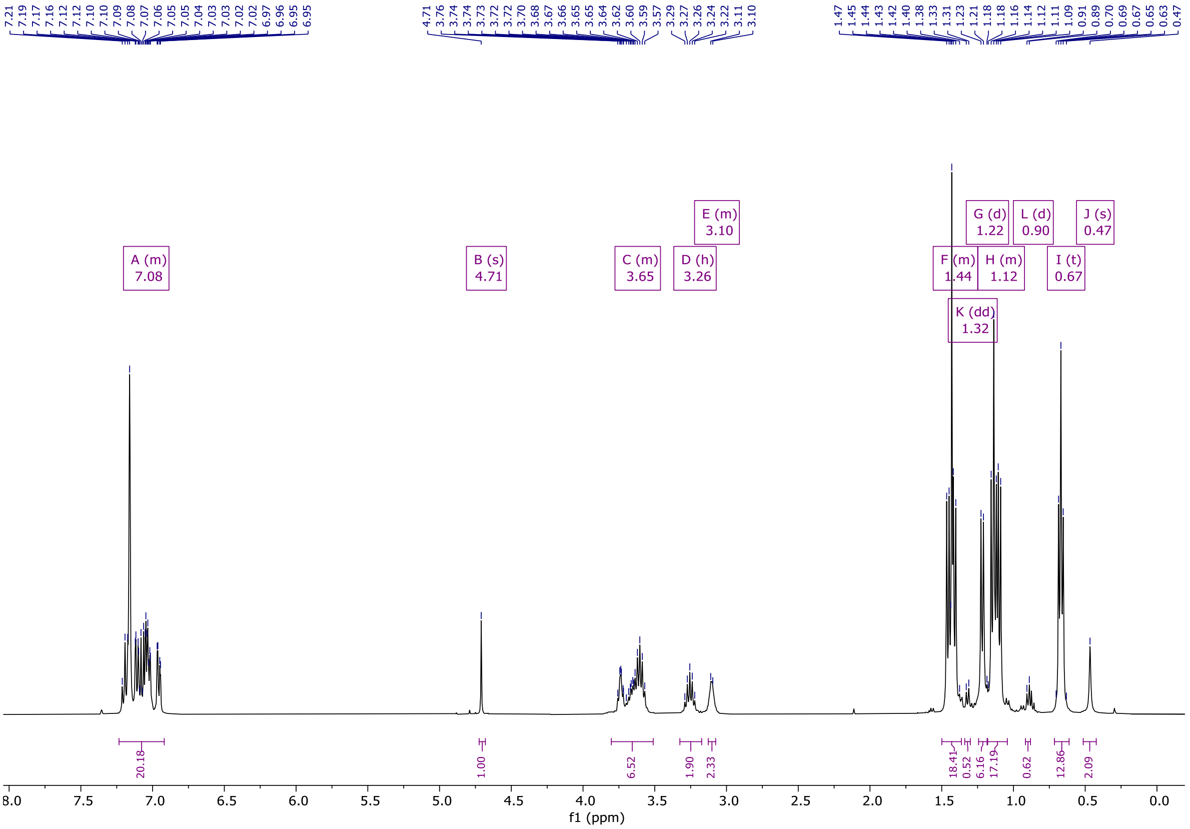

Figure S16. ^{1}H NMR spectrum (400 MHz, 293 K) of **3c** in C_6D_6.

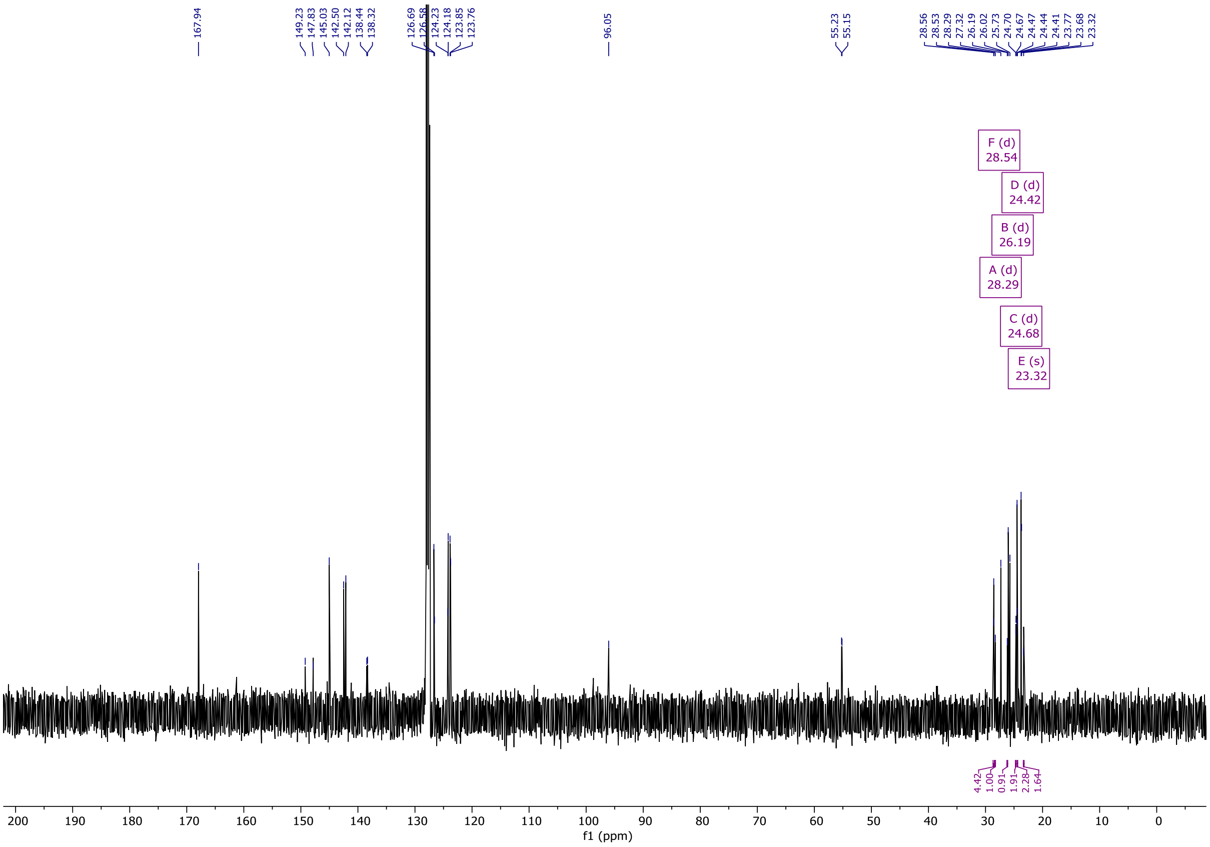

Figure S17. $^{13}C\{^{1}H\}$ NMR spectrum (126 MHz, 293 K) of **3c** in C_6D_6.

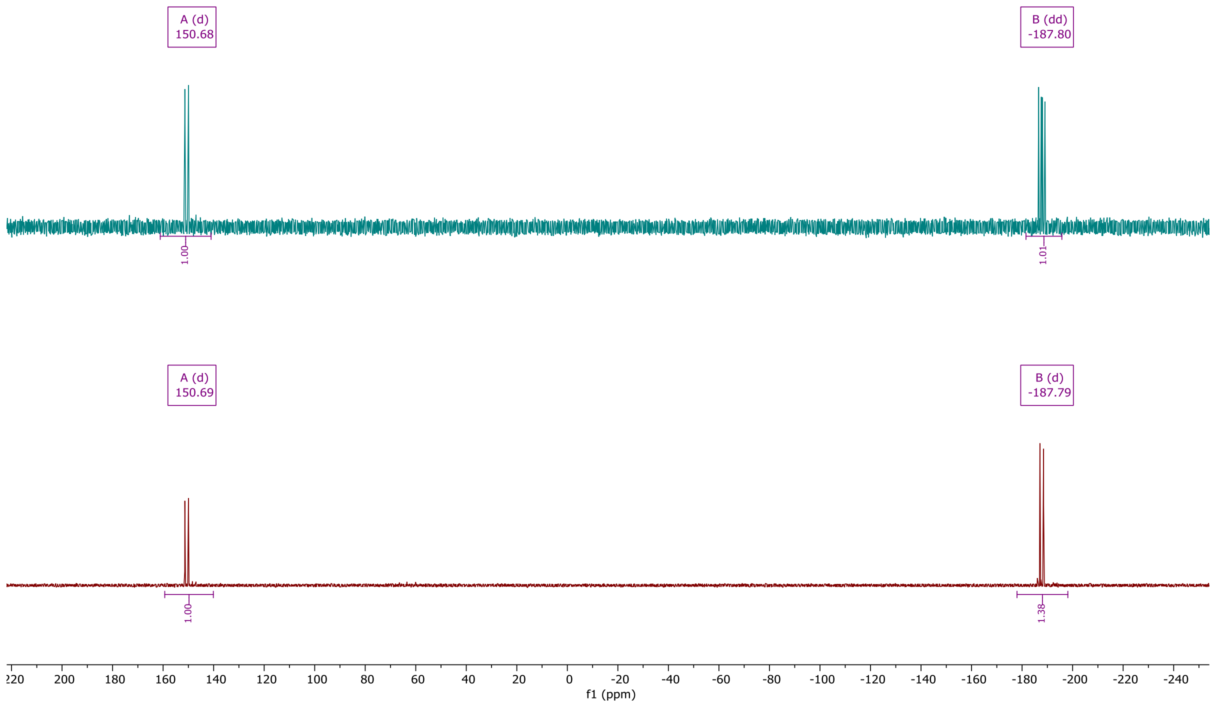

Figure S18. ^{31}P (top) and $^{31}P\{^1H\}$ (bottom) NMR spectra (162 MHz, 293 K) of **3c** in C_6D_6.

1.9. Synthesis of [CH2{N(Dipp)}]2P(H)PGa(CCPh)(NacNac) (4)

A (50 mg, 0.054 mmol) was dissolved in toluene and a drop of phenylacetylene was added. The mixture immediately decolourised and the solvent was removed under reduced pressure. Recrystallisation from hexane gave white crystalline solids. (0.030 g, 54.1%). Anal. calculated for $C_{63}H_{85}GaN_4P_2$: C, 73.46; H, 8.32; N, 5.44. Found: C, 73.22; H, 8.38; N, 5.35. **^{1}H NMR (400 MHz, C_6D_6):** δ (ppm) 9.28 (dd, $^1J_{P–H}$ = 470.0 Hz $^2J_{P–H}$ = 11.5 Hz, 1H; P*H*), 7.60–7.51 (m, 2H; ArC*H*), 7.29–6.91 (m, 15H; ArC*H*), 4.84 (s, 1H; NacNac γ-H), 3.84 (sept, $^3J_{H–H}$ = 6.7 Hz, 2H; {C*H*(CH$_3$)$_2$}), 3.66 (sept, $^3J_{H–H}$ = 6.7 Hz, 2H; {C*H*(CH$_3$)$_2$}), 3.44–3.32 (m, 6H; {C*H*(CH$_3$)$_2$} and {(NCH$_2$}$_2$}), 3.28–3.13 (m, 2H; {(NCH$_2$}$_2$}), 1.51–1.40 (m, 12H; Dipp {CH(C*H*$_3$)$_2$} and NacNac C*H*$_3$), 1.31–1.20 (m, 12H; Dipp {CH(C*H*$_3$)$_2$}), 1.18–1.06 (m, 18H;

Dipp {CH(C*H*$_3$)$_2$}), 0.88 (d, $^3J_{\text{H–H}}$ = 6.8 Hz, 6H; Dipp {CH(C*H*$_3$)$_2$}), 0.75 (d, $^3J_{\text{H–H}}$ = 6.7 Hz, 6H). **^{13}C{^{1}H} NMR (126 MHz, C_6D_6):** δ (ppm) 168.64 (NacNac *C*), 150.74 (Ar*C*), 149.05 (Ar*C*), 145.39 (Ar*C*), 143.06 (Ar*C*), 142.86 (Ar*C*), 136.18 (d, $^2J_{\text{P–H}}$ = 5.0 Hz; ArC), 126.98 (Ar*C*), 126.59 (Ar*C*), 124.63 (Ar*C*), 124.23 (Ar*C*), 124.23 (Ar*C*), 124.19 (Ar*C*), 124.03 (Ar*C*), 105.39 (PhC≡CGa), 97.45 (NacNac *C*H), 51.94 ({(N*C*H$_2$}$_2$}), 51.88 ({(N*C*H$_2$}$_2$}), 29.16 (d, $^4J_{\text{P–C}}$ = 3.3 Hz; Dipp {*C*H(CH$_3$)$_2$}), 29.06 (Dipp {*C*H(CH$_3$)$_2$}), 29.05 (d, $^4J_{\text{P–C}}$ = 5.4 Hz; Dipp {*C*H(CH$_3$)$_2$}), 27.78 (Dipp {CH(CH$_3$)$_2$}), 27.60 (Dipp {CH(*C*H$_3$)$_2$}), 26.50 (Dipp {CH(*C*H$_3$)$_2$}), 25.83 (Dipp {CH(*C*H$_3$)$_2$}), 25.01 (Dipp {CH(*C*H$_3$)$_2$}), 24.74 (d, $^5J_{\text{P–H}}$ = 5.1 Hz; Dipp {CH(*C*H$_3$)$_2$}), 24.64 (Dipp {CH(*C*H$_3$)$_2$}), 24.57 (d, $^5J_{\text{P–H}}$ = 5.8 Hz; Dipp {CH(*C*H$_3$)$_2$}), 24.45 (Dipp {CH(*C*H$_3$)$_2$}), 24.18 (NacNac *C*H$_3$). **^{31}P NMR (162 MHz, C_6D_6):** δ (ppm) 70.90 (ddt, $^1J_{\text{P–P}}$ = 567.3 Hz, $^1J_{\text{P–H}}$ = 471.2 Hz, $^3J_{\text{P–H}}$ = 6.6 Hz), –236.49 (dd, *J* = 568.1 Hz, $^2J_{\text{P–H}}$ = 11.5 Hz).

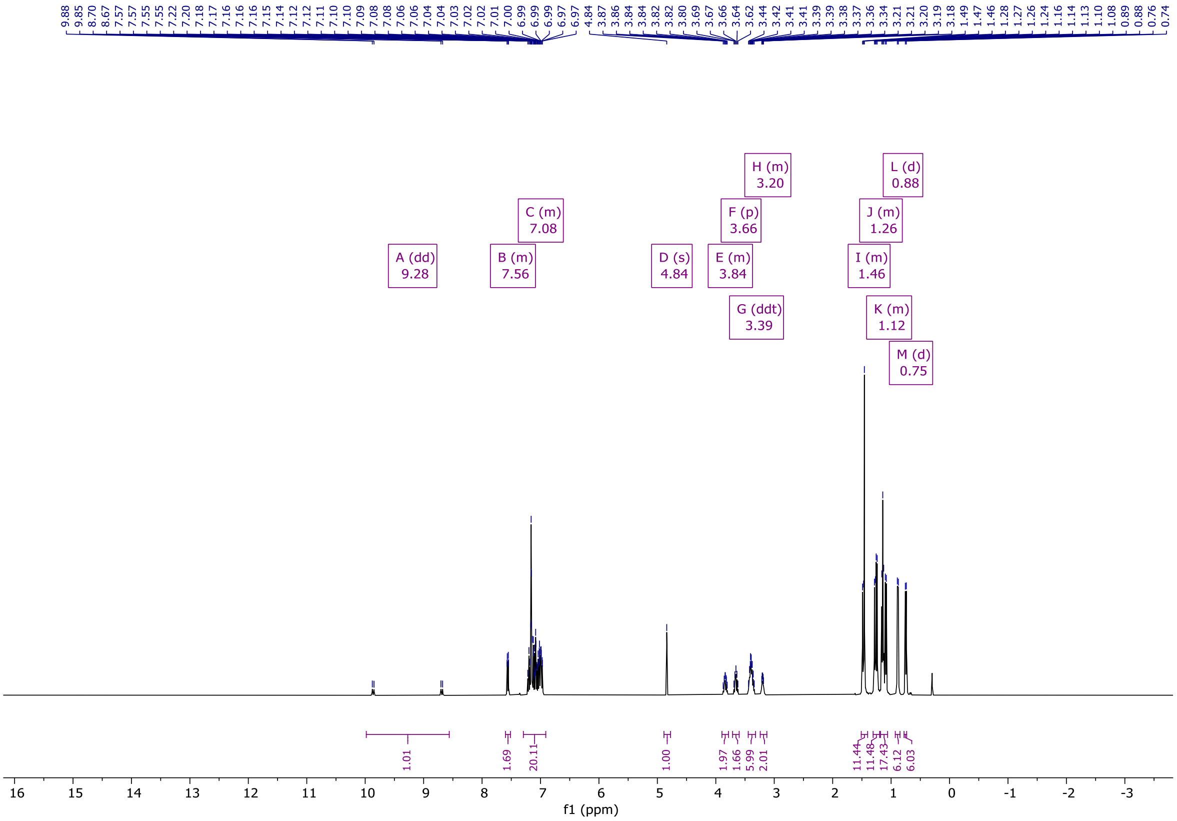

Figure S19. ^{1}H NMR spectrum (400 MHz, 293 K) of **4** in C_6D_6.

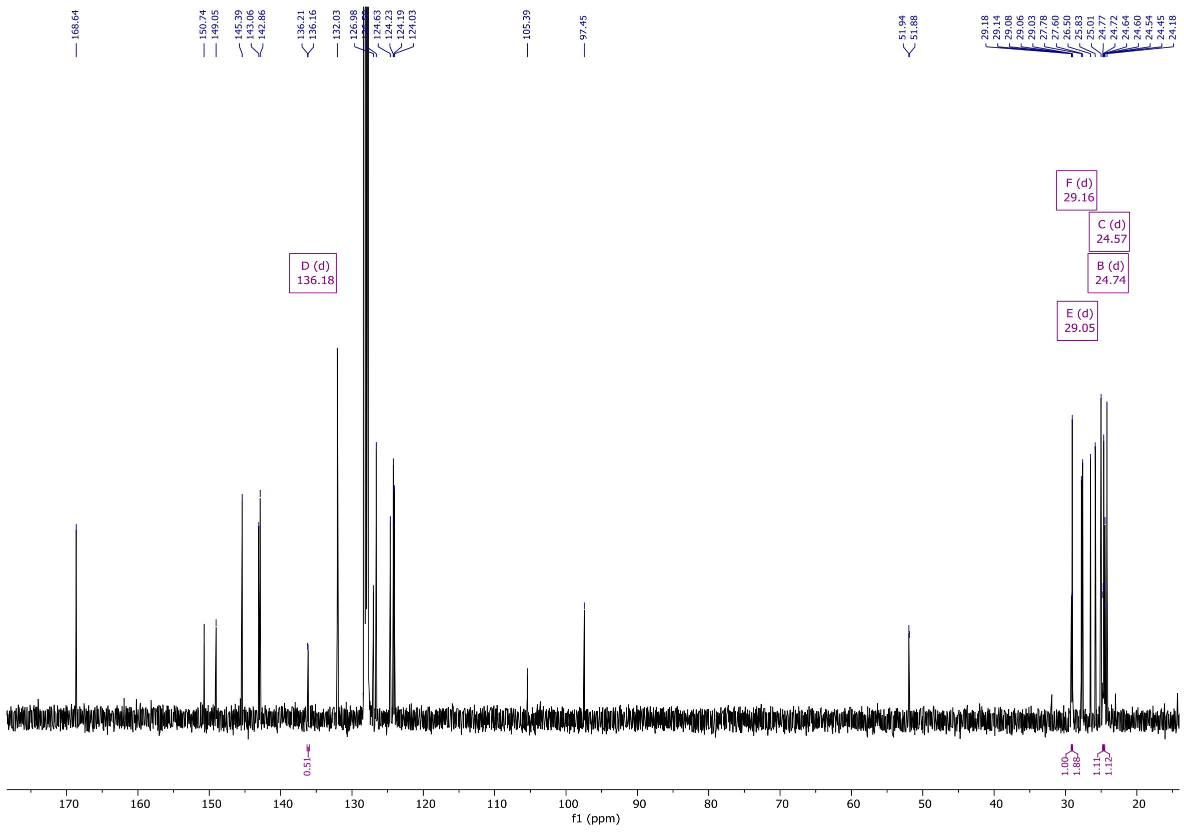

Figure S20. $^{13}C\{^{1}H\}$ NMR spectrum (126 MHz, 293 K) of **4** in C_6D_6.

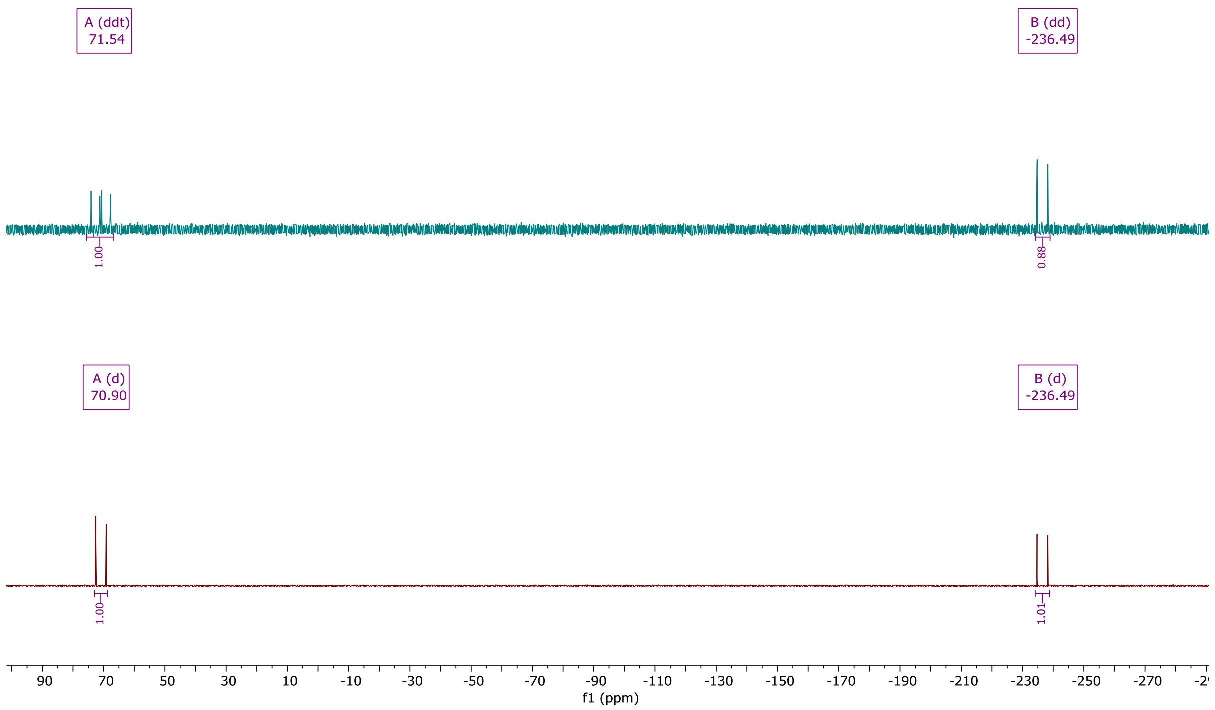

Figure S21. ^{31}P (top) and $^{31}P\{^{1}H\}$ (bottom) NMR spectra (162 MHz, 293 K) of **4** in C_6D_6.

1.8. Synthesis of $[CH_2\{N(Dipp)\}]_2P(H)PGa(PHPh)(NacNac)$ (5)

A (50 mg, 0.054 mmol) was dissolved in toluene and a solution of phenylphosphine in hexanes (10% by w/w) (1 mL, ~0.9 mmol) was added. The mixture immediately decolourised and the solvent was removed under reduced pressure. Recrystallisation from hexane gave yellow crystalline solids. (0.017 g, 26 %). Anal. calculated for $C_{61}H_{86}GaN_4P_3$: C, 70.59; H, 8.35; N, 5.40. Found: C, 70.75; H, 8.54; N, 5.45. **^{1}H NMR (400 MHz, C_6D_6):** δ (ppm) 9.41 (ddd, $^1J_{P–H}$ = 463.6, $^2J_{P–H}$ = 9.4, $^4J_{P–H}$ = 3.0 Hz, 1H; P*H*), 7.83 (t, $^3J_{H–H}$ = 6.4 Hz, 1H; PAr*H*), 7.36–6.95 (m, 16H; Ar*H*), 4.77 (s, 1H; NacNac γ-H), 3.86 (sept, $^3J_{H–H}$ = 6.9 Hz, 2H; {C*H*(CH_3)$_2$}), 3.43 (d, $^1J_{P–H}$ = 212.2 Hz, 1H; PhP*H*), 3.58–3.26 (m, 10H; {C*H*(CH_3)$_2$} and {(NCH_2)$_2$}), 1.42 (s, 6H; NacNac C*H*$_3$), 1.39–0.93 (m, 42H; {CH(C*H*$_3$)$_2$}). **^{13}C{^{1}H} NMR (126 MHz, C_6D_6):** δ (ppm) 169.68 (NacNac *C*), 150.49 (Ar*C*), 149.54 (Ar*C*), 145.27 (Ar*C*), 143.89 (Ar*C*), 142.87 (Ar*C*), 139.73 (d, *J* = 30.2 Hz; PAr*C*), 136.80 (d, $^2J_{P–C}$ = 5.2 Hz; Ar*C*), 133.98 (d, *J* = 12.2 Hz; Ar*C*), 127.01 (Ar*C*), 124.90 (d, *J* = 10.9 Hz; Ar*C*), 124.76 (Ar*C*), 124.19 (Ar*C*), 123.96 (Ar*C*), 98.70 (NacNac *C*H), 51.56 ({(NCH_2)$_2$}), 51.50 ({(NCH_2)$_2$}), 31.97 (Dipp {CH(*C*H$_3$)$_2$}), 29.40 (Dipp {CH(*C*H$_3$)$_2$}), 29.33 (d, *J* = 5.3 Hz; Dipp {*C*H(CH_3)$_2$}), 29.17 (d, $^4J_{P–C}$ = 5.5 Hz; Dipp {*C*H(CH_3)$_2$}), 27.78 (Dipp {CH(*C*H$_3$)$_2$}), 26.59 (Dipp {CH(*C*H$_3$)$_2$}), 26.14 (Dipp {CH(*C*H$_3$)$_2$}), 25.25 (Dipp {CH(*C*H$_3$)$_2$}), 24.85 (d, $^5J_{P–C}$ = 7.1 Hz; Dipp {CH(*C*H$_3$)$_2$}), 24.76 (Dipp {CH(*C*H$_3$)$_2$}), 24.53 (NacNac *C*H$_3$), 24.44 (Dipp {CH(*C*H$_3$)$_2$}), 23.06 (Dipp {CH(*C*H$_3$)$_2$}), 14.36 (Dipp {CH(*C*H$_3$)$_2$}). **^{31}P NMR (162 MHz, C_6D_6):** δ (ppm) 60.11 (ddd, $^1J_{P–P}$ = 579.9 Hz, $^1J_{P–H}$ = 463.8 Hz, $^3J_{P–P}$ = 53.9 Hz), –109.70 (ddd, $^1J_{P–H}$ = 211.9 Hz, $^3J_{P–P}$ = 54.1 Hz, $^2J_{P–P}$ = 19.4 Hz), –210.76 (ddd, $^1J_{P–P}$ =580.2 Hz, $^2J_{P–P}$ = 19.3 Hz, $^2J_{P–H}$ = 10.1 Hz).

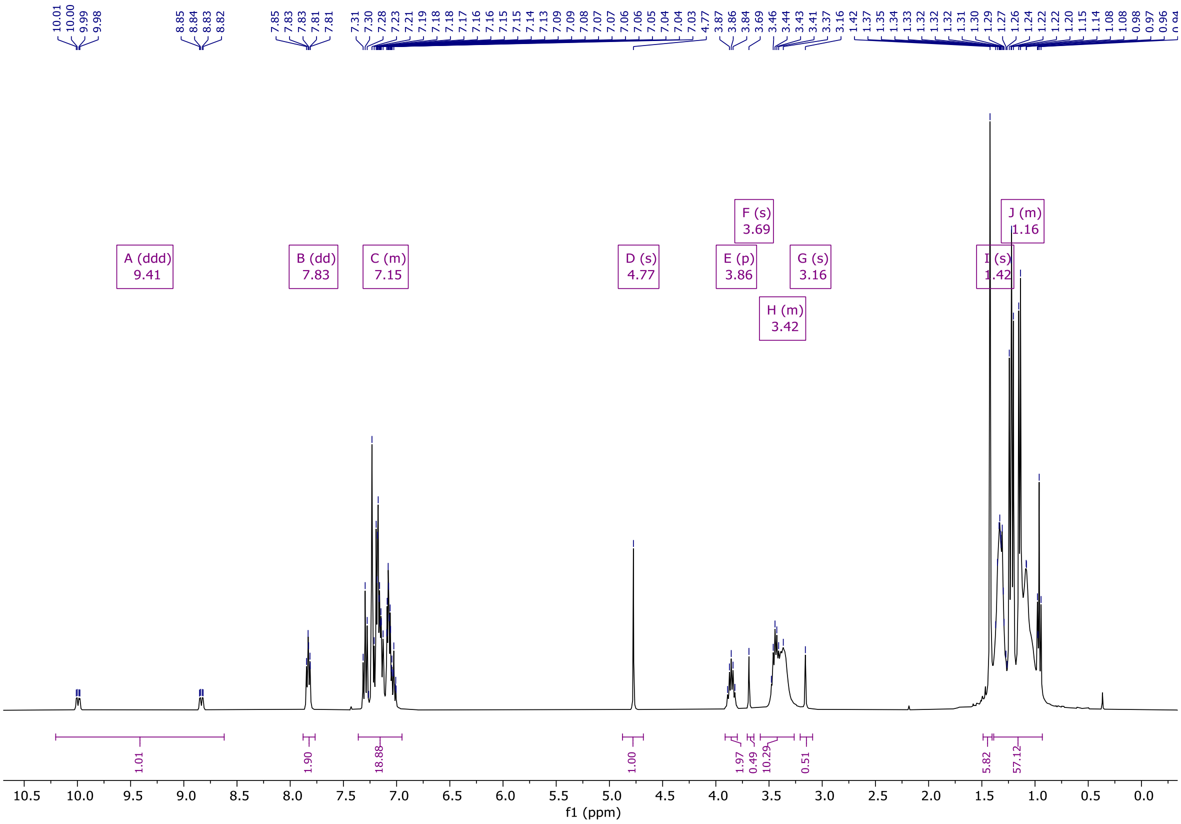

Figure S22. ^{1}H NMR spectrum (400 MHz, 293 K) of **5** in C_6D_6.

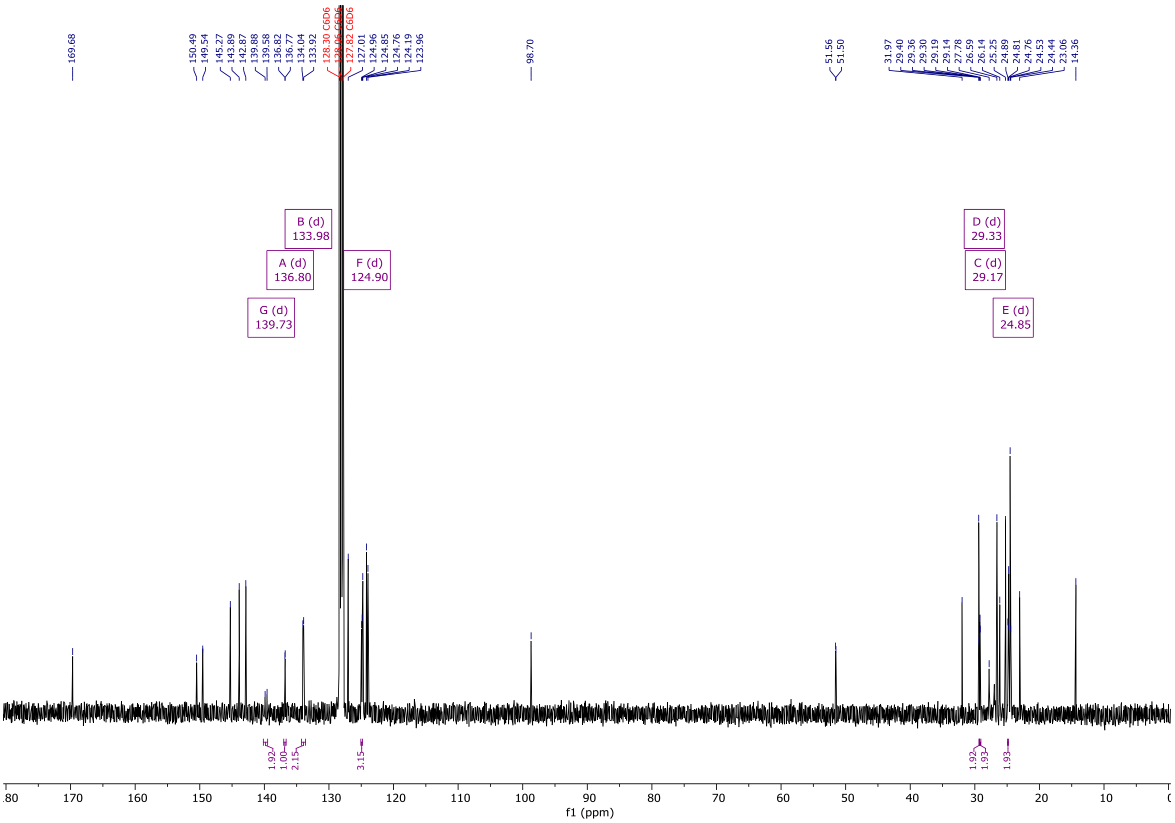

Figure S23. $^{13}C\{^{1}H\}$ NMR spectrum (126 MHz, 293 K) of **5** in C_6D_6.

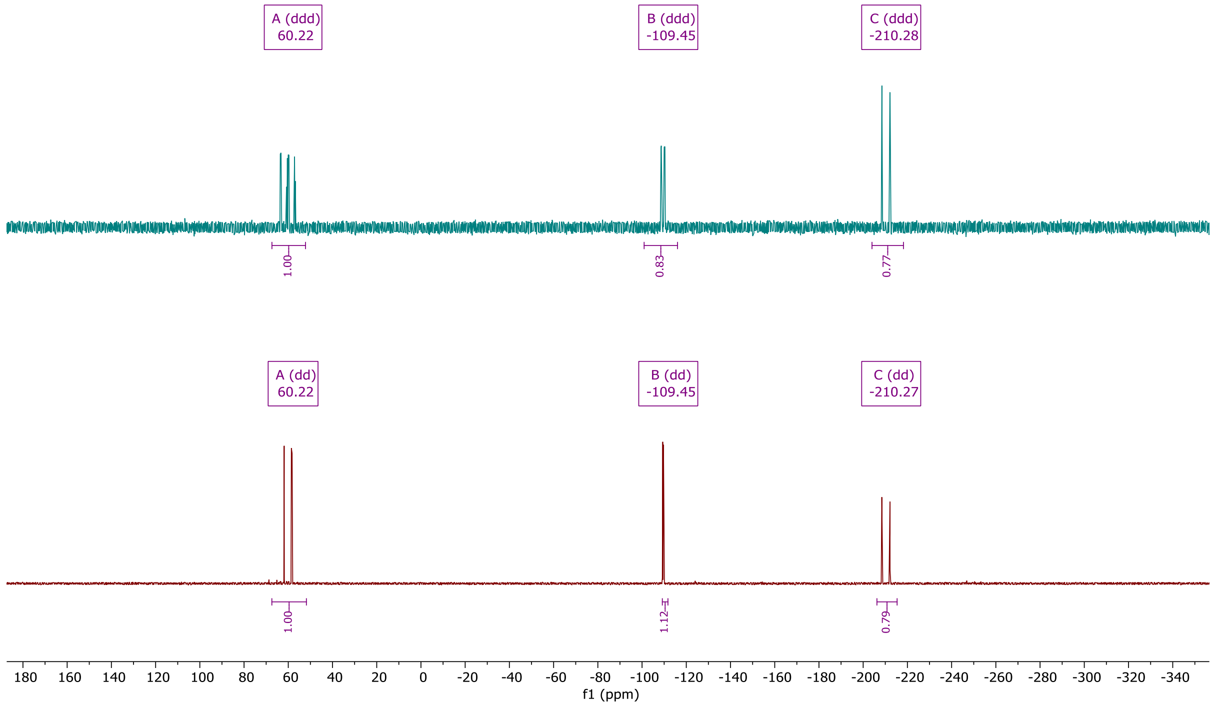

Figure S24. ^{31}P (top) and ^{31}P{^{1}H} (bottom) NMR spectra (162 MHz, 293 K) of **5** in C_6D_6.

1.10. Reaction of 1c with PhCCD

1c (5 mg, 0.00529 mmol) was dissolved in C_6D_6 and a stoichiometric amount of PhCCD was added from a C_6D_6 stock solution 0.05255 mol·L^{-1} (0.10 mL, 0.00529 mmol). The NMR spectrum was obtained without further purification. The sample can be further purified from recrystallisation in hexane.

^{31}P NMR (162 MHz, C_6D_6): δ 70.20 (dt, $^{1}J_{P–P}$ = 568.1 Hz, $^{1}J_{P–D}$ = 71.8 Hz), –238.25 (d, J = 565.7 Hz).

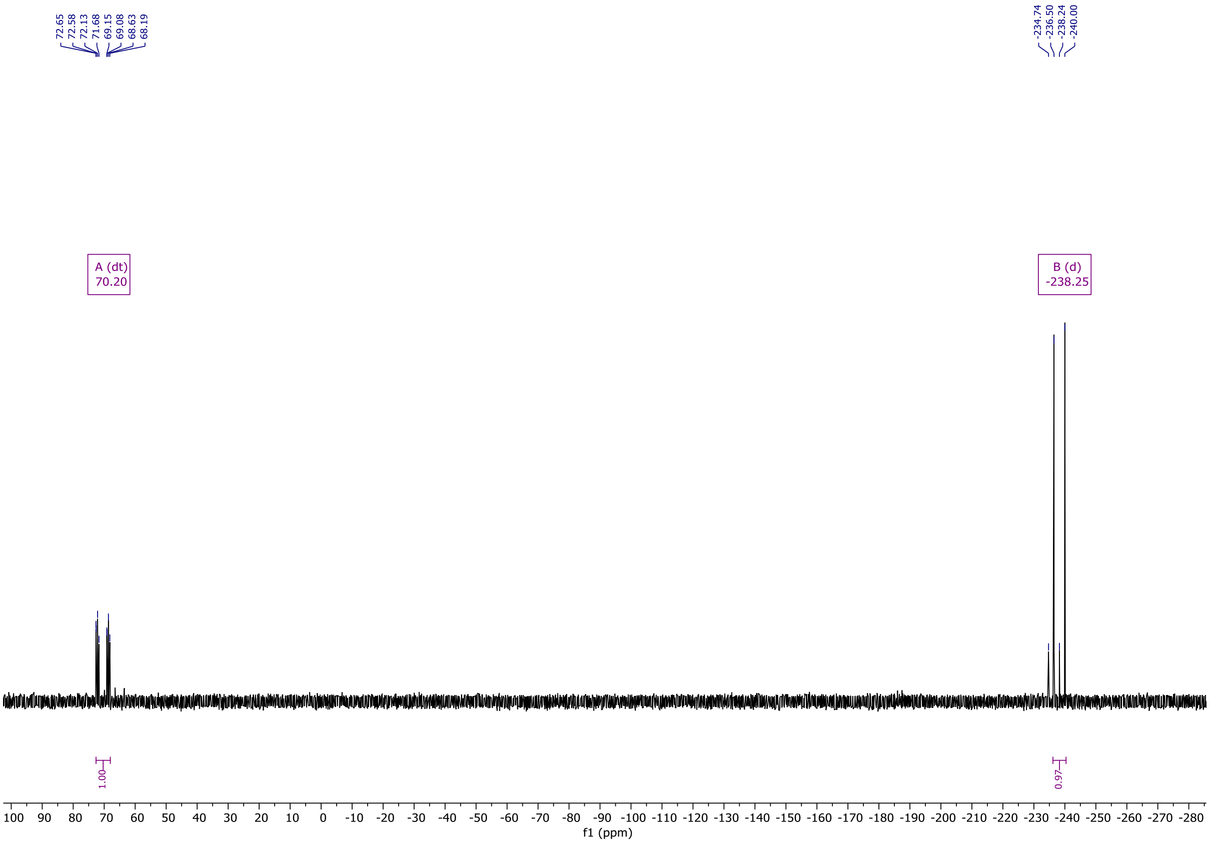

Figure S25. ^{31}P NMR spectrum (162 MHz, 293 K) of the reaction of **1c** with PhCCD in C_6D_6.

1.11. Reaction of 1c with $B(C_6F_5)_3$

1c (5 mg, 0.00529 mmol) and a stoichiometric amount of $B(C_6F_5)_3$ (3 mg, 0.0053 mmol) was dissolved in C_6D_6. The solution immediately turned orange. The NMR spectrum was obtained without further purification. The signals at 157.86 ppm and –61.37 ppm were identified as **A**. The other signals were identified as the Lewis acid-base adduct of **2**:BCF.

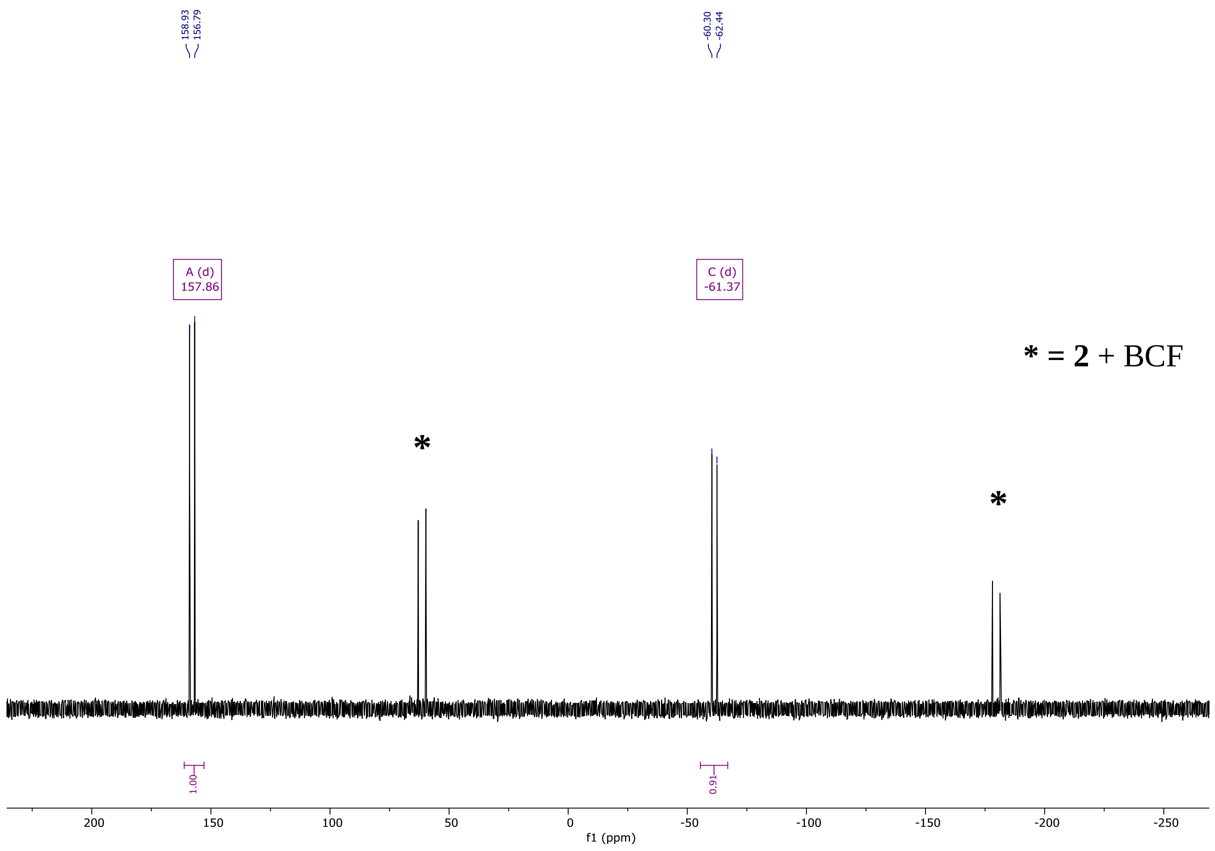

Figure S26. ^{31}P NMR spectrum (162 MHz, 293 K) of the reaction of **1c** with PhCCD in C_6D_6.

1.12. Synthesis of $[CH_2\{N(Dipp)\}]_2PP(H)Ga(H_2SiPh)(NacNac)$ (6)

A (50 mg, 0.054 mmol) was dissolved in toluene and a drop of phenylsilane was added. The mixture immediately decolourised and the solvent was removed under reduced pressure. Recrystallisation from hexane gave white crystalline solids. (0.033 g, 59.1 %). Anal. calculated for $C_{61}H_{87}GaN_4P_2Si$: C, 70.71; H, 8.46; N, 5.41. Found: C, 71.62; H, 8.37; N, 6.04. **^{1}H NMR (400 MHz, C_6D_6)**: δ (ppm) 7.30–6.95 (m, 17H; ArC*H*), 5.68 (d, $^2J_{P–H}$ = 33.1 Hz, 1H; Ga*H*), 5.22 (dd, $^2J_{P–H}$ = 6.3 Hz, 2H; Si*H*), 4.74 (s, 1H; NacNac γ-H), 4.18 (m, 2H; $\{(NCH_2\}_2\}$), 3.69 (sept, $^3J_{H–H}$ = 6.7 Hz, 2H; $\{CH(CH_3)_2\}$), 3.41 (sept, $^3J_{H–H}$ = 6.9 Hz, 2H; $\{CH(CH_3)_2\}$), 3.33–3.14 (m, 8H; $\{CH(CH_3)_2\}$ and $\{(NCH_2)\}_2\}$), 1.40 (s, 6H; NacNac CH_3), 1.30 (d, $^3J_{H–H}$ = 6.7 Hz, 6H; $\{CH(CH_3)_2\}$), 1.20 (d, $^3J_{H–H}$ = 6.7 Hz, 6H; $\{CH(CH_3)_2\}$), 1.16 (d, $^3J_{H–H}$ 6.7 Hz, 6H; $\{CH(CH_3)_2\}$), 1.11–1.02 (m, 24H; $\{CH(CH_3)_2\}$), 0.98 (d, $^3J_{H–H}$ = 6.8 Hz, 6H; $\{CH(CH_3)_2\}$).

^{13}C NMR (126 MHz, C_6D_6): δ (ppm) 169.08 (NacNac *C*), 150.12 (Ar*C*), 150.10 (Ar*C*), 148.53 (Ar*C*), 144.40 (Ar*C*), 144.09 (Ar*C*), 143.39 (Ar*C*), 140.43 (d, $^{2}J_{P–C}$ = 12.6 Hz; Ar*C*), 136.69 (Ar*C*), 136.12 (d, $^{2}J_{P–C}$ = 15.2 Hz; Ar*C*), 129.17 (Ar*C*), 127.29 (Ar*C*), 127.22 (Ar*C*), 124.65 (dd, $^{2}J_{P–C}$ = 55.4 Hz, $^{3}J_{P–C}$ = 35.6 Hz; PPSi*C*), 96.78 (NacNac *C*H), 56.66 ({(N*C*H_2}$_2$}), 56.60 ({(N*C*H_2}$_2$}), 29.71 (d, $^{4}J_{P–C}$ = 4.1 Hz; Dipp {*C*H(CH_3)$_2$}), 28.89 (d, $^{4}J_{P–C}$ = 3.0 Hz; Dipp {*C*H(CH_3)$_2$}), 28.68 (d, $^{4}J_{P–C}$ = 6.8 Hz; Dipp {*C*H(CH_3)$_2$}), 28.49 (Dipp {*C*H(CH_3)$_2$}), 27.42 (Dipp {CH(*C*H_3)$_2$}), 27.08 (Dipp {CH(*C*H_3)$_2$}), 24.99 (Dipp {CH(*C*H_3)$_2$}), 24.94 (Dipp {CH(*C*H_3)$_2$}), 24.74 (Dipp {CH(*C*H_3)$_2$}), 24.16 (Dipp {CH(*C*H_3)$_2$}), 24.12 (Dipp {CH(*C*H_3)$_2$}), 24.01 (Dipp {CH(*C*H_3)$_2$}), 23.82 (Dipp {CH(*C*H_3)$_2$}). **^{31}P NMR (162 MHz, C_6D_6):** δ (ppm) 162.52 (d, $^{1}J_{P–P}$ = 388.9 Hz), –173.64 (d, $^{1}J_{P–P}$ = 389.0 Hz). **^{29}Si NMR (99 MHz, C_6D_6):** δ (ppm) –36.02. ^{29}Si NMR signal was obtained from ^{1}H/^{29}Si HMBC experiment at 99MHz.

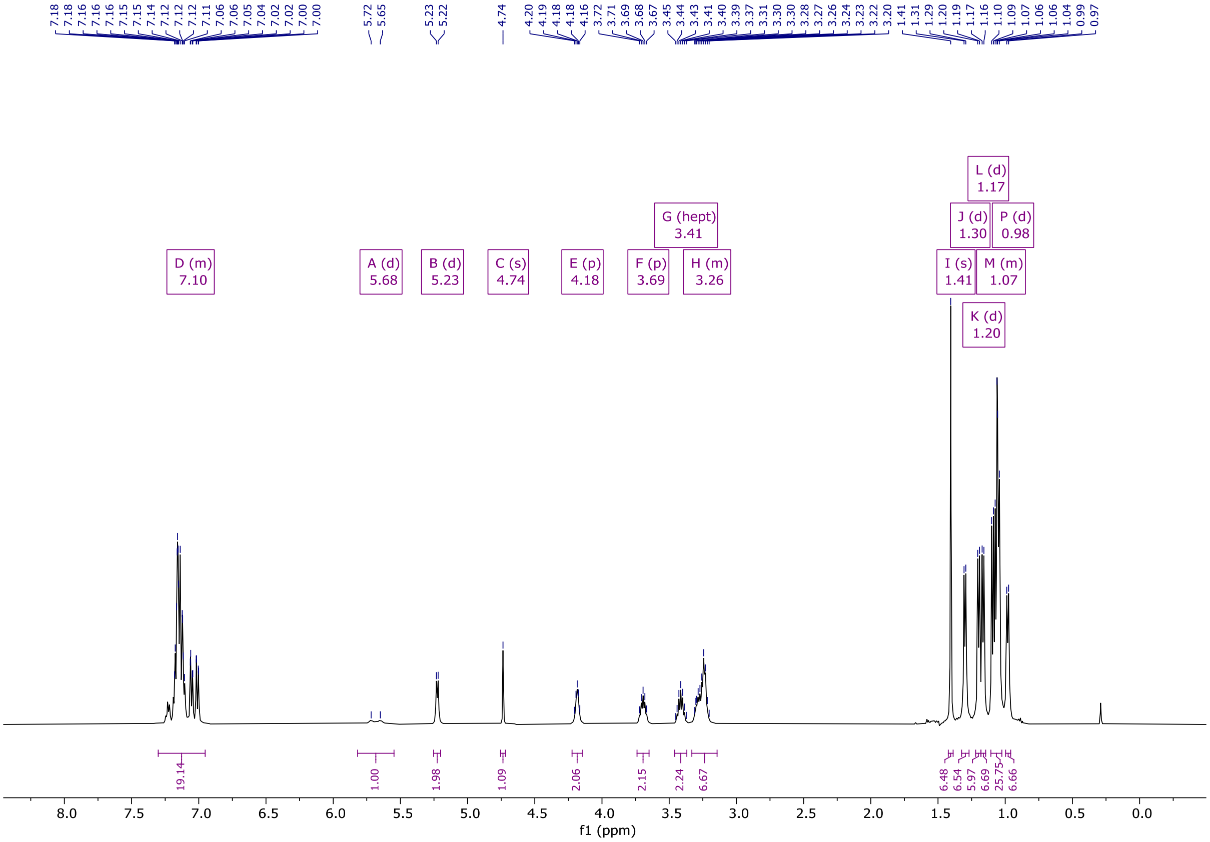

Figure S27. ^{1}H NMR spectrum (400 MHz, 293 K) of **6** in C_6D_6.

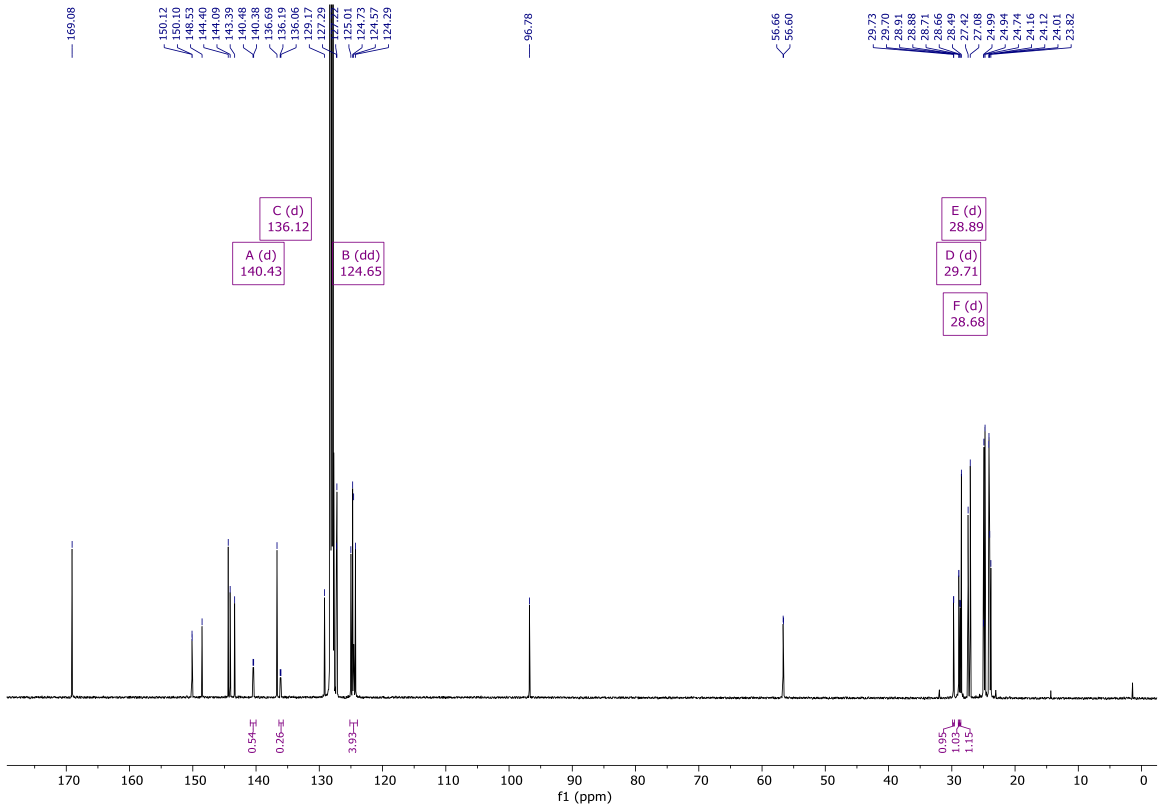

Figure S28. $^{13}C\{^{1}H\}$ NMR spectrum (126 MHz, 293 K) of **6** in C_6D_6.

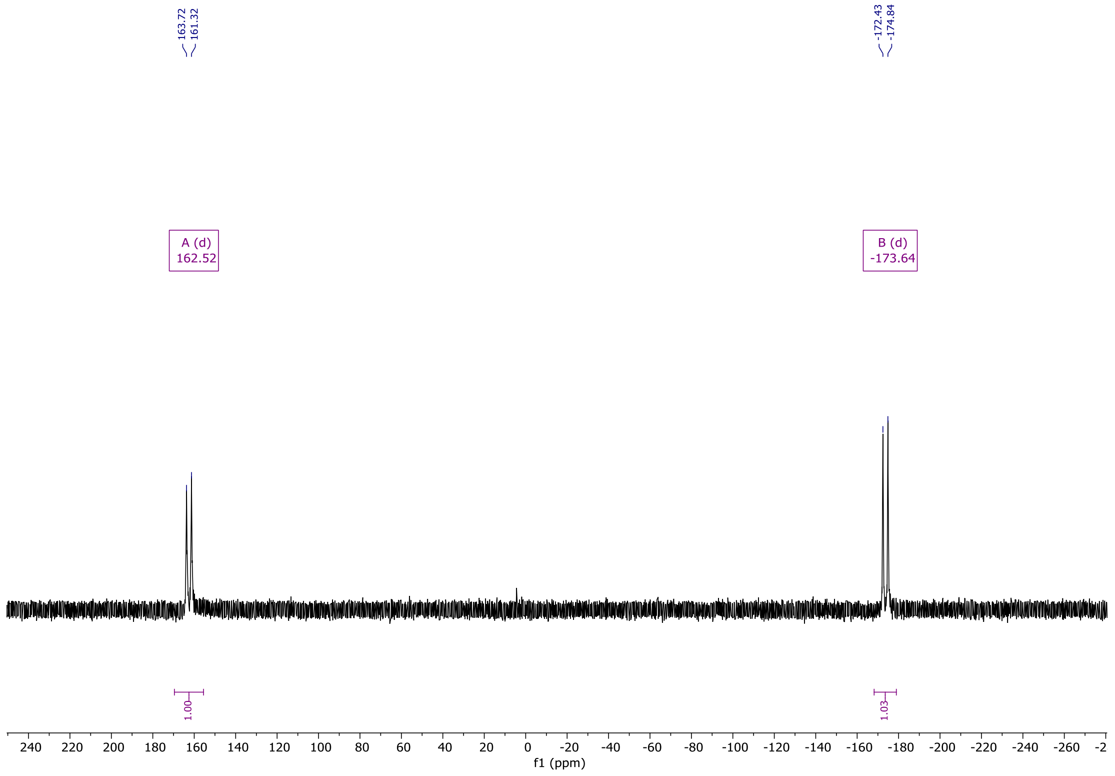

Figure S30. $^{31}P\{^{1}H\}$ NMR spectrum (126 MHz, 293 K) of **6** in C_6D_6.

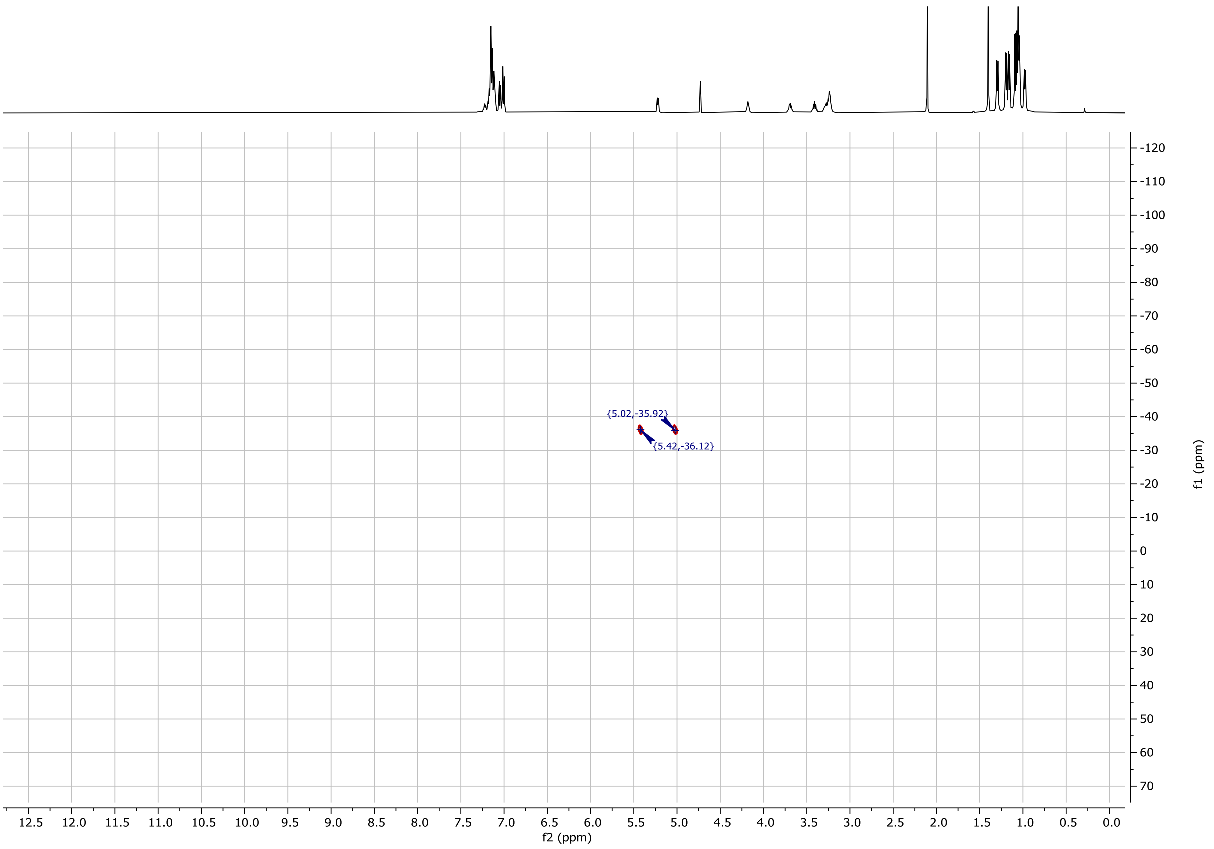

Figure S31. $^{1}H/^{29}Si$ HMBC NMR spectrum (99 MHz, 293 K) of **6** in C_6D_6.

2. Single crystal X-ray diffraction data

Single-crystal X-ray diffraction data were collected using an Oxford Diffraction Supernova dual-source diffractometer equipped with a 135 mm Atlas CCD area detector. Crystals were selected under Paratone-N oil, mounted on micromount loops and quench-cooled using an Oxford Cryosystems open flow N_2 cooling device. Data were collected at 150 K using mirror monochromated Cu Kα (λ = 1.54184 Å) or Mo Kα (λ = 0.71073 Å) radiation and processed using the CrysAlisPro package, including unit cell parameter refinement and inter-frame scaling (which was carried out using SCALE3 ABSPACK within CrysAlisPro).[2] Equivalent reflections were merged and diffraction patterns processed with the CrysAlisPro suite. Structures were subsequently solved using direct methods.[3]

Table S1. Selected X-ray data collection and refinement parameters for **1a**, **1b**·1.5hex, **1c**, and **2**.

	**1a**	**1b**·1.5hex	**1c**	**2**
Formula	$C_{61}H_{86}GaN_5P_2$	$C_{67}H_{109}GaN_5P_2$	$C_{55}H_{82}GaN_5P_2$	$C_{55}H_{81}GaN_4OP_2$
CCDC	2095915	2095916	2095917	2095918
Fw [g mol^{-1}]	1021.00	1116.25	944.91	945.89
Crystal system	monoclinic	triclinic	monoclinic	monoclinic
Space group	$C2/c$	$P\bar{1}$	$P2_1/c$	$P2_1/c$
a (Å)	41.0691(5)	12.7292(3)	19.9042(5)	19.9665(3)
b (Å)	14.4950(2)	12.8046(3)	12.5842(3)	12.5735(2)
c (Å)	21.7335(2)	20.9763(4)	21.6997(5)	21.8419(3)
α (°)	90	77.551(2)	90	90
β (°)	107.680(1)	79.616(2)	97.522(2)	103.334(2)
γ (°)	90	86.556(2)	90	90
V (Å^3)	12326.8(3)	436.927(18)	5388.5(2)	5335.56(14)
Z	8	2	4	4
Radiation, λ (Å)	Cu Kα, 1.54184	Mo Kα, 0.71073	Mo Kα, 0.71073	Cu Kα, 1.54184
Temp (K)	150(2)	150(2)	150(2)	150(2)
ρ_{calc} (g cm^{-3})	1.100	1.129	1.165	1.178
μ (mm^{-1})	1.386	0.509	0.609	1.570
Reflections collected	29690	54428	42363	33002
Independent reflections	12814	11536	9456	10991
Parameters	648	763	598	600
R(int)	0.0269	0.0257	0.0486	0.0348
R1/wR2,[a] I ≥ 2σI (%)	3.92/9.92	4.84/12.39	4.03/8.98	4.83/12.03
R1/wR2,[a] all data (%)	4.54/10.40	5.28/12.59	5.99/9.55	6.22/13.27
GOF	1.032	1.097	1.038	1.018

R1 = $[\Sigma||F_o| - |F_c||]/\Sigma|F_o|$; wR2 = $\{[\Sigma w[(F_o)^2 - (F_c)^2]^2]/[\Sigma w(F_o^2)^2]\}^{1/2}$; w = $[\sigma^2(F_o)^2 + (AP)^2 + BP]^{-1}$, where P = $[(F_o)^2 + 2(F_c)^2]/3$ and the A and B values are 0.0458 and 11.61 for **1a**, 0.0457 and 5.60 for **1b**·1.5hex, 0.0367 and 4.23 for **1c**, and 0.0599 and 6.87 for **2**.

Table S2. Selected X-ray data collection and refinement parameters for **3b**·1.5hex, **4**·0.5hex, **5**·hex and **6**.

	**3b**·1.5hex	**4**·hex	**5**·0.5hex	**6**
Formula	$C_{67}H_{109}GaN_5P_2$	$C_{69}H_{99}GaN_4P_2$	$C_{64}H_{93}GaN_4P_3$	$C_{61}H_{87}GaN_4P_2Si$
CCDC	2095919	2095920	2095921	2095922
Fw [g mol^{-1}]	1116.25	1116.18	1081.05	1036.09
Crystal system	triclinic	triclinic	monoclinic	triclinic
Space group	$P\bar{1}$	$P\bar{1}$	$P2_1/c$	$P\bar{1}$
a (Å)	12.6722(3)	12.9589(3)	22.8997(2)	11.8302(2)
b (Å)	12.7638(2)	14.8587(4)	13.1654(2)	19.9538(3)
c (Å)	20.7022(5)	19.3751(4)	20.6272(2)	25.2185(3)
α (°)	80.864(2)	70.031(2)	90	91.2230(10)
β (°)	88.304(2)	72.794(2)	97.7630(10)	102.7580(10)
γ (°)	88.978(2)	71.847(2)	90	93.5110(10)
V (Å^3)	3304.27(12)	3256.11(15)	6161.77(12)	5791.43(15)
Z	2	4	4	4
Radiation, λ (Å)	Cu Kα, 1.54184	Cu Kα, 1.54184	Cu Kα, 1.54184	Cu Kα, 1.54184
Temp (K)	150(2)	150(2)	150(2)	150(2)
ρ_{calc} (g cm^{-3})	1.122	1.138	1.165	1.188
μ (mm^{-1})	1.326	1.346	1.645	1.668
Reflections collected	36209	57270	38065	101237
Independent reflections	13646	13556	12744	23920
Parameters	704	744	675	1303
R(int)	0.0280	0.0457	0.0425	0.0492
R1/wR2,[a] I ≥ 2σI (%)	4.35/11.30	3.87/9.50	4.41/11.55	3.37/8.95
R1/wR2,[a] all data (%)	4.96/11.87	5.44/10.52	5.04/12.24	3.83/9.38
GOF	1.040	1.013	1.033	1.034

$R1 = [\Sigma||F_o| - |F_c||]/\Sigma|F_o|$; $wR2 = \{[\Sigma w[(F_o)^2 - (F_c)^2]^2]/[\Sigma w(F_o{}^2)^2\}^{1/2}$; $w = [\sigma^2(F_o)^2 + (AP)^2 + BP]^{-1}$, where $P = [(F_o)^2 + 2(F_c)^2]/3$ and the A and B values are 0.059 and 2.04 for **3**·1.5hex, 0.0505 and 1.58 for **4**·hex, 0.0683 and 2.69 for **5**·0.5hex, and 0.0498 and 2.27 for **6**.

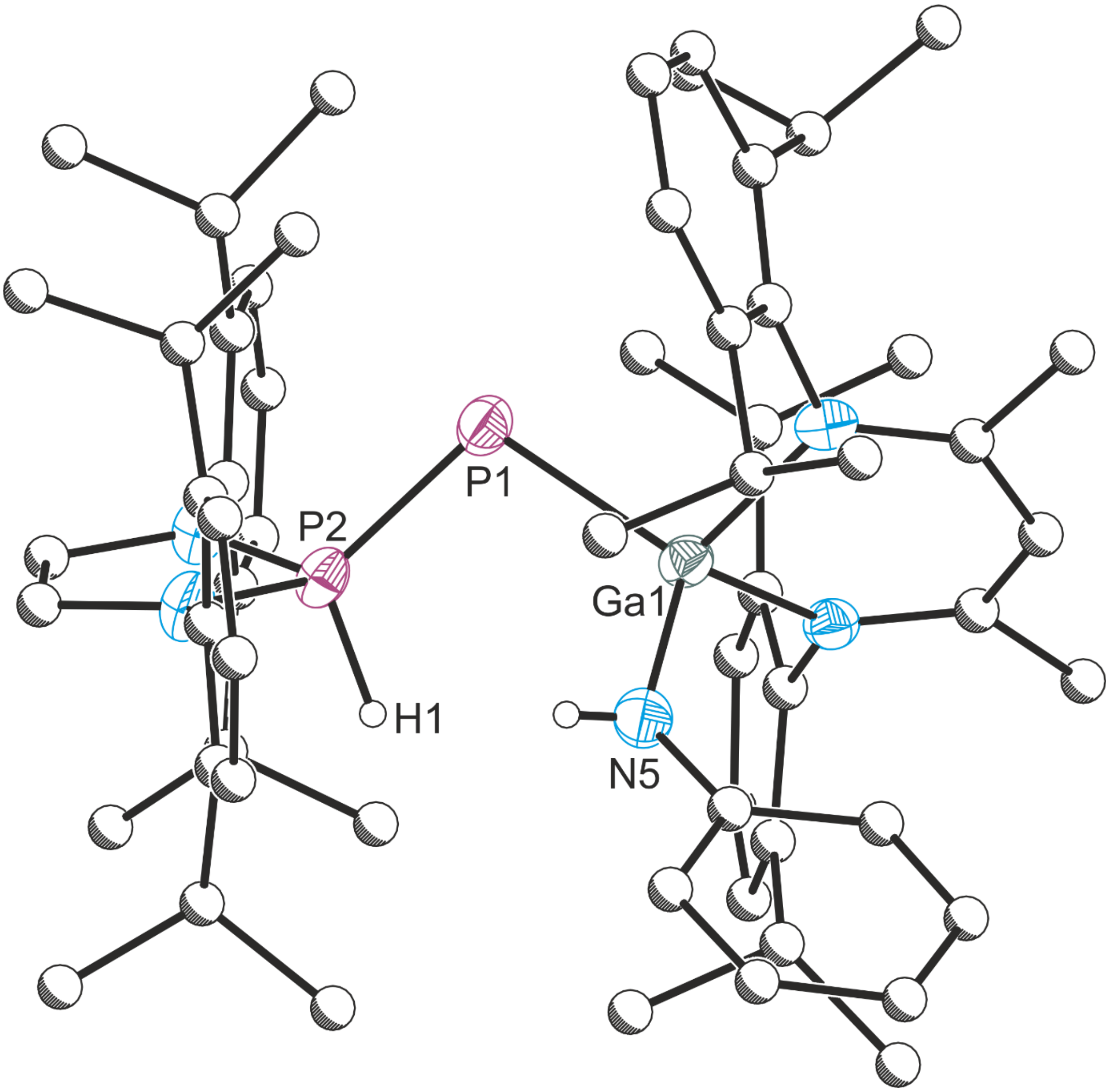

Figure S32. Molecular structure of **1a**. Ellipsoids set at 50% probability; hydrogen atoms (with the exception of those originating from the substrate) omitted for clarity. All carbon atoms are pictured as spheres of arbitrary radius. Selected interatomic distances [Å] and angles [°]: Ga1–P1 2.303(1), P1–P2 2.045(1), Ga1–N5 1.884(2), P2–H1 1.36(2), Ga1-P1-P2 104.78(2).

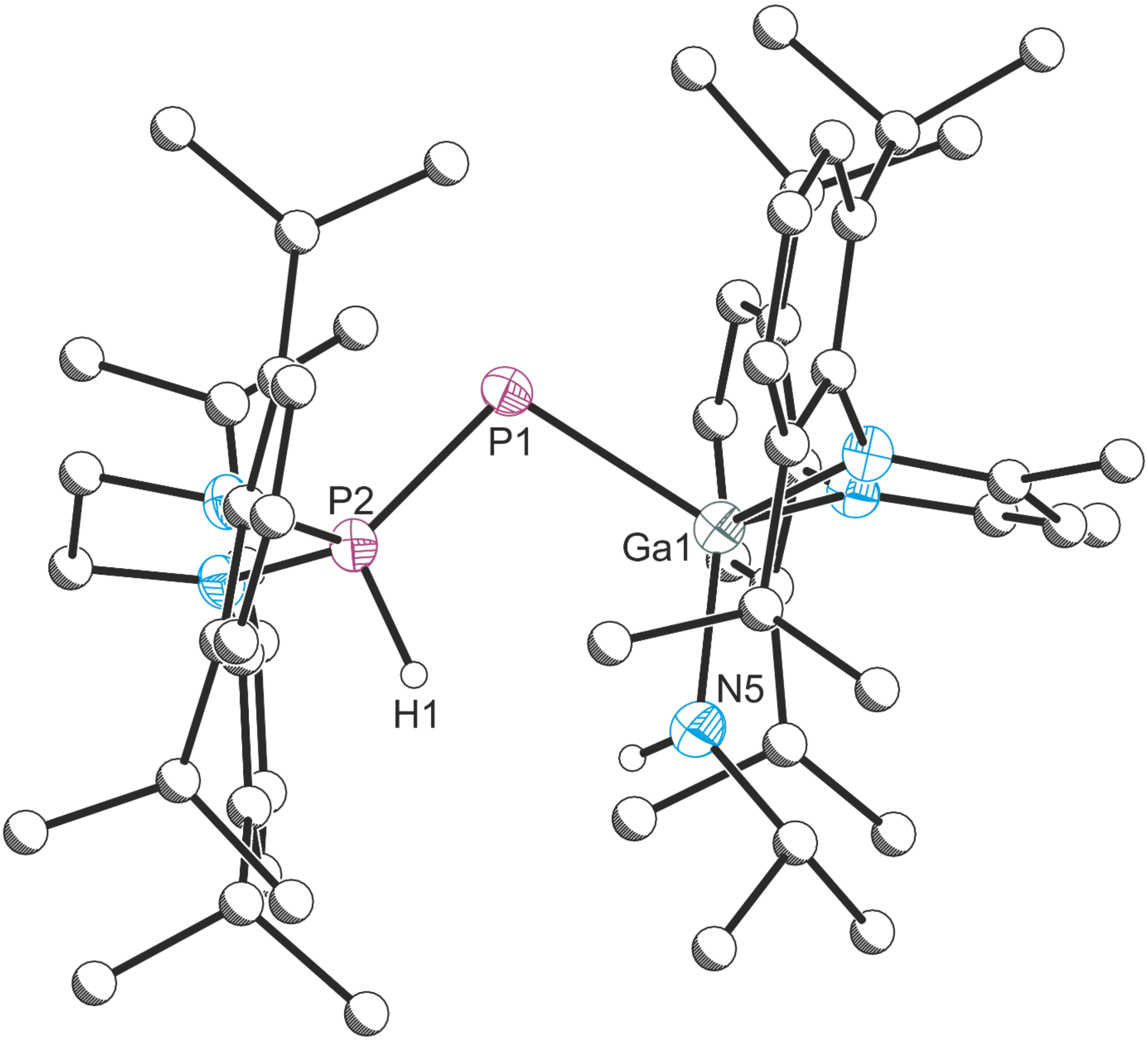

Figure S33. Molecular structure of **1b**. Ellipsoids set at 50% probability; hydrogen atoms (with the exception of those originating from the substrate) omitted for clarity. All carbon atoms are pictured as spheres of arbitrary radius. Selected interatomic distances [Å] and angles [°]: Ga1–P1 2.314(1), P1–P2 2.067(1), Ga1–N5 1.865(2), P2–H1 1.38(6), Ga1-P1-P2 99.94(3).

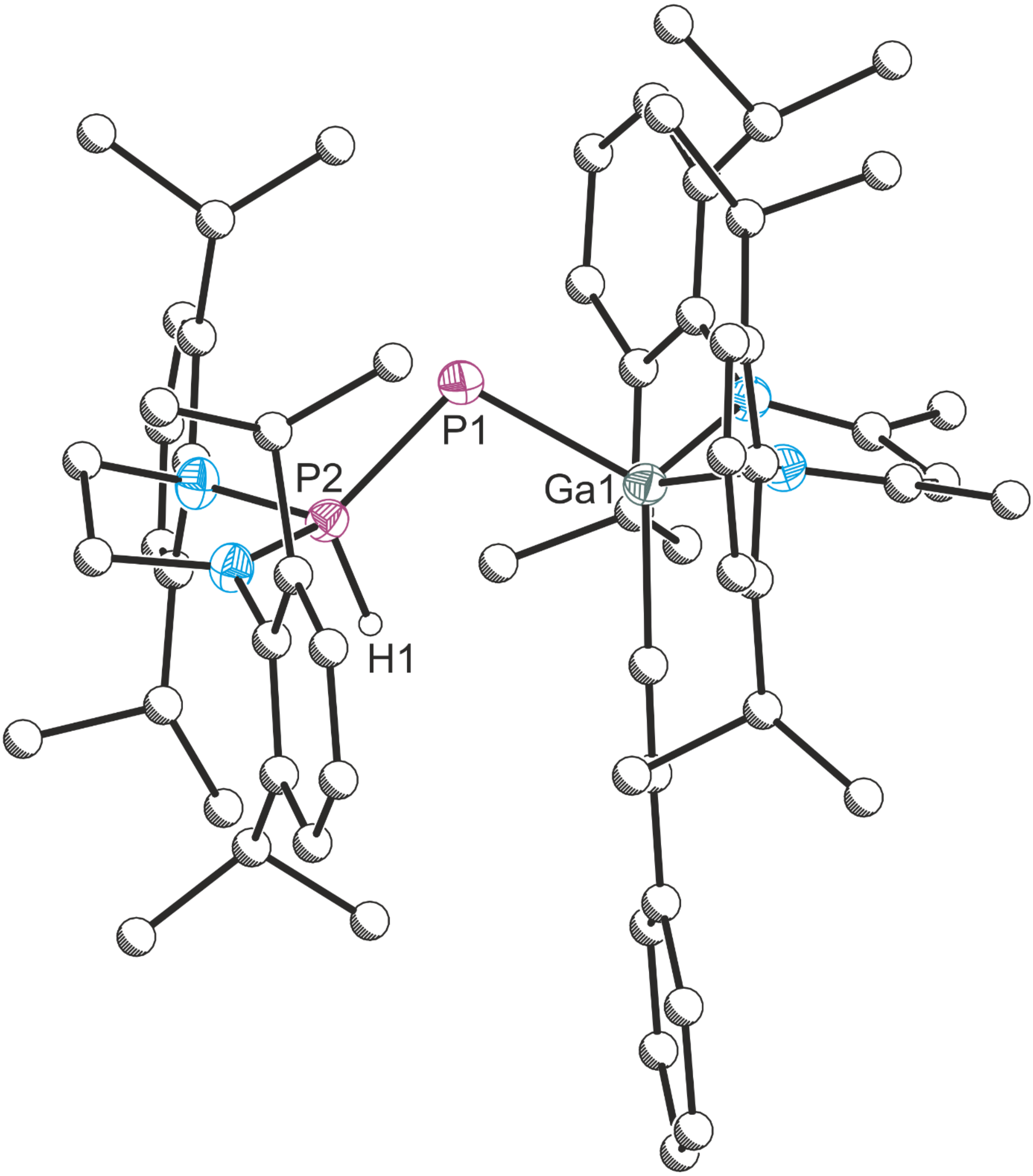

Figure S34. Molecular structure of **4**. Ellipsoids set at 50% probability; hydrogen atoms (with the exception of those originating from the substrate) omitted for clarity. All carbon atoms are pictured as spheres of arbitrary radius. Selected interatomic distances [Å] and angles [°]: Ga1–P1 2.283(1), P1–P2 2.051(1), Ga1–C56 1.964(2), P2–H1 1.33(2), Ga1-P1-P2 100.80(2).

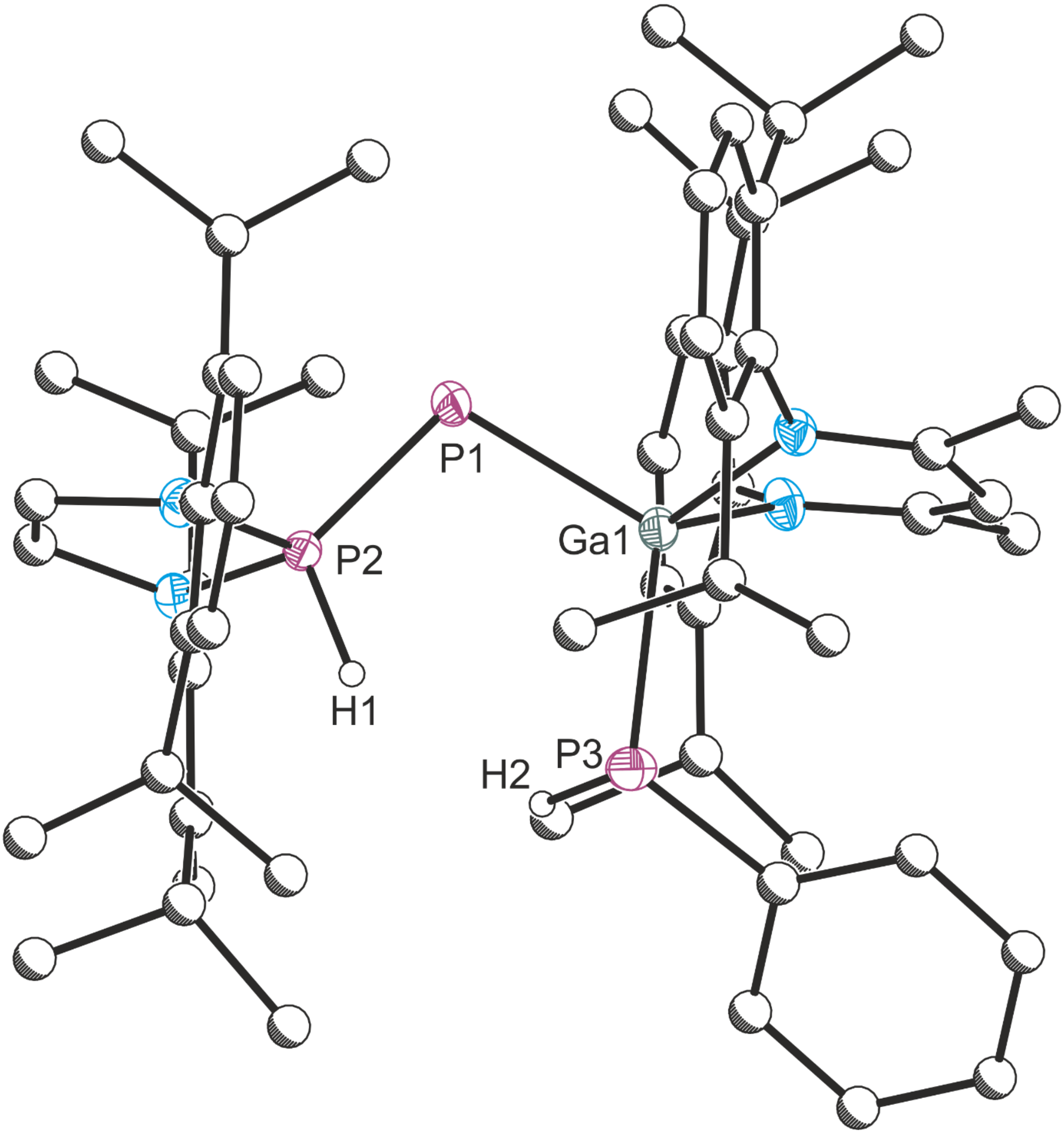

Figure S35. Molecular structure of **5**. Ellipsoids set at 50% probability; hydrogen atoms (with the exception of those originating from the substrate) omitted for clarity. All carbon atoms are pictured as spheres of arbitrary radius. Selected interatomic distances [Å] and angles [°]: Ga1–P1 2.303(1), P1–P2 2.049(1), Ga1–P3 2.385(1), P2–H1 1.33(3), Ga1-P1-P2 102.22(2).

3. Computational Details

The quantum chemical DFT calculations were performed with the Gaussian 16.[4] Where possible, optimizations were performed starting from the single crystal X-ray structure. Diisopropopylphenyl groups were truncated to phenyl in all systems. The structures are fully optimized at the B3LYP with inclusion of empirical dispersion correction (GD3), and the def2-TZVP(Ga, P, N)/Def2-SVP(C, H) basis set. The optimized structures are characterized by frequency analysis to identify the nature of the located stationary points. Transition states were identified by relaxed surface scans using only the Def2-SVP basis set and confirmed by the presence of a single imaginary frequency. Single point calculations were performed on the identified TS structures using the def2-TZVP (Ga, P, N)/Def2-SVP (C, H) basis set. Calculations were performed in the gas phase at 298.15 K and 1 atm.

	G [a.u.]	ΔG [a.u.]	ΔG [kcal/mol]
**A^{DFT}**	-4025.536365		
**NH_3**	-56.563921		
**A^{DFT} + NH_3**	-4082.100286	0	0
**I1**	-4082.103447	-0.00316	-1.98353
**TS1**	-4082.078421	0.021865	13.72029
**$2c^{DFT}$**	-4082.109888	-0.0096	-6.02526
**TS2**	-4082.05967	0.040616	25.48654
**$3c^{DFT}$**	-4082.122584	-0.0223	-13.992
**$PhSiH_3$**	-522.732782		
**A^{DFT} + $PhSiH_3$**	-4548.269147	0	0

**TS3**	-4548.23096	0.038187	23.96234
**[1,3] product**	-4548.270171	-0.00102	-0.64256
**TS4**	-4548.239149	0.029998	18.823745
**[1,2] product**	-4548.296707	-0.02756	-17.2939

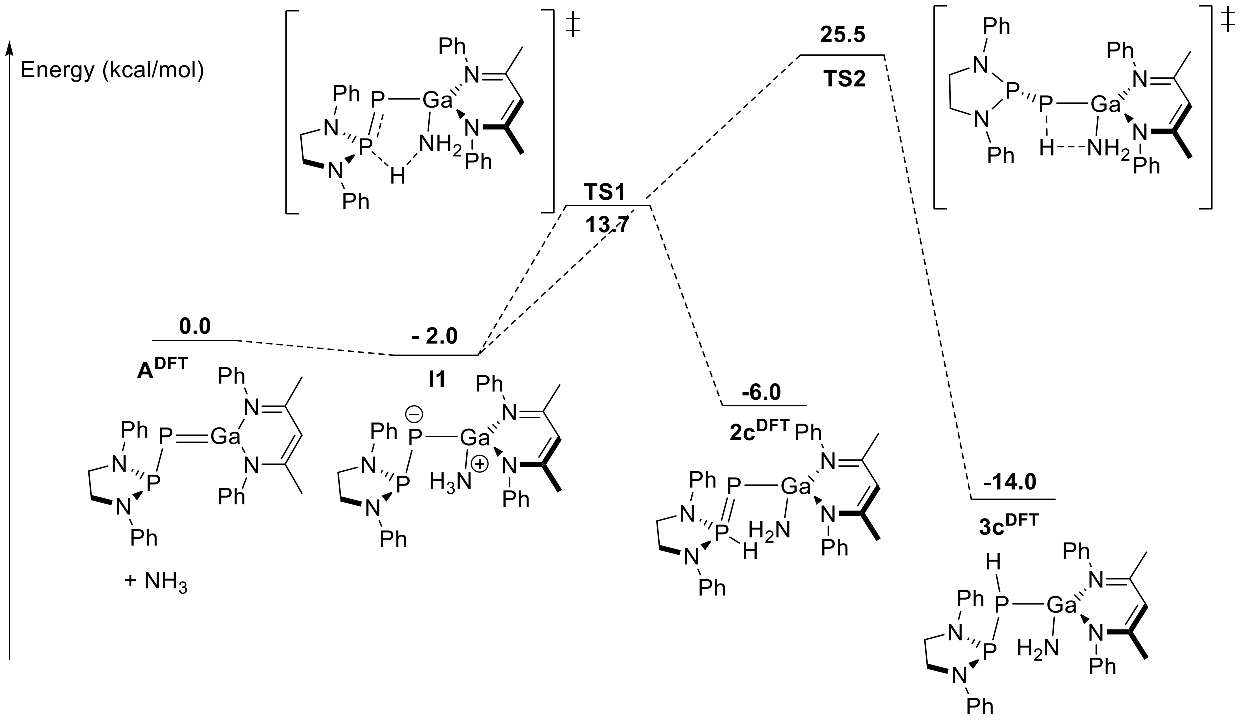

Figure S36. Computed mechanisms for the reaction of $\mathbf{A^{DFT}}$ with ammonia.

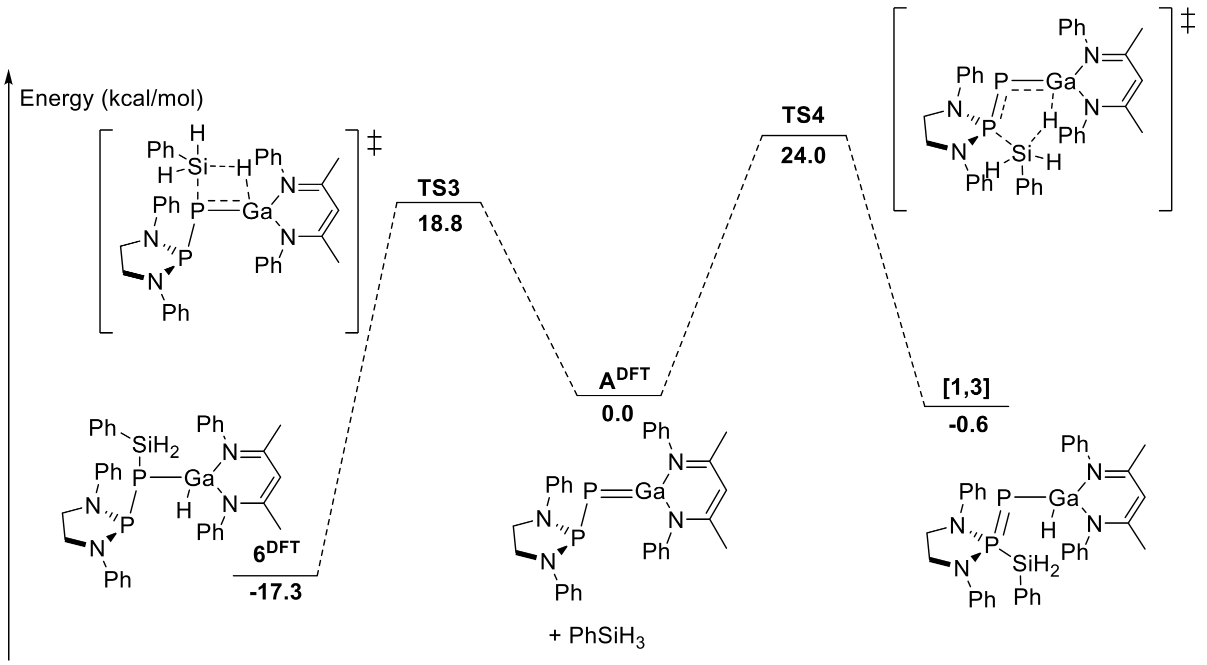

Figure S37. Computed mechanisms for the reaction of $\mathbf{A^{DFT}}$ with $PhSiH_3$.

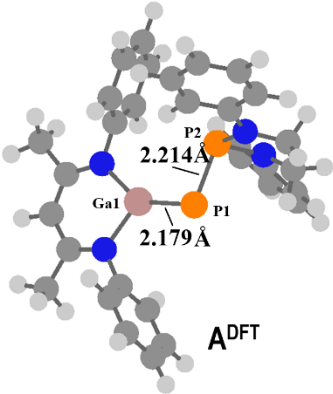

A^{DFT}

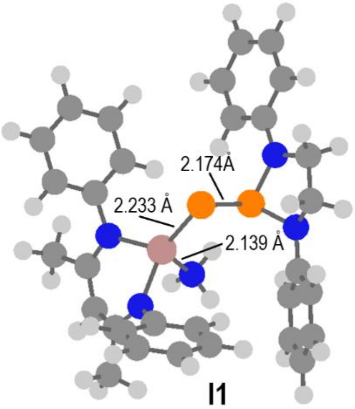

I1

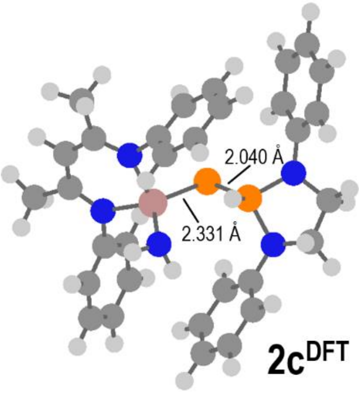

2c^{DFT}

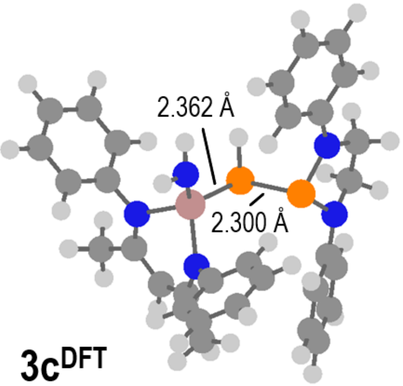

3c^{DFT}

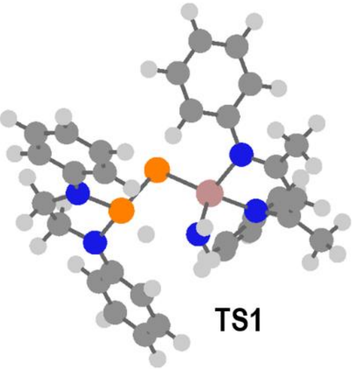

TS1

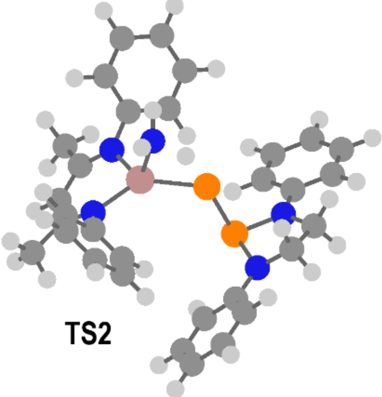

TS2

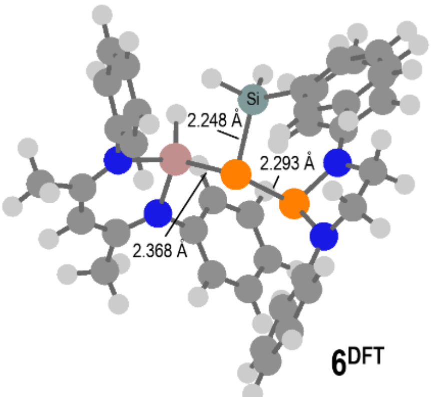

6^{DFT}

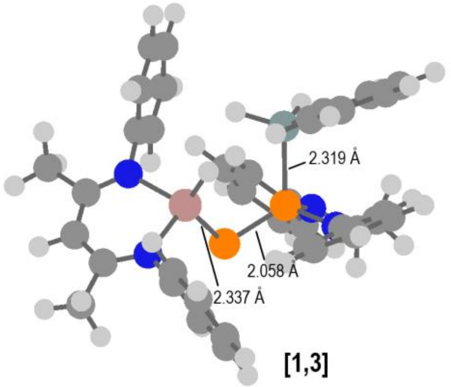

[1,3]

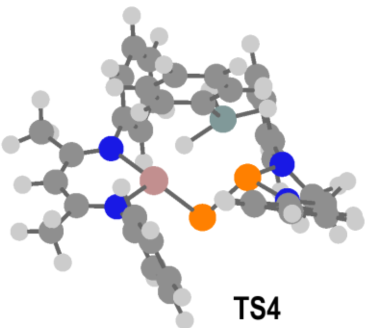

TS4

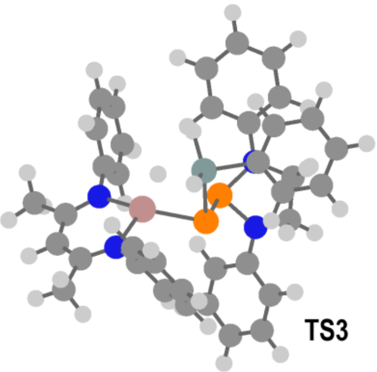

TS3

Coordinates

A^{DFT}

Ga	-1.30215100	0.44419900	0.22287500
P	1.78994100	-0.56488700	-0.07784300
P	0.01905400	-1.00198800	1.17774900
N	2.09373300	-2.11924500	-0.85051700
N	-3.21858000	0.46939200	0.59681000
N	3.08212100	-0.96071700	1.04925200
N	-1.31052800	2.02610400	-0.90681500
C	-4.07621400	1.36114000	0.10589300
C	1.43494300	-2.50666300	-2.00617800
C	3.70729200	-0.02405200	1.84672100
C	2.57909200	-3.08502800	0.10798100
H	3.07055400	-3.92784900	-0.40194300
H	1.75551400	-3.49197800	0.72953700
C	-3.68974200	2.42008000	-0.73260600
H	-4.48808900	3.08643000	-1.05487700
C	3.57702400	-2.31813300	0.96253600
H	3.66983900	-2.77549900	1.96332600
H	4.57927400	-2.33755600	0.49168500
C	1.08435300	-1.54584400	-2.97840000
C	4.89557300	-0.32555100	2.54434900
C	-2.40527900	2.73845400	-1.19395200
C	-3.67362700	-0.57194700	1.46214900
C	-0.03853900	2.43176800	-1.41680200
C	3.17274100	1.27400200	1.99757000
C	1.11317500	-3.85182000	-2.26841400
C	-3.71790800	-0.36505600	2.84370900
C	5.51933600	0.63453700	3.33767400
H	6.44128900	0.36773200	3.86183400
C	0.85176400	3.12182500	-0.59024200
C	-4.49114800	-2.63234800	3.16199600
H	-4.80789700	-3.43911100	3.82693300
C	-5.53090200	1.24579200	0.46126500
H	-5.92210100	0.25937900	0.17051900
H	-6.12183200	2.02605100	-0.03220000
H	-5.67132100	1.32434900	1.54973900
C	-4.01807300	-1.81982000	0.93537300
C	0.45110700	-1.92125900	-4.15608600
H	0.20181100	-1.15357400	-4.89469000
C	0.14699000	-3.25990100	-4.41412800
H	-0.34609100	-3.55133100	-5.34405200
C	-2.26388300	3.96437800	-2.05159300
H	-1.49827000	4.63851000	-1.63933000
H	-3.21590600	4.50256800	-2.12545700
H	-1.92798400	3.69807500	-3.06474400
C	0.34191100	2.09458100	-2.71865900
C	0.48729000	-4.21499600	-3.45960200
H	0.25390800	-5.26893900	-3.63450900
C	3.80989700	2.22097600	2.78888800
H	3.36534300	3.21568300	2.88710800
C	4.99136700	1.91679100	3.46797200
H	5.48523000	2.66379400	4.09302600
C	-4.13069700	-1.39299900	3.68860600
H	-4.15978600	-1.22555300	4.76786400
C	-4.43082600	-2.84345200	1.78533800
H	-4.69613300	-3.81727200	1.36711900
C	2.11492500	3.47029500	-1.06330400
H	2.81335800	3.99200900	-0.40517400
C	2.49283500	3.13773500	-2.36268500
H	3.48706100	3.40333900	-2.72859400
C	1.60280300	2.45392100	-3.18962800
H	1.89762900	2.18203800	-4.20607900
H	0.54994500	3.37044000	0.43014800
H	-0.35135700	1.53416500	-3.35065600
H	-3.41608600	0.60320900	3.24993300
H	-3.94919800	-1.98326600	-0.14250500
H	1.34232000	-4.62315600	-1.53213400
H	1.34041400	-0.49965400	-2.79762900
H	2.23333600	1.52272000	1.50017800
H	5.34430500	-1.31597300	2.46235100

I^{1}DFT

Ga	0.60766200	0.90687100	0.06154100
P	-1.25517500	-1.65835400	0.42164600
P	-0.51945400	-0.49586600	-1.26176400
N	-0.51621600	-3.26728500	0.29552700

N	2.55210600	1.32515300	0.03007700
N	-2.71766700	-2.37259600	-0.20511500
N	0.11479900	2.80893700	0.45979100
C	3.04623700	2.24240500	0.86096500
C	-1.02015200	-3.92573200	-0.90215400
H	-0.82367600	-5.00854400	-0.86328100
H	-0.54381700	-3.52043300	-1.81565700
C	2.23867600	3.18425500	1.53748100
H	2.75522100	3.82549300	2.25130300
C	-2.52538800	-3.63792800	-0.90752300
H	-2.89984300	-3.58747000	-1.94451200
H	-3.07997200	-4.43930800	-0.38603100
C	0.90685600	3.54867300	1.23445100
C	4.53868700	2.30180300	1.10746200
H	4.98021100	1.29459800	1.11359500
H	4.75397000	2.80177000	2.06185000
H	5.04638800	2.86728200	0.31112800
C	0.39167600	4.83967900	1.83623600
H	0.55769100	5.68758000	1.15404800
H	0.92204100	5.06244800	2.77297900
H	-0.68884100	4.78881900	2.03092100
N	0.64947200	0.35652300	2.12761900
H	0.76916900	1.14583400	2.75491600
H	-0.22980800	-0.13454600	2.30237000
H	1.39828800	-0.31327800	2.28008200
C	-3.97350900	-1.80263700	-0.09073200
C	-4.17284900	-0.62063500	0.66375400
C	-5.10875100	-2.37984000	-0.70559300
C	-5.43643800	-0.04949900	0.78854200
H	-3.32037500	-0.16444700	1.16645400
C	-6.36988700	-1.79296300	-0.57523000
H	-5.01344400	-3.29178000	-1.29434600
C	-6.55226300	-0.62373600	0.16677000
H	-5.55070600	0.85844900	1.38824100
H	-7.22454200	-2.26562300	-1.06761700
H	-7.54143500	-0.17058500	0.26325000
C	0.79970300	-3.44810400	0.70301400
C	1.21284300	-2.96133300	1.96844400
C	1.75970900	-4.12001000	-0.08288300
C	2.52887900	-3.10751500	2.40359000
H	0.46265400	-2.51184800	2.62132000
C	3.07060500	-4.28335200	0.37557000
H	1.48856300	-4.50486100	-1.06580900
C	3.47641000	-3.76639600	1.60808200
H	2.81316600	-2.73022700	3.39067100
H	3.78979200	-4.81496500	-0.25397100
H	4.50578700	-3.88746300	1.95206500
C	-1.10260900	3.31821000	-0.07598200
C	-1.10327200	4.45452300	-0.90058400
C	-2.30383100	2.62741500	0.14002700
C	-2.29358400	4.90762100	-1.47249400
H	-0.16030200	4.96498900	-1.10894500
C	-3.49101100	3.07709600	-0.44150600
H	-2.30350100	1.72572000	0.75085100
C	-3.49170000	4.22160500	-1.24379600
H	-2.28140200	5.79176000	-2.11506900
H	-4.41099700	2.51173000	-0.27766200
H	-4.41997000	4.56922200	-1.70350000
C	3.40033400	0.58056000	-0.84266600
C	3.47070500	-0.81598600	-0.73888100
C	4.11313500	1.23728800	-1.85785500
C	4.27188900	-1.53847100	-1.62681300
H	2.90190800	-1.34134500	0.02975500
C	4.91191700	0.50937700	-2.74114100
H	4.02014700	2.32134100	-1.95757900
C	4.99651800	-0.88244400	-2.62541100
H	4.31737700	-2.62468000	-1.53213300
H	5.46037900	1.03021400	-3.53017100
H	5.61711300	-1.45425100	-3.31969500

2c^{DFT}

Ga	0.96013600	-0.78275200	0.24867100
P	-1.23308700	1.39456600	-0.27722700
P	-0.17327900	0.19267500	-1.53954500
N	-2.78488900	1.90880500	-0.72402700
N	0.06780800	-2.49404700	0.75793800
N	-0.73644900	3.02084400	0.01101100

N	2.62170900	-1.68052300	-0.39690000
C	0.56804600	-3.70326500	0.55320400
C	-3.02619500	3.33320700	-0.45725400
H	-4.00626900	3.49330200	0.01467900
H	-3.00423900	3.90197800	-1.40362000
C	1.85370400	-3.93684200	0.01578600
H	2.14275800	-4.98230500	-0.07788100
C	-1.89703800	3.77692200	0.47116200
H	-1.69946000	4.85697700	0.39784700
H	-2.16260200	3.54762000	1.52309700
C	2.81068100	-2.99815400	-0.41550500
C	-0.27320800	-4.90663400	0.91805800
H	-0.63255700	-4.82493100	1.95537600
H	0.28889100	-5.84238600	0.80489200
H	-1.17077200	-4.95519400	0.28259500
C	4.12853400	-3.52894700	-0.93721000
H	4.33768800	-3.12324300	-1.93909100
H	4.12909500	-4.62508300	-0.98825400
H	4.96064300	-3.20638000	-0.29274300
H	-1.37439300	0.88278100	1.03634400
N	1.23489200	0.12822500	1.87005800
H	1.79747900	-0.37840300	2.54341300
H	1.61320900	1.06345400	1.78896100
C	-1.26993800	-2.29244000	1.21277300
C	-1.51087800	-1.90946500	2.53931200
C	-2.33598600	-2.34587400	0.30196000
C	-2.81161800	-1.60896200	2.95459800
H	-0.66770900	-1.82414500	3.22632500
C	-3.63248000	-2.04031600	0.71950000
H	-2.12963000	-2.59034200	-0.74111100
C	-3.87403500	-1.67309200	2.04733000
H	-2.99121500	-1.30966700	3.99050200
H	-4.44998100	-2.05829800	-0.00338600
H	-4.88809600	-1.42122100	2.36766600
C	3.65206100	-0.77379000	-0.78356800
C	4.65771700	-0.41427400	0.12673900
C	3.61711100	-0.16483500	-2.04676700
C	5.63364500	0.51772600	-0.23361600
H	4.65577500	-0.86695200	1.12066000
C	4.59272900	0.77107600	-2.40186400
H	2.81385500	-0.42501500	-2.73852700
C	5.60706200	1.11089400	-1.50052200
H	6.41303000	0.78960900	0.48291300
H	4.55659000	1.23925900	-3.38875200
H	6.36823700	1.84335900	-1.78049600
C	0.54947000	3.29196100	0.54460000
C	1.69242000	2.98303300	-0.21728900
C	0.71546000	3.85462700	1.82161800
C	2.96748500	3.18709300	0.31042000
H	1.56662400	2.56569700	-1.21518400
C	1.99756100	4.08018600	2.33254100
H	-0.15492400	4.09913700	2.43220300
C	3.12880900	3.73592600	1.58772700
H	3.83960300	2.91076100	-0.28571900
H	2.10817500	4.51522600	3.32937600
H	4.12929100	3.89894400	1.99582400
C	-3.84569900	1.05187000	-1.11720700
C	-5.03776600	1.01411400	-0.37324000
C	-3.72824000	0.23425000	-2.25296200
C	-6.09448700	0.19005600	-0.76422400
H	-5.12961300	1.62325000	0.52851700
C	-4.77800500	-0.61038700	-2.62366100
H	-2.80838600	0.26531200	-2.83657800
C	-5.96793700	-0.63198000	-1.88913000
H	-7.01513600	0.17639900	-0.17494800
H	-4.66844400	-1.24456800	-3.50720200
H	-6.79162300	-1.28347700	-2.19132800

3c^{DFT}

Ga	-1.21266100	0.35965300	0.27198200
P	2.27489500	0.15432900	-0.10118000
P	0.44122700	0.65284300	-1.38861300
N	3.28994400	1.50730100	-0.39982700
N	-2.91327700	0.15587100	-0.76043500
N	3.21569100	-0.79598800	-1.19858700
N	-1.29664000	-1.50933700	0.93861600
C	-3.14943200	-0.99292400	-1.38295800

C	4.22954300	1.33516100	-1.50502600
H	5.26099100	1.26086200	-1.11582700
H	4.18893800	2.19309100	-2.19682300
C	-2.47796700	-2.19139600	-1.05553900
H	-2.69054800	-3.04786300	-1.69423200
C	3.82247800	0.04698900	-2.22435300
H	3.10472300	0.26334900	-3.03893400
H	4.69913100	-0.45333000	-2.66264800
C	-1.73369700	-2.46264700	0.10953500
C	-4.19108300	-1.03264800	-2.47704800
H	-5.18914500	-0.81513600	-2.06622000
H	-4.22133900	-2.01302800	-2.96914300
H	-3.98777600	-0.25581100	-3.23023600
C	-1.44824500	-3.91940400	0.40818100
H	-0.58454400	-4.03621800	1.07402600
H	-1.24501500	-4.45058900	-0.53265900
H	-2.31281000	-4.40493900	0.88728200
N	-1.22330000	1.53138600	1.72332900
H	-1.98323300	1.44248700	2.38584000
H	-1.10949000	2.50919700	1.49053100
H	0.57484500	2.04828600	-1.14109100
C	-3.68971200	1.32144700	-1.01403400
C	-4.90178100	1.52538000	-0.33871400
C	-3.21120100	2.30909000	-1.88851000
C	-5.63394700	2.69673300	-0.54783800
H	-5.25596000	0.75894400	0.35476500
C	-3.94577500	3.48062000	-2.09187900
H	-2.26251300	2.14584500	-2.40536900
C	-5.15913600	3.67762200	-1.42511100
H	-6.57788300	2.84696600	-0.01740900
H	-3.56600600	4.24378700	-2.77597900
H	-5.73093700	4.59506200	-1.58448400
C	-0.91602700	-1.81331600	2.27550400
C	-1.81931600	-2.45752600	3.13784200
C	0.33612300	-1.41791800	2.76973200
C	-1.46302400	-2.73244000	4.45963500
H	-2.80889700	-2.73013000	2.76409400
C	0.68628100	-1.69281800	4.09333400
H	1.03583500	-0.89620600	2.11495500
C	-0.20622200	-2.35416400	4.94300100
H	-2.17605300	-3.23586100	5.11787300
H	1.66751100	-1.38273800	4.46140700
H	0.07180900	-2.56510600	5.97851300
C	3.22527800	2.68153000	0.34471400
C	4.15135300	3.72714400	0.14857800
C	2.21559000	2.85572100	1.31995600
C	4.06768000	4.89887400	0.90543600
H	4.94890800	3.62916400	-0.58821000
C	2.15055300	4.02822200	2.07000300
H	1.46996800	2.07692600	1.49822100
C	3.07163500	5.06331100	1.87080900
H	4.79995200	5.69272900	0.73412200
H	1.35967800	4.12976700	2.81810800
H	3.01206500	5.98247700	2.45800600
C	2.84207600	-2.11870200	-1.48078300
C	2.31180400	-2.93675700	-0.45748400
C	2.99225500	-2.67405800	-2.76635500
C	1.95406200	-4.25728600	-0.71828500
H	2.19536400	-2.53412600	0.55062400
C	2.63341600	-4.00288000	-3.01277600
H	3.37995200	-2.07082900	-3.58705800
C	2.11069800	-4.80543400	-1.99744300
H	1.55728700	-4.87017600	0.09468900
H	2.76076100	-4.40836000	-4.02017000
H	1.83263900	-5.84310300	-2.19553200

TS1

Ga	0.14759900	-1.02657000	-0.40339900
P	-0.38065200	2.00906800	-0.05029600
P	-0.16867700	0.46586400	1.31383200
N	0.67904600	3.38425400	0.06777000
N	1.81076000	-2.12901500	-0.38788700
N	-1.72194600	3.07322400	0.20740100
N	-0.99956500	-2.66220800	-0.39973400
C	1.83760700	-3.18295000	-1.20283800
C	0.13941800	4.32281400	1.02676100
H	0.59248200	5.31588700	0.88627800

H	0.33458900	4.00715000	2.07098200
C	0.67169600	-3.76433300	-1.73716700
H	0.82389500	-4.54840200	-2.47977200
C	-1.36005500	4.36971100	0.74451600
H	-1.90879500	4.59319200	1.67461300
H	-1.60109400	5.16239800	0.01192100
C	-0.63139600	-3.64898300	-1.21757500
C	3.15013800	-3.81553400	-1.58826300
H	3.97285600	-3.08813900	-1.55978400
H	3.07131200	-4.23929400	-2.59904300
H	3.41507200	-4.63995600	-0.90892000
C	-1.61946000	-4.71180200	-1.62574400
H	-1.56406900	-5.59049200	-0.96534400
H	-1.38749100	-5.05556500	-2.64342800
H	-2.65207600	-4.33877900	-1.59314000
N	-0.00851700	-0.28648100	-2.17260600
H	-0.75188700	-0.70218800	-2.73203000
H	-0.31634700	1.40996900	-1.51333300
H	0.84169500	-0.35737500	-2.72960200
C	-2.15782700	-2.77267400	0.40393900
C	-2.32124700	-3.85501300	1.28078900
C	-3.13103500	-1.76723200	0.38500500
C	-3.45079000	-3.94503300	2.08946200
H	-1.54046500	-4.61691800	1.34033100
C	-4.25778500	-1.85849300	1.19809400
H	-3.00142800	-0.90313400	-0.26643400
C	-4.42704700	-2.94954700	2.04887900
H	-3.56097200	-4.79419100	2.76874800
H	-5.00310100	-1.05996300	1.16518700
H	-5.30960500	-3.01780500	2.68917500
C	2.92112700	-1.80790200	0.42820300
C	3.48257900	-0.52696400	0.38966200
C	3.43500600	-2.74707500	1.33397700
C	4.55668900	-0.20349000	1.21551600
H	3.07687000	0.22362800	-0.28963700
C	4.50996000	-2.42050900	2.15588000
H	2.96770000	-3.73224700	1.40436900
C	5.07876600	-1.14812600	2.09751400
H	4.97958100	0.80278500	1.16416600
H	4.89697000	-3.16320300	2.85822400
H	5.91868300	-0.89093000	2.74718800
C	-3.03477200	2.77342000	-0.12836800
C	-3.34016600	1.68296100	-0.96732000
C	-4.10254400	3.56916500	0.32779100
C	-4.65686100	1.38857200	-1.30334700
H	-2.53184400	1.07414600	-1.37723800
C	-5.41606400	3.26723700	-0.02291100
H	-3.91114900	4.42999500	0.96885600
C	-5.71034000	2.17306800	-0.83298400
H	-4.85959000	0.53554200	-1.95662400
H	-6.22252300	3.90391200	0.35046600
H	-6.74235600	1.94105200	-1.10410700
C	2.03791800	3.33619800	-0.22657500
C	2.49270500	2.61334600	-1.34706900
C	2.98559500	4.03187800	0.54360900
C	3.84290400	2.59374900	-1.67750500
H	1.77208100	2.08176200	-1.97241200
C	4.33462600	4.01400300	0.19442500
H	2.67112500	4.58538600	1.42948800
C	4.77762700	3.29474900	-0.91326900
H	4.16852600	2.03019200	-2.55585800
H	5.04954800	4.56785400	0.80869500
H	5.83588400	3.28373400	-1.18274400

TS2

Ga	-0.32486500	-1.34645800	-0.29861900
P	0.66096600	2.11190300	0.51749700
P	0.38214900	0.63721600	-1.06359600
N	-0.36074000	3.47639400	0.08030400
N	0.70585900	-2.82060700	0.49135700
N	2.02220700	3.05451300	-0.07188500
N	-2.04622300	-1.97242000	0.39611100
C	0.28076400	-3.47234600	1.57381800
C	0.16008100	4.14504000	-1.08876700
H	-0.25648500	5.16023100	-1.17891000
H	-0.09152600	3.58778200	-2.01472500
C	-1.02544800	-3.35892200	2.08154700

H	-1.22345400	-3.89633900	3.00806300
C	1.67089000	4.19956400	-0.88436300
H	2.18977200	4.18173100	-1.85952400
H	1.96238200	5.13546600	-0.37043600
C	-2.13517200	-2.73782500	1.48288900
C	1.22629100	-4.37986400	2.31347400
H	1.28618600	-5.36873900	1.83390400
H	0.88112300	-4.52849900	3.34442300
H	2.24460000	-3.96609200	2.32545700
C	-3.48097700	-2.95965600	2.11604700
H	-4.05600400	-2.02283400	2.15153500
H	-3.36921600	-3.35820800	3.13185900
H	-4.07805100	-3.67642100	1.53195900
N	-0.34471800	-1.53280800	-2.34723500
H	-1.23528200	-1.60092100	-2.84063400
H	0.00564400	-0.37900200	-2.22464600
H	0.35444400	-2.10238500	-2.82463400
C	-3.20664300	-1.51964200	-0.29000900
C	-3.97604300	-2.42225600	-1.03701800
C	-3.54476400	-0.16194800	-0.28869000
C	-5.08677100	-1.97528200	-1.74870100
H	-3.68853700	-3.47664600	-1.06107300
C	-4.65681900	0.27852100	-1.00587400
H	-2.93642800	0.55080700	0.27138800
C	-5.43155800	-0.62316600	-1.73329800
H	-5.68214100	-2.68781200	-2.32529700
H	-4.90802900	1.34159100	-0.98683400
H	-6.30126800	-0.27294000	-2.29430400
C	1.95959300	-3.11373600	-0.10931600
C	2.18729300	-4.36418300	-0.70092200
C	2.95163800	-2.12831100	-0.18512300
C	3.40249000	-4.63519900	-1.32492900
H	1.39726600	-5.11903800	-0.67818300
C	4.16347400	-2.40510900	-0.81619100
H	2.76281500	-1.13814800	0.23299500
C	4.39652300	-3.65757600	-1.38159200
H	3.56940800	-5.61511400	-1.77931900
H	4.92646600	-1.62425000	-0.86365500
H	5.34791400	-3.87012800	-1.87488200
C	3.33715700	2.74477000	0.20059300
C	3.67322800	1.64059400	1.01612000
C	4.39892000	3.51642100	-0.31716600
C	4.99904900	1.32829800	1.28844600
H	2.87266200	1.03678100	1.44975500
C	5.72315600	3.18854100	-0.03602600
H	4.19175200	4.38372700	-0.94462500
C	6.04264200	2.09365200	0.76346700
H	5.21915600	0.46921100	1.92870600
H	6.51993900	3.80980200	-0.45452600
H	7.08344000	1.84332500	0.97941200
C	-1.70523700	3.52039900	0.40823300
C	-2.16302900	2.94421500	1.61377800
C	-2.65557400	4.15020300	-0.41837900
C	-3.50353600	3.00725400	1.97061100
H	-1.44002700	2.45970500	2.27429900
C	-3.99609100	4.21744500	-0.04084000
H	-2.34909600	4.58764100	-1.36925200
C	-4.43736800	3.64693900	1.15071400
H	-3.82328300	2.55834600	2.91509000
H	-4.70688700	4.72211900	-0.70133600
H	-5.48882900	3.70214900	1.44085900

6[DFT]

Ga	-1.73082400	0.55948000	-1.05649000
N	1.62370900	-1.81259500	1.87496700
N	2.54311300	-1.28560000	-0.32122800
N	-3.01225400	1.92710200	-0.38936600
N	-3.00802400	-0.95917900	-0.89807600
P	0.95148700	-1.57481400	0.29510000
P	0.01689000	0.50574300	0.54046000
Si	1.33465000	1.88609600	-0.64803100
C	2.87435900	-1.07827800	2.03284100
H	3.46877200	-1.50540300	2.85447900
H	2.69567800	-0.01056000	2.25417000
C	3.59189700	-1.23316700	0.69736500
H	4.27074200	-0.38572800	0.52406200
H	4.18207900	-2.16761000	0.67567300

C	0.79157400	-2.04969400	2.97949900
C	1.02583300	-1.44952000	4.23095700
C	0.18632800	-1.72318400	5.31581700
H	0.38881800	-1.24470100	6.27794700
C	-0.90523800	-2.58261700	5.17994300
H	-1.56008400	-2.78799300	6.02989100
C	-1.14293000	-3.18137900	3.93597400
H	-1.98512500	-3.86802000	3.81091300
C	-0.30559900	-2.92775400	2.85266600
C	2.85097500	-1.34323100	-1.67714300
C	1.82195300	-1.42551500	-2.64226500
C	2.12077400	-1.46332300	-4.00136800
H	1.30050300	-1.51995400	-4.72217800
C	3.44723400	-1.41702300	-4.44736500
H	3.67730100	-1.44084600	-5.51485100
C	4.46991400	-1.33523900	-3.49957700
H	5.51371500	-1.29843900	-3.82364000
C	4.18574600	-1.30024100	-2.13145100
C	-4.29263900	1.69215300	-0.13912500
C	-4.89942700	0.42803300	-0.29559500
H	-5.97123900	0.39585700	-0.10719400
C	-4.30323100	-0.80568400	-0.62161800
C	-5.15773300	2.83795900	0.33563600
H	-4.79716800	3.21315100	1.30574300
H	-6.20665100	2.53452700	0.44310700
H	-5.09782400	3.68441900	-0.36555700
C	-5.22071700	-2.01091600	-0.66524500
H	-4.95264000	-2.68616200	-1.49037300
H	-6.26558600	-1.69569900	-0.78426300
H	-5.14950800	-2.59662700	0.26402800
C	-2.42627800	3.20984700	-0.16570300
C	-2.26149800	4.09875200	-1.23681200
C	-1.61013800	5.31926400	-1.03899900
H	-1.48175400	6.00622700	-1.87914700
C	-1.11320000	5.65506400	0.22402000
H	-0.59520500	6.60526700	0.37462100
C	-1.27073700	4.76454900	1.29116800
H	-0.87418300	5.01621400	2.27801100
C	-1.92238100	3.54388500	1.09977700
C	-2.43064400	-2.24371400	-1.11493300
C	-1.80470500	-2.51954400	-2.34100800
C	-1.15685900	-3.74078900	-2.54190900
H	-0.65977700	-3.93671100	-3.49493900
C	-1.13694000	-4.70391000	-1.52929100
H	-0.62677800	-5.65695100	-1.68665900
C	-1.76432200	-4.43387600	-0.30909100
H	-1.74193100	-5.17597600	0.49325700
C	-2.39559300	-3.20739500	-0.09566400
C	3.01230800	2.16537700	0.14423800
C	3.14125600	2.52030600	1.50062900
H	2.24362700	2.64060700	2.11527300
C	4.39881300	2.70893300	2.08080400
H	4.47839900	2.98187000	3.13672700
C	5.55669500	2.54630700	1.31058100
H	6.54128100	2.69101000	1.76341000
C	5.44847300	2.19834300	-0.03941000
H	6.34900100	2.06710100	-0.64563100
C	4.18691900	2.00862900	-0.61519700
H	4.11733100	1.71423400	-1.66581500
H	-1.40383300	0.83121000	-2.57751300
H	1.59143800	1.44108600	-2.03949900
H	0.59659300	3.17522900	-0.70876500
H	5.00924700	-1.23294500	-1.42128700
H	0.77948000	-1.44993900	-2.32242600
H	-2.63612400	3.81513300	-2.22285000
H	-2.02778200	2.82884800	1.91786000
H	-2.83928400	-2.97433100	0.87398100
H	-1.82657800	-1.76627200	-3.13136600
H	-0.48211600	-3.41689700	1.89500900
H	1.85467800	-0.75393500	4.36308700

[1,3]

Ga	1.25601200	-1.13884300	0.28847400
P	-1.33871100	0.67710800	-0.90247900
P	-0.09924600	-0.81576500	-1.58836300
N	-2.85739200	0.69746600	-1.73163200
N	0.91948900	-2.96260600	1.04583600

N	-1.05666300	2.33329500	-1.35207600
N	3.05572000	-1.64299400	-0.41876300
C	1.61714600	-4.04685100	0.73330400
C	-3.63998200	-0.43824600	-1.98807200
C	0.02616700	3.10609000	-0.87466000
C	-3.09162300	1.93180900	-2.47237000
H	-4.16557700	2.17358700	-2.47954400
H	-2.74609300	1.84616700	-3.51911600
C	2.79640400	-4.01815700	-0.03871000
H	3.25921900	-4.98409700	-0.23269700
C	-2.29511700	2.99684700	-1.73740700
H	-2.07478000	3.85524800	-2.39014500
H	-2.86228200	3.37007500	-0.86259200
C	-3.63015700	-1.54309500	-1.11150700
C	1.32984400	2.57564600	-0.84332300
C	3.48510600	-2.89686800	-0.54117100
C	-0.25468100	-3.03635700	1.84950800
C	3.85551100	-0.55496700	-0.88246300
C	-0.16758900	4.43262600	-0.44535500
C	-4.48096800	-0.49570200	-3.11714700
C	-1.50107600	-3.30939400	1.26582700
C	2.39303200	3.33387000	-0.35409400
H	3.38981800	2.88918000	-0.33830500
C	3.63640400	-0.00388900	-2.15384500
C	-2.58490800	-2.97891200	3.41124200
H	-3.49384800	-2.95035600	4.01693700
C	1.14002000	-5.38827100	1.24441400
H	1.05066300	-5.37211800	2.34172300
H	1.82357700	-6.19653100	0.95557700
H	0.13611200	-5.61600700	0.85461100
C	-0.18197600	-2.72917500	3.21573600
C	-4.42180900	-2.66080800	-1.36675700
H	-4.38897900	-3.50518000	-0.67298300
C	-5.25487900	-2.71200000	-2.48969200
H	-5.87558200	-3.58985500	-2.68282000
C	4.79769500	-3.14522600	-1.25228700
H	4.78379800	-2.69652000	-2.25730100
H	5.00802200	-4.21825700	-1.34289400
H	5.62894800	-2.66759300	-0.71131900
C	4.83525000	0.00166100	-0.04680200
C	-5.27809500	-1.61855800	-3.35690700
H	-5.91856100	-1.63476300	-4.24285900
C	0.90805500	5.19149400	0.01985400
H	0.72733000	6.21476300	0.35891800
C	2.19301400	4.64778900	0.08146900
H	3.02981500	5.24214100	0.45635400
C	-2.65892500	-3.28340600	2.04807700
H	-3.62826700	-3.48755100	1.58729600
C	-1.34299200	-2.70370100	3.99278000
H	-1.27771500	-2.45826500	5.05577400
C	4.40984600	1.07512400	-2.59090600
H	4.22881200	1.50160700	-3.58049500
C	5.40386500	1.61240100	-1.76687100
H	6.00545300	2.45683600	-2.11176000
C	5.61099700	1.07555500	-0.49160700
H	6.37607200	1.49904100	0.16424500
H	1.42413600	-0.12447500	1.48295500
Si	-1.92704900	0.75886400	1.33923800
H	-1.32890400	-0.39251900	2.03960000
H	-3.40794100	0.65535300	1.34488900
C	-1.42901500	2.38211400	2.10417700
C	-2.36600100	3.42391200	2.24006600
C	-0.10411500	2.61034100	2.52450000
C	-1.99272900	4.65796600	2.78135800
H	-3.40256700	3.26968200	1.92474000
C	0.26867900	3.84078000	3.06724100
H	0.64734600	1.82518800	2.40764300
C	-0.67435800	4.86614400	3.19686500
H	-2.73251900	5.45671100	2.88205900
H	1.30364000	4.00596700	3.37538400
H	-0.37889700	5.83131800	3.61679200
H	-1.16428000	4.87217200	-0.43361400
H	-2.99725400	-1.53314600	-0.22694200
H	-4.50951000	0.32899300	-3.82902800
H	4.97961700	-0.41969300	0.95072200
H	0.79121400	-2.49409100	3.65210300

H	-1.54952100	-3.51300900	0.19448900
H	2.84799900	-0.42156900	-2.78282200
H	1.50883300	1.57381900	-1.22531700

TS3

Ga	0.09281600	1.24302800	-0.79553700
P	0.60110600	-1.80775500	-0.54415200
P	0.66300100	-0.46513000	-2.16594300
N	1.97076700	-2.88185200	-0.53122400
N	1.24380600	2.76615500	-0.31180200
N	-0.42919300	-3.19903300	-0.77712200
N	-1.49318400	2.35049500	-1.18560900
C	0.92924700	4.04017200	-0.54705000
C	3.30198800	-2.47353800	-0.33114900
C	-1.75532200	-3.25914100	-0.37196800
C	1.69797200	-3.98252600	-1.43754600
H	2.42856900	-4.79054200	-1.28119300
H	1.74247300	-3.67686000	-2.50270800
C	-0.31599600	4.45583200	-1.04206900
H	-0.42093900	5.52458100	-1.22094900
C	0.29433200	-4.41953500	-1.07064600
H	-0.18205100	-4.97435900	-1.89687600
H	0.32319200	-5.07916000	-0.18277300
C	3.71356000	-2.04303100	0.93845800
C	-2.49133400	-2.09212700	-0.08947300
C	-1.45165500	3.67829800	-1.31374200
C	2.50781900	2.44917500	0.26546500
C	-2.71722200	1.66541500	-1.44325500
C	-2.40701300	-4.49955200	-0.21546100
C	4.25778800	-2.56874400	-1.35357900
C	3.51783100	1.88591900	-0.52078300
C	-3.79521900	-2.17054100	0.38776200
H	-4.32501100	-1.24340500	0.61359600
C	-2.87736000	0.93416900	-2.62512700
C	4.99310600	1.85169600	1.39482400
H	5.96640300	1.62049300	1.83392200
C	1.95475000	5.11059500	-0.29067700
H	2.11794100	5.25026000	0.78834000
H	1.63182000	6.06701300	-0.71878800
H	2.92784500	4.82912300	-0.71925500
C	2.73414700	2.67358100	1.62786200
C	5.04591400	-1.72424700	1.17807500
H	5.34696600	-1.40239100	2.17800800
C	5.99655500	-1.83923000	0.16291200
H	7.04639400	-1.60831500	0.35977100
C	-2.67535800	4.40995800	-1.79504200
H	-3.10033800	3.91870100	-2.68271700
H	-2.43280800	5.45120000	-2.03823700
H	-3.46491300	4.40668000	-1.02897600
C	-3.76092600	1.70985100	-0.51141500
C	5.59383100	-2.26086800	-1.10286800
H	6.32625200	-2.35225400	-1.90936000
C	-3.71982400	-4.56082900	0.24311700
H	-4.19327200	-5.53957200	0.35935800
C	-4.42371700	-3.40222200	0.56346800
H	-5.44897200	-3.45706300	0.93560000
C	4.75692900	1.59493900	0.04542300
H	5.53828800	1.14820200	-0.57283900
C	3.97610900	2.38275800	2.18696400
H	4.14552800	2.56507700	3.25101700
C	-4.08541500	0.28985800	-2.88630600
H	-4.20069300	-0.28102100	-3.81055700
C	-5.13776100	0.36750800	-1.97625600
H	-6.08289900	-0.13846300	-2.18577200
C	-4.97015100	1.07501400	-0.78619700
H	-5.78262100	1.12439800	-0.05686900
H	-0.15265600	0.36669800	0.69030400
Si	0.13523400	-0.81587000	1.63721500
H	1.47133700	-0.26673200	2.04141000
H	0.14190500	-2.24187800	2.18112100
C	-1.26622100	-0.12095200	2.74257900
C	-1.96404000	-0.97556700	3.60662300
C	-1.59793900	1.24108200	2.75612800
C	-2.95459600	-0.48728400	4.45956100
H	-1.72809400	-2.04409600	3.60544900
C	-2.57582200	1.74061300	3.61569500
H	-1.07986300	1.93174300	2.08048300

C	-3.25982500	0.87310600	4.46866900
H	-3.49191800	-1.17306600	5.12014000
H	-2.80754600	2.80951300	3.62124500
H	-4.03283400	1.25853900	5.13886400
H	-1.88487200	-5.42929900	-0.44033300
H	2.97915700	-1.99776200	1.74419100
H	3.95317300	-2.88740300	-2.35204900
H	-3.61596400	2.24251900	0.43148300
H	1.92636100	3.07278100	2.24631200
H	3.32093200	1.67199500	-1.57317500
H	-2.04665400	0.86883600	-3.33031600
H	-2.05213700	-1.11109600	-0.28239100

TS4

Ga	1.71048600	0.38093300	-0.63453500
N	-1.87717800	-0.58087400	2.62823300
N	-2.89416400	0.10554100	0.52861200
N	3.50736400	-0.39953400	-0.72023300
N	2.28227400	2.22618600	-0.39243000
P	-1.39568900	0.46303200	1.31898000
P	0.10836000	-0.94950900	0.35923800
Si	-0.37582500	-0.93481700	-1.90977100
C	-2.68144500	-1.67107300	2.11412500
H	-3.26672300	-2.13484900	2.92271800
H	-2.04729800	-2.45435200	1.65209600
C	-3.60109400	-1.04267400	1.07482400
H	-3.85474400	-1.77011800	0.28545900
H	-4.53923700	-0.70036600	1.54944300
C	-1.09900900	-0.72505600	3.77391300
C	-1.01347000	-1.94745900	4.46265400
C	-0.26041800	-2.05236000	5.63117600
H	-0.21517800	-3.01514200	6.14730100
C	0.43789300	-0.95896100	6.13549400
H	1.02945200	-1.04897400	7.04907300
C	0.36720300	0.25596800	5.45026000
H	0.90258100	1.13007200	5.83075500
C	-0.38944000	0.37724500	4.29227900
C	-3.52933600	0.97248300	-0.35188200
C	-2.89050100	2.13613800	-0.82385200
C	-3.53976700	2.99939300	-1.69765300
H	-3.01177200	3.89195300	-2.04247100
C	-4.83854200	2.73883000	-2.13660800
H	-5.34189800	3.41908200	-2.82688500
C	-5.47525700	1.58832500	-1.67894000
H	-6.49119300	1.35631800	-2.00996000
C	-4.83817000	0.71321800	-0.80215400
C	4.62150500	0.29562600	-0.52334300
C	4.64022100	1.68317700	-0.28795900
H	5.62465500	2.12202300	-0.13503900
C	3.56420300	2.58023600	-0.25121500
C	5.94177400	-0.42185900	-0.54804800
H	5.92269500	-1.28880200	0.12901300
H	6.75961900	0.24713800	-0.25613600
H	6.15075700	-0.81774900	-1.55321200
C	3.88717100	4.03334400	-0.04085000
H	3.52872100	4.63736200	-0.88788100
H	4.96707300	4.18332600	0.07085300
H	3.37683300	4.42153800	0.85315100
C	3.55548100	-1.79533300	-1.00852500
C	3.79350700	-2.23469300	-2.31505500
C	3.79692600	-3.59723800	-2.60302600
H	3.97949300	-3.93267100	-3.62670800
C	3.55240900	-4.52811200	-1.59384800
H	3.54774800	-5.59619100	-1.82313900
C	3.29963400	-4.08992300	-0.29511600
H	3.09375700	-4.81266400	0.49791700
C	3.29612200	-2.72786900	0.00076500
C	1.26509400	3.22715000	-0.38106800
C	0.85982700	3.82481900	-1.57923800
C	-0.12255900	4.81324800	-1.56848200
H	-0.42900200	5.27928000	-2.50813700
C	-0.71572200	5.20066600	-0.36726700
H	-1.48998800	5.97109700	-0.36050100
C	-0.33111100	4.58654000	0.82380600
H	-0.80826900	4.86857900	1.76507800
C	0.65344200	3.60057400	0.81973300
C	-1.92325200	-2.10535900	-1.94601800

C	-1.89225900	-3.38370700	-1.36290900
H	-0.98562500	-3.71403900	-0.84230600
C	-2.98724700	-4.24488700	-1.42855300
H	-2.93467500	-5.23541200	-0.96692700
C	-4.15208900	-3.84373300	-2.08740900
H	-5.01285100	-4.51589400	-2.14246600
C	-4.20604600	-2.58183400	-2.67692200
H	-5.11278400	-2.25784100	-3.19603300
C	-3.10208000	-1.72768100	-2.60323500
H	-3.16659200	-0.73660900	-3.06539600
H	0.89755900	0.05608200	-2.05835100
H	-0.98877200	0.26190800	-2.58425600
H	0.57610200	-1.81555700	-2.67578300
H	-5.36172700	-0.18679300	-0.48144400
H	-1.87298600	2.36899400	-0.50029700
H	3.96023000	-1.49800900	-3.10461800
H	3.07838000	-2.37697200	1.01150000
H	0.95313200	3.10777700	1.74719800
H	1.32462400	3.50951800	-2.51640700
H	-0.45528400	1.33906800	3.77749200
H	-1.53004700	-2.82868000	4.08099800

4. References

[1] D. W. N. Wilson, J. Feld, J. M. Goicoechea, *Angew. Chem. Int. Ed.* **2020**, *59*, 20914−20918.

[2] *CrysAlisPro*, Agilent Technologies, Version 1.171.35.8.

[3] (a) G. M. Sheldrick in SHELXL97, *Programs for Crystal Structure Analysis (Release 97-2)*, Institut für Anorganische Chemie der Universität, Tammanstrasse 4, D-3400 Göttingen, Germany, 1998; (b) G. M. Sheldrick, *Acta Crystallogr. Sect. A* 1990, **46**, 467−473; (c) G. M. Sheldrick, *Acta Crystallogr. Sect. A* 2008, **64**, 112−122.

[4] M. J. Frisch, G. W. Trucks, H. B. Schlegel, G. E. Scuseria, M. A. Robb, J. R. Cheeseman, G. Scalmani, V. Barone, B. Mennucci, G. A. Petersson, et al., Gaussian 16, Revision B.01, 2016.
